# *Megastigmus* seed chalcids (Hymenoptera, Torymidae) radiated much more on Angiosperms than previously considered. I- Description of 8 new species from Kenya, with a key to the females of Eastern and Southern Africa

**DOI:** 10.3897/zookeys.585.7503

**Published:** 2016-04-26

**Authors:** Alain Roques, Robert S. Copeland, Laurent Soldati, Olivier Denux, Marie-Anne Auger-Rozenberg

**Affiliations:** 1 INRA, UR633, Zoologie Forestière, 2163 Avenue Pomme de Pin, F-45075, Orléans, France; 2 ICIPE, International Centre of Insect Physiology and Ecology, P.O. Box 30772, Nairobi 00100, Kenya; 3 National Museums of Kenya, Division of Invertebrate Zoology, P.O. Box 40658, Nairobi 00100, Kenya; 4 INRA, UMR 1062, Centre de Biologie pour la Gestion des Populations, Campus International de Baillarguet, CS 30016, F-34988, Montferrier-sur-Lez, France

**Keywords:** Chalcidoidea, Megastigmus, seed, Anacardiaceae, Malvaceae, Rhamnaceae, new species, Africa, plant hosts, fruit

## Abstract

A survey of seed chalcids from woody plants in Kenya revealed 12 species belonging to the genus *Megastigmus* Dalman, 1820, and has increased to 16 the number of *Megastigmus* species presently recorded from the Afrotropical Region, of which at least 13 are seed feeders. A key to female *Megastigmus* of the Afrotropical Region is provided. Eight new species are described from morphological evidence: *Megastigmus lanneae* Roques & Copeland, *Megastigmus laventhali* Roques & Copeland, *Megastigmus ozoroae* Roques & Copeland, and *Megastigmus smithi* Roques & Copeland in seeds of species of the family Anacardiaceae, *Megastigmus copelandi* Roques & Copeland and *Megastigmus grewianae* Roques & Copeland in seeds of Malvaceae, *Megastigmus helinae* Roques & Copeland in seeds of Rhamnaceae, and *Megastigmus icipeensis* Roques & Copeland for which no host is known. These collections include the first records of Malvaceae and Rhamnaceae as hosts of *Megastigmus* seed chalcids, which appear to have radiated in Angiosperms much more than previously considered. Analyses of the mitochondrial (cytochrome oxidase subunit one – COI) and nuclear DNA (28S ribosomal region) could be carried out on 8 of the 16 African species of which 5 were newly described ones. The species associated with Anacardiaceae always clustered together in phylogenies, confirming the existence of a strong and ancestral monophyletic clade, unlike the ones associated with Malvaceae and Rhamnaceae, whose position remains unclear. All holotypes are deposited in the National Museums of Kenya.

cytochrome oxidase subunit one –

## Introduction

Globalization, the accelerating worldwide movement of goods and people, has led to an exponential increase in the transport of alien organisms across geographical barriers (DAISIE 2009; Essl et al. 2011), and the international trade in plant propagative material is widely recognized as a major mode of introduction of alien arthropod pests and microbial diseases (Roques et al. 2010; Liebhold et al. 2012). The seed trade, in particular, has been identified as highly prone to pest invasion as a result of the limited measures in place to regulate seed traffic and the specific biology of the associated alien organisms (Auger-Rozenberg and Roques 2012). Indeed, phytosanitary regulations regarding the trade in tree seeds are non-existent in a number of countries, including those of the European Union, whilst the exploding development of trade through the internet allows seeds to move quite freely all over the world. In addition, the endophytic way of life of most insects associated with seeds makes it difficult to detect their presence during the transport and importation processes. In most cases, examining the outside of the seeds does not provide any evidence of insect larvae which can be detected only when seeds are X-rayed (Auger-Rozenberg and Roques 2012). As a result, 11 of the 21 seed wasp species in the genus *Megastigmus* (Hymenoptera, Torymidae) known to date in Europe are of exotic origin (Roques and Skrzypczyńska 2003). This level of invasion has already had negative consequences for both natural regeneration of native trees and seed niche exploitation by native seed feeders (Auger-Rozenberg and Roques 2012). Therefore, it is important to increase our knowledge about the species that may be introduced with tree seeds.

Seeds of woody plants can be infested by larvae of a number of arthropod groups, including, among others, dipteran seed midges in the family Cecidomyiidae (e.g., *Plemeliella*, *Mayetiola*), coleopteran seed beetles in the family Bruchidae, and mites in the genus *Trisetacus* (Nalepellidae) (Křístek et al. 1992). However, the most important group consists of hymenopteran seed chalcids in the families Torymidae (e.g., *Megastigmus*, *Bootanellus*, *Bootania*, *Torymus)*, Eurytomidae (e.g. *Bephratelloides*, *Bruchophagus*, *Eurytoma*, *Risbecoma*, *Striateurytoma*), Pteromalidae (*Homodecatoma*), Eulophidae (*Anselmella*), and Tanaostigmatidae (*Tanaoneura*) (Roques et al. 2003). Among these seed chalcids, the genus *Megastigmus* appears dominant. In his checklist of the world Megastigminae, Grissell (1999) listed 126 *Megastigmus* species of which about one-third were phytophagous in tree seeds, about one-third were parasitoids or inquilines of gall-forming insects in the fruits, leaves and stems of plants, and about one-third had no known hosts. Since 2000, a number of new *Megastigmus* species have been described (Roques and Skrzypczyńska 2003; Auger-Rozenberg et al. 2006; Ghramh and Shati 2011; Grissel 2006; Sureshan 2009; Narendran et al. 2010; Doǧanlar and Hassan 2010; Galindo-González et al. 2012; Doǧanlar et al. 2013; Doǧanlar 2015). Doǧanlar (2011) also transferred the metallic-coloured, parasitic species into the genus *Bootanomyia*.

Data from these recent studies indicate that, worldwide, the genus *Megastigmus* currently includes 135 species, 70 of which are seed feeders, 45 develop in galls, and 20 for which behavioral data is lacking (Noyes 2015). Among the seed feeders, species associated with conifers are most abundant with 49 species (24 spp. in the family Pinaceae; 22 spp. in Cupressaceae; one sp. in Taxodiaceae, and two spp. in unidentified conifers), whereas 21 species have been shown to develop within seeds of five families of Angiosperms (Rosaceae: 13 spp.; Anacardiaceae: four spp.; Fabaceae: two spp.; Aquifoliaceae: one sp.; Hamamelidaceae: one sp.). Although found in all biogeographic regions, most species of *Megastigmus* have been described from the Palearctic, Australian, and Nearctic Regions.

To date, relatively few species have been reported from subtropical and tropical areas. In the Afrotropical region, only seven species have been recognized. Four species develop as phytophages in angiosperm seeds. Three of these, *Megastigmus hypogeus* (Hussey), *Megastigmus thomseni* (Hussey), and *Megastigmus transvaalensis* (Hussey) (= *Megastigmus rhusi* [Hussey]) attack seeds of Anacardiaceae (Hussey 1956a; Grissell and Prinsloo 2001). A fourth species, *Megastigmus aculeatus* (Swederus), is specific to seeds of *Rosa* spp. (Rosaceae) and, in the Afrotropical region, was previously known only from a single record from a US quarantine inspection in French Somaliland (Roques and Skrzypczynska 2003). It has since been collected in the wild in Eastern Cape, South Africa (Simon Van Noort, personal communication, IZIKO catalogue SAM-HYM-P005791). Another species, *Megastigmus somaliensis* Hussey, develops in seeds of *Juniperus procera* Endl. (Cupressaceae) (Hussey 1956b). Two final Afrotropical species, *Megastigmus pretorianensis* Doǧanlar and *Megastigmus zebrinus* Grissell, develop in *Eucalyptus* galls, the former as an inquiline or parasitoid in leaf, stem and petiole galls induced by the eulophid *Leptocybe invasa* Fisher & LaSalle (Doǧanlar 2015) and the latter as a gall maker in seeds of *Eucalyptus camaldulensis* Dehnhardt and *Syzygium cordatum* Hochst. ex Krauss, both Myrtaceae (Grissell 2006). Of these seven species, at least two, *Megastigmus aculeatus* and *Megastigmus zebrinus*, are not native to Africa but were introduced with their host from Eurasia and Australia, respectively. Thus far, *Megastigmus pretorianensis* is known only from South Africa (Doǧanlar 2015). The native status of *Megastigmus transvaalensis* which develops in seeds of the exotic pink pepper trees, *Schinus* spp., introduced from South America, but also in *Rhus* species native to Africa (Grissell and Prinsloo 2001), is still debatable, although molecular studies by Scheffer and Grissell (2003) suggested a South African origin.

The lower diversity of *Megastigmus* seed chalcids observed in angiosperms and in subtropical/ tropical areas may be due to a more limited radiation process in these hosts and regions or simply reflect more limited investigations in the tropics compared to those carried out in conifers of the Holarctic region in relation to afforestation programs and establishment of seed orchards (Hedlin et al. 1980; Roques and Skrzypczyńska 2003). The present paper aims at contributing to the knowledge of African *Megastigmus*. It presents the results of a large collection campaign carried out by the International Centre of Insect Physiology and Ecology (ICIPE) that included, primarily, fruits of Angiosperm trees and shrubs from different regions of Kenya. Between 1999 and 2012 two projects were conducted in this country, both of which focused on the rearing of insects, especially Tephritidae, from wild fruits. The first of these (1999–2004) was a country-wide survey of fruits from diverse habitat types. The second (2011–2012) focused on the northernmost representatives of the Eastern Arc mountains, the Taita Hills and the outlying Kasigau Mountain, both located in southeastern Kenya. Most of the collections from this second project were from highland (ca.1600–2200 m elevation) wet-forest remnants, with a smaller number of samples from mid-altitude dry savanna and woodland.

Over 700 insect species were reared from fruits and much of the data from the two projects has been published previously (e.g. Copeland et al. 2002, White et al. 2003, Copeland et al. 2004, Copeland et al. 2006, Copeland et al. 2009, Adamski et al. 2012, Razowski and Brown 2012, Brown et al. 2014). Surprisingly, *Megastigmus* were reared from several species of wild fruits. *Megastigmus* specimens were also recovered, though rarely, from Malaise trap samples from an unrelated project, also conducted by the second author. In this paper, combining the use of morphological and molecular tools, we report range expansion of some previously described species, add two plant families of Angiosperms to the list of known hosts of seed-feeding *Megastigmus*, describe eight new species from Kenya, and provide a key to females of all known Afrotropical species.

## Material and methods

### Fruit and insect collection and rearing

During the 1999–2004 wild-fruit survey, 3839 fruit collections were made throughout much of Kenya. An additional 347 wild-fruit collections were made during the 2011–2012 survey in the Taita Hills, bringing the total number of fruit samples to 4186, the great majority from woody plants. Over the course of the two surveys approximately 930 species of plants were sampled, representing 122 families. Details of fruit collection and insect rearing methods are available in Copeland et al. (2002) and Copeland (2006). Briefly, fruits were collected from plants or on the ground below them. An effort was made to collect ripe, but not rotting fruit. Binoculars were used to associate fallen fruit under tall trees with fruit still remaining on the tree. Leaf and stem specimens, including flowers when present, were pressed in the field. Photographic vouchers of fruits and leaf and stem specimens were made at the time of collection. A ball point pen was included in each photograph to provide scale. The approximate diameter of the barrel of the pen was 10mm.

During transport, damage to fruits was minimised by placing each fruit sample in a separate polythene bag and suspending the bag above the bottom of a two-litre plastic storage container. A tight-fitting plastic lid fixed the bag and its contents in place. A large rectangular piece had been cut out of the lid and replaced with fine mesh cloth to allow for ventilation of the fruits. In the laboratory, fruits were removed from transport bags and placed within one-litre, rectangular plastic containers that had small elliptical holes cut out of the bottom. Each one-litre container (also provided with a mesh-covered lid) was nested within a fresh two-litre container, the bottom of which had a layer of heat-treated sand. Fruits were usually held for up to two months. Emerged adult insects were held for 1–3 days before being killed.

Beentje (1994) and Agnew and Agnew (1994) were used for preliminary plant identifications. Identifications made by the second author were confirmed or corrected by Quentin Luke of the East African Herbarium. Plant names and authors correspond to those found in The African Plant Database (2015) and the Missouri Botanical Garden (Tropicos 2015). Voucher specimens of plants are deposited in the collection of the International Centre of Insect Physiology and Ecology (ICIPE) in Nairobi.

Independent of the wild-fruit surveys, at various times between 2005 and 2014, the second author surveyed Kenyan insects using Townes-style Malaise traps (Townes 1972). Traps were run in a total of 76 locations representing diverse habitats, mostly throughout the southern half of the country. Some of the traps were run for a full year. Collecting bottles with 85–90% EtOH were changed every two weeks. Together, these collections represent ca. 458 sampling months (=ca. 13,740 sampling days). Moreover, after completion of the two fruit surveys, occasional collections of wild fruits were made during field trips to collect Malaise trap samples.

### Morphological study

Adult morphology was examined using a MZ12 Leica stereomicroscope equipped with a Leica IC A camera. When more than three specimens were obtained per species and sex, one individual of each sex was dissected and wings of both sexes and male genitalia were mounted on glass slides using Dimethyl hydantoin formaldehyde (DMHF; Mendel 1982). Length of body, head, thorax, gaster and ovipositor sheaths was measured for each specimen using Leica QWIN V2.3® image processing and analysis software. Measurements of forewing stigma characteristics (stigma length, stigma maximal width, length of uncus, upper part of stigmal vein), marginal and post-marginal veins, and of antennal parts were made as described above. To increase depth of field, photographs of entire specimens and body parts were made by combining images taken at multiple focal planes using a Leica Z16 APO A microscope and JVC digital camera KY-F75U. Microvision Cartograph® software was used to combine pictures.

In a few specimens, head, antenna and thoracic sculpture were examined using a Cambridge StereoscanB 90 scanning electron microscope equipped with OrionB® image processing software. When less than three specimens were available per species and sex, these specimens were kept intact and mounted on triangular cardboards. Photographs and measurements of body and body parts (wings, antenna, head, thorax, gaster and ovipositor) were made as above without dissecting the mounted specimens. Morphological terminology follows Roques and Skrzypczyńska (2003). The following abbreviations are used in the text: F1, F2, … Fn: Segment number of antennal funicle; OOL: Ocellocular line: the minimum distance between the eye margin and the adjacent posterior ocellus; POL: Posterior ocellar line: the minimum distance between the posterior ocelli; T3: first apparent tergite of gaster; T4: second apparent tergite; T5, T6, T7, T8, T9: subsequent gaster tergites.

### DNA sequence analysis

• DNA extraction, molecular markers, amplification and sequencing

In order to include the Kenyan species in an existing phylogeny of *Megastigmus*, we used mtDNA sequences previously obtained by Boivin et al. (2014) for 25 species of seed chalcids associated with different host families (Pinaceae, Cupressaceae, Rosaceae, Anacardiaceae; GenBank accession numbers KF531833 to KF531858. Total genomic DNA was isolated by crushing, individually, whole adult females. A segment of the cytochrome oxidase I (COI) gene was amplified by PCR using the primers “Clyde and Bonnie” following Boivin et al. (2014). Whenever possible, the amplification was tested for one to five individuals per species, but for several species, only one specimen had amplifiable DNA although other specimens of the same species were collected. When these two primers did not allow sequencing, we used Bonnie and another primer C1-J-2183 (“Jerry”, 5’-CAACATTTATTTTGATTTTTTGG-3’; Simon et al. 1994), which supplied a shorter dataset but on a larger number of species. PCR products were purified using Nucleospin gel and PCR clean-up kit (Macherey-Nagel, Düren, Germany). Sequencing was performed using the big-dye terminator sequencing kit (PE Applied Biosystems) and carried out with an ABI 3500 Genetic Analyzer (Applied Biosystems, Foster City, California, USA). Specimens were sequenced in both directions.

In addition to the COI gene, a nuclear fragment, the D2 region of the 28S ribosomal subunit (rDNA), was used to build a phylogenetic tree of the studied *Megastigmus* species. Nuclear primers, D1F (5’-ACCCGCTGAATTTAAGCATAT-3’) and D3R (5’-TAGTTCACCATCTTTCGGGTC-3’), previously used for reconstructing a molecular phylogeny of *Megastigmus* spp. on conifers (Auger-Rozenberg et al. 2006), were chosen due to their utility for molecular identification at the intrageneric level. Some sequences were previously obtained by Auger-Rozenberg et al (2006) for 13 species of seed chalcids associated with conifer host families (AY900454, AY90048, AY900460, AY900463, AY900470 to AY900472, AY900474, AY900479, AY900481, AY900486, AY900487, AY900490), and the others were obtained for this study. The species studied molecularly, their collection localities and host plants are summarized in Suppl. material 1

• Phylogenetic analyses

For all datasets, sequences were aligned using Clustal W (Thompson et al. 1994) as implemented in BioEdit 7.05 (Hall 1999). COI was aligned unambiguously and all sequences were truncated to the same length (810 bp for the longest fragment and 417 bp for the shortest). All sequences were translated into amino acids using MEGA 6 (Tamura et al, 2013) to check that no stop codons occurred (Tamura et al. 2013). Genetic distances were calculated on the two COI datasets with MEGA 6 using Kimura-2-parameter (K2P) distance model. Interspecific sequence divergences based on K2P distances were calculated for all species, and mean interspecific K2P divergences were calculated from the pairwise comparisons within and between the clades identified according to host families. For 28S sequences, final alignment was obtained manually and gaps were treated as missing data. The alignment was 924 pb long including gaps.

Phylogenetic analyses were performed using maximum likelihood (ML) inference with PhyML v3.0 (Guindon et al. 2010). The appropriate model of evolution was evaluated with jModeltest v2.1.4 (Darriba et al. 2012). The models selected were GTR+I+G for the different genes. The robustness of the nodes was assessed with 500 bootstrap replicates. Additionally, Bayesian inferences (BI) were also used to reconstruct phylogenies with MrBayes v3.1.2 (Ronquist and Huelsenbeck 2003) using settings leading to convergence between two independent runs (200000 MCMC generations and sampling every 100 generations). Finally, trees were edited with Figtree v1.4.0 (A. Rambaut, http://tree.bio.ed.ac.uk/software/figtree).

### Specimen depositories

Abbreviations used for insect specimen depositories or private collections cited in the study are as follows:

NMKE
 National Museums of Kenya 


RSC
 Robert Copeland personal collection, ICIPE, Nairobi, Kenya 


ARC
 Alain Roques personal collection, INRA Zoologie Forestière Orléans, France 


ICIPE
 International Centre of Insect Physiology and Ecology, Nairobi, Kenya 


SAMC
 IZIKO South African Museum, Capetown, South Africa 


BNHM
 British National History Museum, London, UK 


## Results and discussion

### Megastigmus diversity in Kenya

**Figure 1.**
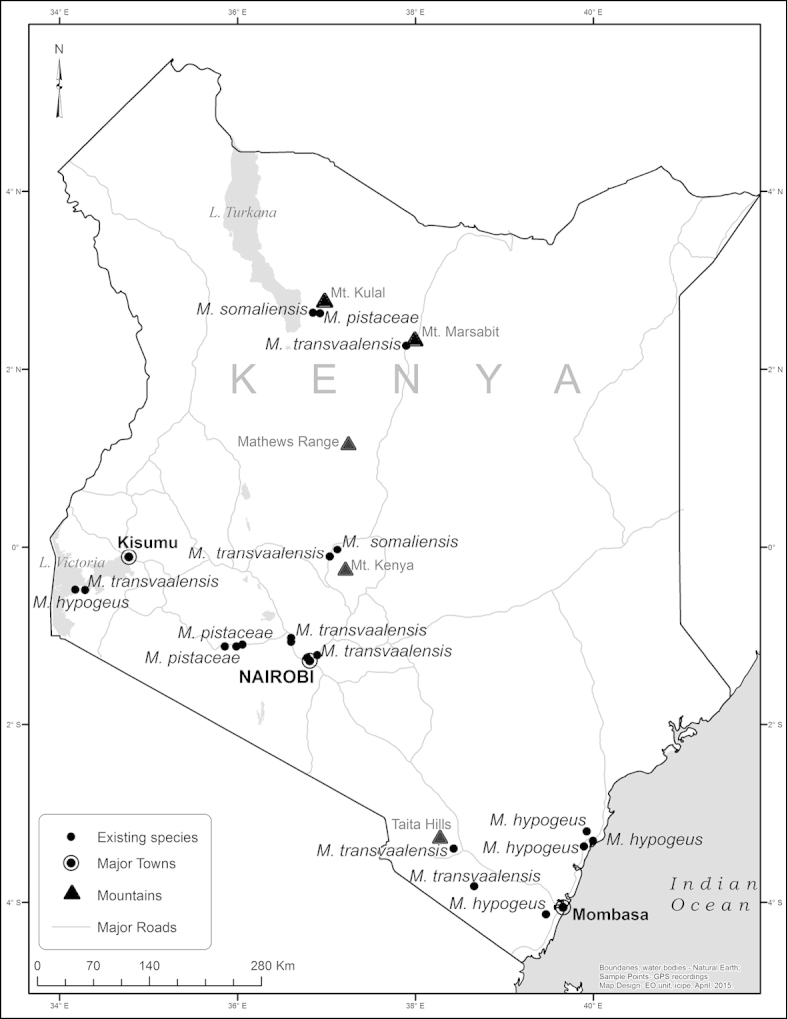
Distribution in Kenya of the previously described African *Megastigmus* species.

**Figure 2.**
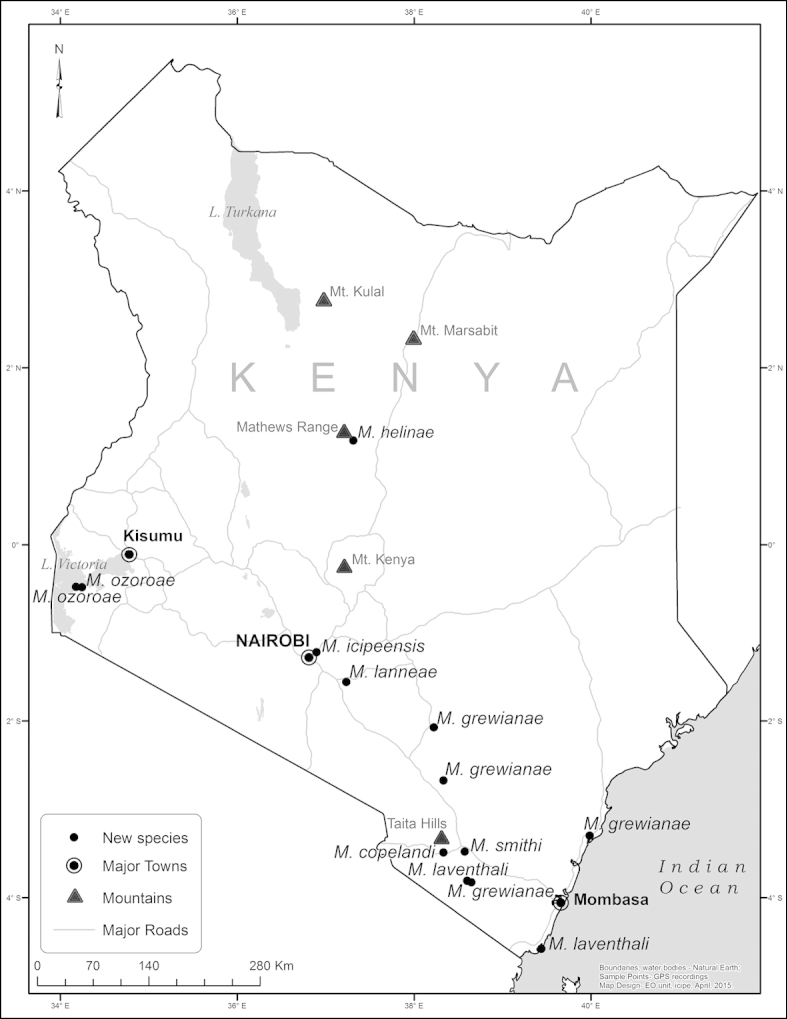
Distribution in Kenya of the new *Megastigmus* species identified during our study.

*Megastigmus* wasps were obtained from 31 of the fruit samples (0.74%- Table 1). Additionally, 10 *Megastigmus* specimens were recovered from four Malaise trap samples. The distribution in Kenya of previously described *Megastigmus* species that we collected from fruit samples and Malaise traps is shown in Figure 1, while sampling locations that yielded new *Megastigmus* species are indicated in Figure 2. Specimens were obtained across the length and breadth of the country, from the Indian Ocean to Lake Victoria in the west and from near Tanzania in the south to Mt. Kulal and Marsabit in the arid north (Figures 1 and 2). They were collected at elevations between near sea-level (Funzi Island) and 2200 m (Kikuyu Escarpment).

**Table 1.** Fruit and Malaise trap samples producing *Megastigmus* species in Kenya.

Family / Host plant species	*Megastigmus* species	Region	Location	Latitude N	Longitude E	Elevation^1^ (m)
**Anacardiaceae**						
*Lannea* cf. *schimperi* (A.Rich.) Engl.	*Megastigmus smithi* Roques & Copeland, sp. n.	Eastern Arc Mountains	Sagalla Mountain	-3,4786	38,5746	1090
*Lannea rivae* (Chiov.) Sacl.	*Megastigmus lanneae* Roques & Copeland, sp. n.	Central highlands	Machakos	-1,5609	37,2338	1586
*Lannea schweinfurthii* (Engl.) Engl.	*Megastigmus laventhali* Roques & Copeland, sp. n.	Eastern Arc Mountains	Base of Kasigau Mountain	-3,8130	38,6405	619
*Ozoroa insignis* Del. subsp. *reticulata* (Bak.f.) Gillett	*Megastigmus ozoroae* Roques & Copeland, sp. n.	Western mid-altitude	Gembe Hills	-0,4894	34,2433	1362
*Ozoroa insignis* subsp. *reticulata*	*Megastigmus ozoroae*	Western mid-altitude	Sindo-Mbita Road	-0,4849	34,1765	1205
*Ozoroa insignis* subsp. *reticulata*	*Megastigmus hypogeus* (Hussey)	Western mid-altitude	Sindo-Mbita Road	-0,4849	34,1765	1205
*Ozoroa obovata* (Oliv.) R. & A. Fernandes	*Megastigmus hypogeus*	Coastal forests	Arabuko-Sokoke Forest	-3,3103	39,9978	55
*Ozoroa obovata*	*Megastigmus hypogeus*	Coastal forests	Arabuko-Sokoke Forest	-3,3716	39,8949	55
*Ozoroa obovata*	*Megastigmus hypogeus*	Coastal forests	Arabuko-Sokoke Forest	-3,2030	39,9271	98
*Ozoroa obovata*	*Megastigmus hypogeus*	Coastal forests	Shimba Hills	-4,1349	39,4680	285
*Pistacia lentiscus* L. subsp. *emarginata* (Engl.) AL-Saghir	*Megastigmus pistaciae* Walker	Western highlands	nr. Narok on main road	-1,1023	36,0192	1919
*Pistacia lentiscus* subsp. *emarginata*	*Megastigmus pistaciae*	Western highlands	nr. Narok on main road	-1,1243	35,9868	1830
*Pistacia lentiscus* subsp. *emarginata*	*Megastigmus pistaciae*	Northern highlands	Mt Kulal	2,6290	36,9278	1639
*Pistacia lentiscus* subsp. *emarginata*	*Megastigmus pistaciae*	Western highlands	Mai Mahiu-Narok Road	-1,1121	35,9765	1900
*Rhus natalensis* Krauss	*Megastigmus transvaalensis* (Hussey)	Central Highlands	Kikuyu Escarpment	-1,0290	36,6025	2100
*Rhus natalensis*	*Megastigmus transvaalensis*	Central Highlands	Kikuyu Escarpment	-1,0717	36,6033	2100
*Rhus natalensis*	*Megastigmus transvaalensis*	Central Highlands	Burguret	-0,1128	37,0375	1960
*Rhus natalensis*	*Megastigmus transvaalensis*	Northern mid-altitude	south of Marsabi town	2,2659	37,8976	1200
*Rhus natalensis*	*Megastigmus transvaalensis*	Western mid-altitude	Gembe Hills	-0,4894	34,2433	1370
*Rhus natalensis*	*Megastigmus transvaalensis*	Eastern Arc Mountains	Ronge-Nyika, mixed shrub- and grassland	-3,3963	38,4309	1050
*Rhus natalensis*	*Megastigmus transvaalensis*	Eastern Arc Mountains	Kasigau forest	-3,8194	38,6619	1280
*Rhus vulgaris* Meikle	*Megastigmus transvaalensis*	Central Highlands	Kikuyu escarpment	-1,0780	36,6020	2200
*Schinus molle* L.	*Megastigmus transvaalensis*	Central Highlands	Nairobi	-1,2211	36,8963	1610
*Schinus terebinthifolius* Raddi	*Megastigmus transvaalensis*	Central highlands	Nairobi	-1,2212	36,8963	1610
*Schinus terebinthifolius*	*Megastigmus transvaalensis*	Central highlands	Nairobi	-1,2501	36,7835	1750
**Cupressaceae**						
*Juniperus procera* Endl.	*Megastigmus somaliensis* (Hussey)	Central highlands	Mt. Kenya Forest	-0,0308	37,1230	2130
*Juniperus procera*	*Megastigmus somaliensis*	Northern highlands	Mt. Kulal	2,6319	36,9282	1650
**Rhamnaceae**						
*Helinus integrifolius* (Lam.) Kuntze	*Megastigmus helinae* Roques & Copeland, sp. n.	Northern highlands	Mathews Range	1,1777	37,3141	1340
**Malvaceae**						
*Grewia stuhlmannii* K. Schum.	*Megastigmus grewianae* Roques & Copeland, sp. n.	Coastal forests	Arabuko-Sokoke Forest	-3,2997	39,9869	60
*Grewia tephrodermis* K. Schum.	*Megastigmus grewianae*	Eastern savanna	Tsavo	-2,6760	38,3325	640
*Grewia tephrodermis*	*Megastigmus copelandi* Roques & Copeland, sp. n.	Eastern Arc Mountains	Mwatate area, on small farm	-3,4894	38,3330	1050
**n.a., Malaise trap**	*Megastigmus grewianae*	Eastern savanna	Kasaala	-2,0749	38,2250	740
**n.a., Malaise trap**	*Megastigmus grewianae*	Eastern Arc Mountains	Kasigau Mountain Forest	-3,8270	38,6488	1070
**n.a., Malaise trap**	*Megastigmus icipeensis* Roques & Copeland, sp. n.	Central Highlands	Nairobi, ICIPE	-1,2232	36,8965	1600
**n.a., Malaise trap**	*Megastigmus laventhali*	Coastal island	Funzi Island	-4,5778	39,4413	0

A total of 12 *Megastigmus* species were found during the survey, of which eight were new to science (Table 1). Among the four previously described species that we reared, *Megastigmus hypogeus* was described from specimens collected in Kenya (Hussey 1956a), and before our surveys it was the only *Megastigmus* species known from the country. *Megastigmus transvaalensis* was reared from Anacardiaceae fruits during the early stages of our project in Kenya and these data were reported in Grissell and Prinsloo (2001) and Scheffer and Grissell (2003). Numerous records of this species also exist for other regions of tropical Africa such as South Africa (Iponga et al. 2008) and the La Réunion island (Roques and Skrzypczynska 2003). Our rearing of *Megastigmus pistaciae* represents the first record in the Afrotropical region of this widespread Palaearctic species (Roques and Skrzypczynska 2003). In addition, two samples of *Juniperus procera* produced *Megastigmus somaliensis*, a species previously described from specimens collected in the former British Somaliland (Hussey 1956b).

*Megastigmus* chalcids were reared from fruits of seven genera of shrubs and trees native to tropical Africa (Table 2), belonging to the families Anacardiaceae (*Lannea*, *Ozoroa*, *Pistacia* and *Rhus*; Figures 3–9), Rhamnaceae (*Helinus*; Figure 10), Malvaceae (*Grewia*; Figure 11–12) and Cupressaceae (*Juniperus*; Figure 13) .

**Figures 3–9.**
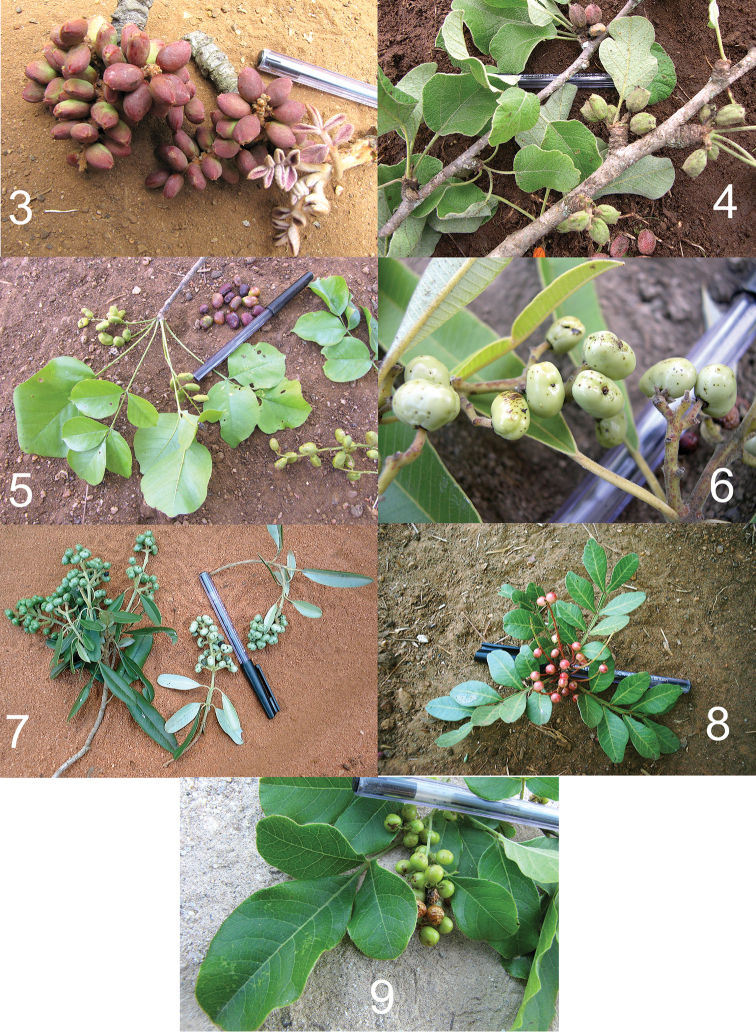
Anacardiaceae host fruits of *Megastigmus* spp. in Kenya. **3** *Lannea* cf. *schimperi* **4** *Lannea rivae* **5** *Lannea schweinfurthii* **6** *Ozoroa insignis* subsp. *reticulata* **7** *Ozoroa obovata* **8** *Pistacia lentiscus* subsp. *emarginata* **9** *Rhus natalensis* (note the chalcid hole in a fruit).

**Figures 10–13.**
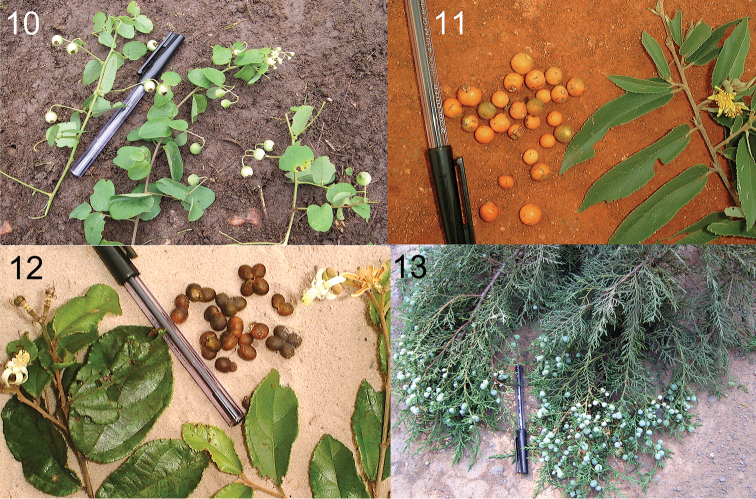
Other host fruits of *Megastigmus* spp. in Kenya. **10** *Helinus integrifolius* (Rhamnaceae) **11** *Grewia tephrodermis* (Malvaceae) **12** *Grewia stuhlmannii* **13** *Juniperus procera* (Cupressaceae).

**Table 2.** Host-plant genera of *Megastigmus* species in Kenya.

Family	Genus	Total no. of Kenyan species	No. of Kenyan species sampled	No. of sampled Kenyan species yielding Megastigmus (%)	Total number of Afrotropical species
Anacardiaceae	*Lannea* ^1^	10	5	3 (60)	29
	*Ozoroa^1^*	2	2	2 (100)	44
	*Pistacia* ^1,2^	1	1	1 (100)	2
	*Rhus* ^1^	6	4	2 (50)	106
	*Schinus* ^1,5^	2^5^	2^5^	2 (100)	2^5^
Cupressaceae	*Juniperus* ^1^	1	1	1 (100)	4
Rhamnaceae ^3^	*Helinus* ^1,2,4^	2	2	1 (50)	3
Malvaceae ^3^	*Grewia* ^1,2,4^	28	14	2 (14)	122

^1^First record of genus as a host of *Megastigmus* in Kenya; ^2^First record of genus as a host of *Megastigmus* in Afrotropical region; ^3^New host-plant family; ^4^New host-plant genus; ^5^ Introduced in Afrotropical region

Of the host families, Anacardiaceae was most important. Including the collections from two exotic *Schinus* species, Anacardiaceae contributed 10 (67%) of the host fruit species (n=15) from which *Megastigmus* were reared. Among these plant hosts, *Lannea* was the most important genus with three of five species sampled producing *Megastigmus*. Each *Lannea* species was attacked by a different, previously undescribed chalcid: *Lannea* cf. *schimperi* (A.Rich.) Engl. (Figure 3) by *Megastigmus smithi* Roques & Copeland, sp. n., *Lannea rivae* (Chiov.) Sacl. (Figure 4) by *Megastigmus lanneae* Roques & Copeland, sp. n., and *Lannea schweinfurthii* (Engl.) Engl. (Figure 5) by *Megastigmus laventhali* Roques & Copeland, sp. n., (Table 1). Both species of Kenyan *Ozoroa* were hosts of *Megastigmus. Ozoroa insignis* Del. subsp. *reticulata* (Bak.f.) Gillett (Figure 6) and *Ozoroa obovata* (Oliv.) R. & A. Fernandes (Figure 7) represent the first confirmed hosts of *Megastigmus hypogeus* (see next paragraph). The former plant is also reported for the first time as a host of *Megastigmus*. *Ozoroa insignis* var. *reticulata* also yielded an undescribed *Megastigmus* species, *Megastigmus ozoroae* Roques & Copeland, sp. n. Other Anacardiaceae hosts were *Pistacia lentiscus* L. subsp. *emarginata* (Engl.) Al-Saghir (Figure 8), a new host record for *Megastigmus pistaciae* Walker, and *Rhus natalensis* Krauss (Figure 9), *Rhus vulgaris* Meikle and the two exotic Anacardiaceae, *Schinus terebinthifolius* Raddi and *Schinus molle* L., all hosts of *Megastigmus transvaalensis*. The two *Rhus* species represent new host records for *Megastigmus transvaalensis*.

Before our study the host of *Megastigmus hypogeus* was an unresolved issue. When transcribing label information in his description of *Megastigmus hypogeus* from specimens then available to him, Hussey (1956a) reported the host as “Oil seed, probably *Helianthus* (Compositae)”. This seemed unlikely as no Asteraceae has been reported as a host of phytophagous *Megastigmus*. We were able to examine Hussey’s holotype and paratypes of *Megastigmus hypogeus* as well as additional material deposited at BNHM and it appeared that our Kenyan specimens undoubtedly belong to the same species. Thus, our study appears to have resolved this host question as we reared *Megastigmus hypogeus* from several collections of *Ozoroa insignis* subsp. *reticulata* and *Ozoroa obovota*. Another *Megastigmus* species, *Megastigmus thomseni* (Hussey), is known to develop in seeds of *Ozoroa obovota* as well as in those of *Ozoroa paniculosa* (Sond.) and *Lannea discolor* (Sond.) Engl. in South Africa (Grissell and Prinsloo 2001). The examination of Hussey’s type material kept at BNHM confirmed that *Megastigmus thomseni* was not present in the chalcid specimens collected during our survey.

Among non-Anacardiaceae host families, two new *Megastigmus* species were obtained from fruits of two species of Malvaceae in the genus *Grewia. Megastigmus grewianae* Roques & Copeland, sp. n. emerged from both *Grewia tephrodermis* K. Schum. (Figure 11) and *Grewia stuhlmannii* K. Schum. (Figure 12), while *Megastigmus copelandi* Roques & Copeland, sp. n. was reared from *Grewia tephrodermis*. *Megastigmus helinae* Roques & Copeland, sp. n. was reared from seeds of the Rhamnaceae *Helinus integrifolius* (Lam.) Kuntze (Figure 10), collected in the northern Kenya mountains of the Mathews Range. These are the first records of *Megastigmus* seed chalcids from Malvaceae and Rhamnaceae. Finally, we reared *Megastigmus somaliensis* from two collections of *Juniperus procera* (Figure 13). The examination of Hussey’s type material kept at BNHM allowed us to confirm the species identity. The remaining species, *Megastigmus icipeensis* Roques & Copeland, sp. n., has no known host, having been collected only in Malaise trap samples.

Table 3 presents the infestation indices of the host fruits. The percentage of fruits infested by chalcids ranged from 0.1 to 14.8% depending on both the *Megastigmus* species and the host plant. However, the infestation only exceeded 10% of the fruits in two species of Anacardiaceae, *Rhus vulgaris* and the introduced pinkpepper tree *Schinus molle*, both attacked by *Megastigmus transvaalensis*, and in the Malvaceae *Grewia tephrodermis*, attacked by *Megastigmus grewianae*.

**Table 3.** Host fruit infestation indices for *Megastigmus* species. Only samples whose fruits were counted are included; no samples of *Juniperus procera* fruits were counted.

Plant family/ Plant species	No. of fruits	Megastigmus species	No. of reared *Megastigmus*	% of infestation by *Megastigmus*
**Anacardiaceae**				
*Lannea* cf. *schimperi*	786	*Megastigmus smithi*	3	0,4
*Lannea rivae*	338	*Megastigmus lanneae*	11	3,3
*Lannea schweinfurthii*	168	*Megastigmus laventhali*	1	0,6
*Ozoroa insignis* subsp. *reticulata*	2806	*Megastigmus ozoroae*	226	8,1
*Ozoroa obovata*	1505	*Megastigmus hypogeus*	42	2,8
*Ozoroa obovata*	698	*Megastigmus hypogeus*	1	0,1
*Ozoroa obovata*	2442	*Megastigmus hypogeus*	22	0,9
*Pistacia lentiscus* subsp. *emarginata*	2603	*Megastigmus pistaciae*	12	0,5
*Pistacia lentiscus* subsp. *emarginata*	1019	*Megastigmus pistaciae*	1	0,1
*Rhus natalensis*	674	*Megastigmus transvaalensis*	41	6,1
*Rhus natalensis*	524	*Megastigmus transvaalensis*	6	1,1
*Rhus natalensis*	2394	*Megastigmus transvaalensis*	14	0,6
*Rhus natalensis*	894	*Megastigmus transvaalensis*	36	4,0
*Rhus natalensis*	412	*Megastigmus transvaalensis*	6	1,5
*Rhus vulgaris*	769	*Megastigmus transvaalensis*	113	14,7
*Schinus molle*	535	*Megastigmus transvaalensis*	79	14,8
**Rhamnaceae**				
*Helinus integrifolius*	82	*Megastigmus helinae*	8	9,8
**Malvaceae**				
*Grewia tephrodermis*	448	*Megastigmus copelandi*	3	0,7
*Grewia tephrodermis*	100	*Megastigmus grewianae*	13	13,0
*Grewia stuhlmannii*	1056	*Megastigmus grewianae*	29	2,7

The four Malaise trap samples containing *Megastigmus* yielded a total of 10 specimens. Given our estimate of 13,740 Malaise trap sampling days, *Megastigmus* were captured at the very low rate of approximately one specimen per 1374 sampling days. Despite this, a total of three species of *Megastigmus* were collected, all of them representing previously undescribed species. Single specimens of *Megastigmus grewianae* Roques & Copeland, sp. n. were collected in Malaise traps set in dry savanna in Eastern Kenya (Kasaala) and in a small campsite clearing in Kasigau Forest (Table 1, Figure 2). In addition to being reared from *Lannea schweinfurthii*, a single specimen of *Megastigmus laventhali* Roques & Copeland, sp. n. was captured in a Malaise trap set on Funzi Island, just off the coast of Kenya. Finally, a Malaise trap set alongside a small stream in a sprawling semi-urban part of Nairobi yielded five female specimens of *Megastigmus icipeensis* Roques & Copeland, sp. n. and three males, which probably belong to the same species. In contrast with other groups of Microhymenoptera (e.g. Dryinidae; Olmi et al. 2015) Malaise traps produced very few specimens of *Megastigmus*. Although each of the four Malaise samples with *Megastigmus* contained an undescribed species, only one of the species (*Megastigmus icipeensis*), was not represented in the fruit samples. Fruit collection is presently the best hope for discovering new host plant taxa and new *Megastigmus* species.

Our fruit collections bring to six the number of Afrotropical plant families that are hosts of *Megastigmus*, including Rosaceae which are attacked by the introduced species, *Megastigmus aculeatus*. The listing, in a summary table, by Grissell (1999) of the presence in the Afrotropical region of *Megastigmus rosae* Bouček, another palearctic species associated with seeds of wild roses, was probably in error as it is not mentioned in the species accounts (Grissell 1999). Moreover, no such record is found in Noyes (2015).

Table 2 summarizes data on the number of species of known host-plant genera found in Kenya and in the Afrotropical region. The Anacardiaceae genera *Rhus* and, to a lesser extent, *Ozoroa* and *Lannea* are speciose, as is the Malvaceae genus *Grewia*. Considering that the great majority of species of these plant genera remain to be sampled it is reasonable to expect that targeted fruit collections will yield considerably more species of Afrotropical *Megastigmus*. Without exception, fruits used by *Megastigmus* species were small and relatively dry, with little pulp surrounding the seed (Figures 3–13). Of plant families known to be hosts, species with larger and wetter fruits were never found to harbor *Megastigmus*. For example, among the Anacardiaceae with large, fleshy fruits that we sampled, the genera *Anacardium* (1 sample), *Harpephyllum* (6 samples), *Mangifera* (3 samples), *Pseudospondias* (2 samples), *Sclerocarya* (15 samples), and *Sorindeia* (10 samples) all failed to produce *Megastigmus* (Copeland et al. 2009).

### Molecular confirmation of specific identity of the *Megastigmus* species and phylogeny

MtDNA was sequenced for nine of the 12 African species defined morphologically, including five of the eight new species and the four previously described ones. Using the longest mitochondrial marker (“Clyde-Bonnie”), 810 bp- long COI sequences were obtained for *Megastigmus helinae* (1 specimen), *Megastigmus grewianae* (4 specimens), *Megastigmus lanneae* (1 specimen), *Megastigmus pistaciae* (2 specimens), *Megastigmus somaliensis* (1 specimen) and *Megastigmus zebrinus* (1 specimen). In the case of *Megastigmus hypogeus* and *Megastigmus ozoroae*, six specimens per species were extracted but we obtained only a short fragment of 417 bp with the internal primer “Jerry” for one individual of *Megastigmus ozoroae*, and for two of *Megastigmus hypogeus*. Similarly, we obtained this short fragment for the sole specimen of *Megastigmus icipeensis*. DNA from the other species was amplified with the different sets of primers and, although we obtained data from more species with the shorter fragment than with the longer, we studied and compared the two datasets because some short sequences showed the presence of overlapping fragments (for less than 2% of the short sequence) which could be due to heteroplasmy or numt’s (nuclear copies of mitochondrial DNA), even if they were of the correct length and reading frame and contained no stop codons. These trends were observed in *Megastigmus lanneae* and *Megastigmus ozoroae* and their occurrence seemed to be species specific. These problems (no amplification and/or overlapping fragments) could be due to the preservation quality of the specimens. For *Megastigmus transvaalensis*, we used sequences from specimens previously obtained from Morocco, because we failed to amplify any Kenyan specimens. *Megastigmus copelandi*, *Megastigmus laventhali* and *Megastigmus smithi* were not analyzed molecularly because of the limited number of available specimens necessary to define holotype and paratypes. For the nuclear marker 28S, one specimen per species was successfully amplified for all the species analyzed in the COI dataset except *Megastigmus icipeensis*, and an alignment of 924 bp including gaps was obtained.

The inclusion of the nine Kenyan species in an already existing molecular phylogeny (Boivin et al. 2014) and their resulting phylogenetic position confirmed without ambiguity that none of the Kenyan species was synonymous with previously examined species. After grouping *Megastigmus* species according to their host plant families (Pinaceae / Cupressaceae / Taxodiaceae / Rosaceae / Anacardiaceae / Malvaceae / Rhamnaceae), we calculated within- and between-group mean distances for the two COI datasets (Table 4). In both cases lower values were observed within groups than between groups, with values ranged from 6% to 7.9% for COI-long fragment and from 4.6% to 6.8% for COI-short fragment (with no values for Malvaceae, Rhamnaceae or gall-former because there was only one species per group). Values within the Anacardiaceae group were quite similar for both datasets (6.8% and 6.3%), and are consistent with the values observed within the Holarctic groups (Pinaceae / Cupressaceae / Taxodiaceae / Rosaceae).

**Table 4.** Mean genetic distances within and between groups of *Megastigmus* sequences. For each cell: COI long fragment before backslash (/), and COI short fragment after backslash (in this case, the group “unknown” is corresponding to the only specimen of *Megastigmus icipeensis*, caught in malaise trap and only sequenced in short fragment).

	Pinaceae	Cupressaceae	Taxodiaceae	Rosaceae	Tiliaceae	Rhamnaceae	Anacardiaceae	Gall-former	unknown	outgroup
Pinaceae	0.061\0.057									
Cupressaceae	0.088\0.073	0.060\0.046								
Taxodiaceae	0.081\0.074	0.084\0.074	-\-							
Rosaceae	0.085\0.074	0.093\0.080	0.086\0.077	0.079\0.068						
Tiliaceae	0.098\0.086	0.089\0.080	0.094\0.083	0.102\0.087	-\-					
Rhamnaceae	0.113\0.095	0.112\0.105	0.107\0.096	0.108\0.088	0.111\0.097	-\-				
Anacardiaceae	0.111\0.096	0.112\0.099	0.099\0.104	0.108\0.100	0.111\0.095	0.107\0.078	0.068\0.063			
Gall-former	0.102\0.092	0.100\0.085	0.094\0.096	0.100\0.086	0.106\0.080	0.094\0.086	0.105\0.092	-\-		
Unknown	-\0.094	-\0.089	-\0.096	-\0.094	-\0.096	-\0.083	-\0.082	-\0.083		
Outgroup	0.157\0.127	0.151\0.123	0.158\0.135	0.157\0.134	0.161\0.132	0.140\0.113	0.146\0.115	0.154\0.121	-\0.113	-\-

Between groups, similar values were observed between Holarctic groups, as well as between the species related to Malvaceae and the Holarctic groups, whereas pairwise K2P distances showed higher values for the other host families. In the COI-long fragment dataset, the values between *Megastigmus somaliensis* and the two other species related to the genus *Juniperus* (Cupressaceae) were 3.5%. The two specimens identified as *Megastigmus pistaciae* (one from Greece and one from Kenya) diverged by 4.5%, suggesting that the two are probably sibling species.

**Figure 14.**
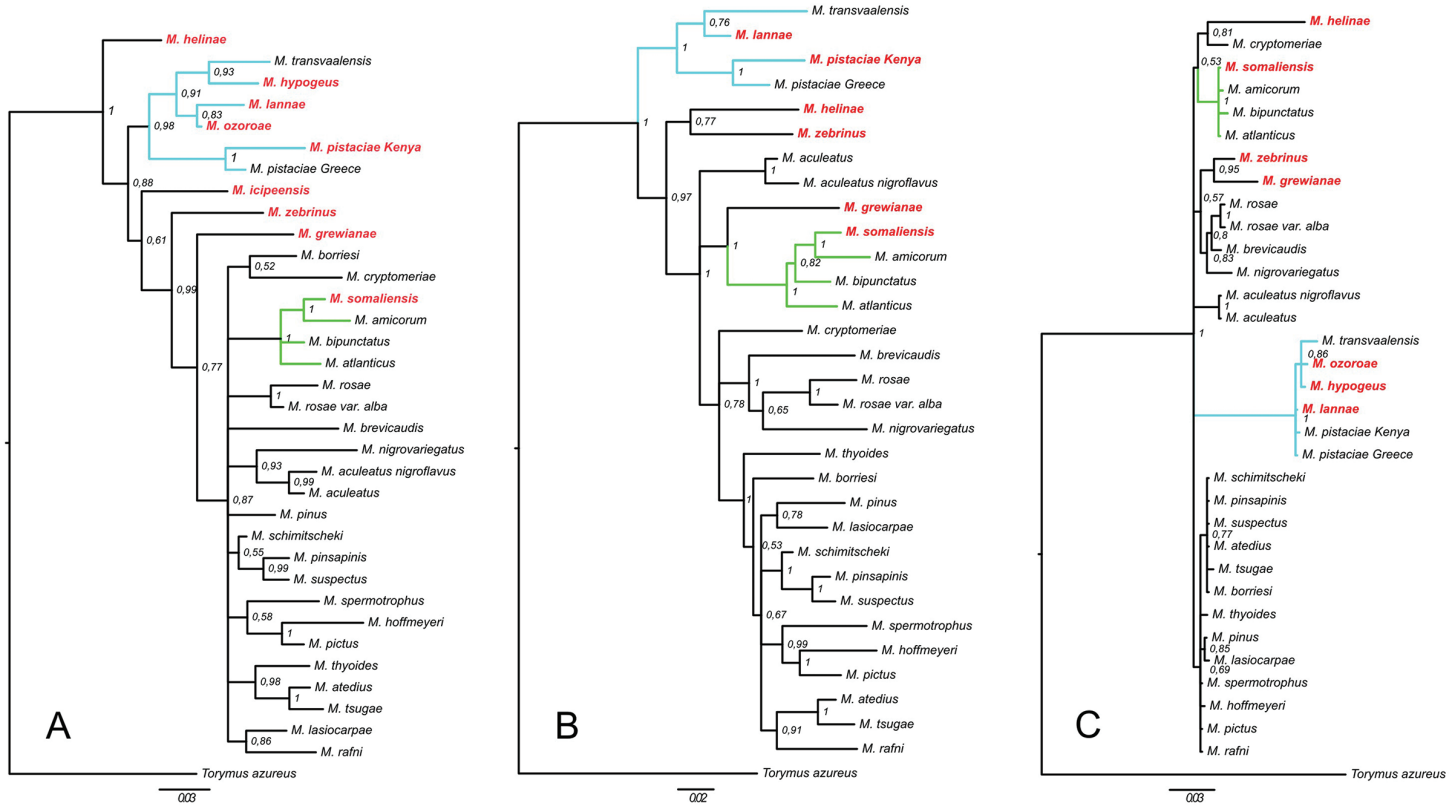
Bayesian-likelihood inference phylogenies based on cytochrome oxidase I (COI) and ribosomal DNA (28S) sequences in seed-specialized wasps of the *Megastigmus* genus. **A** Short fragment COI (34 taxa, 417bp) **B** Long fragment COI (31 taxa, 810pb) **C** Nuclear fragment (33 taxa, 924pb). *Torymus azureus* Boheman, 1834 was used as an outgroup. Posterior probability values are indicated at each node. New taxa described in this paper are indicated in red and bold. Branches of taxa associated with Anacardiaceae as host plant family are in blue, and branches of taxa associated with Cupressaceae are in green.

The use of maximum likelihood and Bayesian inference methods resulted in similar information about phylogenetic relationships within the genus *Megastigmus*. Therefore, we present only the phylogenetic trees built with the Bayesian inference method (Figure 14). The phylogenetic reconstruction was carried out by adding, where possible, one sequence (selected at random when there was more than one specimen) per Kenyan species to the sequences of the 25 *Megastigmus* species considered in Boivin et al. (2014). The current dataset thus included 32 taxa for the COI-long fragment, 35 for the COI-short one and 34 for the 28S marker. To obtain phylogenetic trees including as many taxa as possible, after alignment we compared the sequenced regions common to all taxa for analysis; i.e. the 28S and COI-short fragment. For the mtDNA analysis, the trees obtained with COI-long fragment were considered first because of the greater reliability and safety of long fragments vs short ones. Our phylogenetic reconstruction based on evidence from two markers confirmed previous studies (Auger-Rozenberg et al. 2006; Boivin et al. 2014) that linked host families specialization with genetic similarity. The phylogenetic trees were analysed with a focus on the Kenyan species, and some of the new species are associated with host families already present in the phylogeny (Anacardiaceae and Cupressaceae). In those cases, they always clustered in the corresponding clade regardless of the marker. For the other host families (i.e. associated with Rhamnaceae and Malvaceae or described as gall-former) for which no species were already described, the phylogenetic position remains unclear.

In the different trees, robust clades (Pinaceae, Cupressaceae, Rosaceae, Anacardiaceae) included several species, whereas the positions of single species varies depending of the marker. Whatever the marker and method, the *Megastigmus* species associated with Anacardiaceae clustered together in a highly supported monophyletic clade (Figure 14). Within this clade, the two specimens collected on *Pistacia* always clustered indicating a phylogenetic proximity. The basal position of this clade in the COI dataset and its large branch length in the nuclear dataset indicated an ancestral split between the species associated with Anacardiaceae and those reared from other plant families. However, the phylogenetic position of *Megastigmus helinae* (Rhamnaceae), *Megastigmus grewianae* (Malvaceae) and *Megastigmus zebrinus* (gall-former) was unstable and unresolved. Similarly, the position of *Megastigmus icipeensis* (caught in malaise trap) remained unclear. For this last species, as for *Megastigmus hypogeus* and *Megastigmus ozoroae* (both associated with Anacardiaceae), mtDNA amplification produced only the shortest COI fragment. The study of this gene fragment was very useful because some species were only available in this dataset, although it is recognized that longer fragments are usually more reliable in identification of insects (Aly 2014) and that the presence of numts can skew the phylogenetic relationships, leading to mtDNA divergence that is often lineage specific (Song et al. 2008). Details for each species studied both morphologically and molecularly are provided in the key below.

Despite amplifying for all markers used, the phylogenetic position of *Megastigmus zebrinus* remains uncertain. According to Grissell (2006), *Megastigmus zebrinus*, a gall-forming species introduced with its *Eucalyptus* host, has modified its feeding behaviour by adapting to the seeds of a new host, *Syzygium cordatum*, endemic to the Afrotropical region. In our phylogenetic trees (in addition to the genetic distance calculated from the COI fragments) it clusters with the strictly seminiphagous species, and this brings it close to the Kenyan species. It would be very useful to confirm its degree of molecular similarity with Australian gall-formers in order to disentangle the evolutionary strategies that allow a species to shift to new host-plant taxa and adapt to new host-plant reproductive structures.

The mtDNA sequences and nuclear sequences determined in the course of this study are registered under the following GenBank accession numbers: KU984677 to KU984706.

### Key to the species of *Megastigmus* of Eastern and Southern Africa

In some *Megastigmus* species, males, unlike females, are highly variable in size and colour. This is observed especially in species associated with Anacardiaceae (e.g., *Megastigmus transvaalensis*; Grissell and Prinsloo 2001; *Megastigmus pistaciae*, Roques and Skrzypczyńska 2003) and Cupressaceae (*Megastigmus amicorum* Bouček, *Megastigmus wachtli* Seitner; Roques and Skrzypczyńska 2003), but also in species associated with galls such as *Megastigmus zebrinus* (Grissell, 2006). In *Megastigmus transvaalensis*, Grissell and Prinsloo (2001) observed two extreme morphs, small yellow specimens vs. large ones predominantly colored in black and with a large, infuscate stigma, and pointed out that these forms are bridged by intermediates along a gradual cline. Many pale and dark male morphs were seen by us in rearings of *Megastigmus lanneae* (Figures 103 and 110), *Megastigmus ozoroae* (Figures 49 and 56) and *Megastigmus hypogeus* (Figures 123 and 130) These males were easily assigned to their respective species because they emerged together with many females of only one species. In the absence of DNA confirmation, when few specimens are reared, variations in size and colour make it difficult to assign males and females to the same species with certainty, particularly when adults emerge at different times or at different sites, or when they were caught in Malaise traps.

Therefore, we provide a key to females only, including the species associated with rose hips, conifers, and galls that were not collected during the surveys (i.e. *Megastigmus aculeatus*, *Megastigmus thomseni*, *Megastigmus pretorianensis* and *Megastigmus zebrinus*). We also include *Megastigmus asir* Ghramh & Shati recorded from seeds of *Juniperus procera* in a region of Saudi Arabia facing Eritrea and Ethiopia (Ghramh and Shati 2011). The key relies strongly on the relative length of the exserted part of the ovipositor which we consider to be a stable character, related to the species’ adaptation to host exploitation (Roques and Skrzypczyńska 2003).

### Key to Megastigmus females of Eastern and Southern Africa

**Table:** 

1	Ovipositor sheaths shorter (0.4–0.9×) than gaster length (Figures 16, 31, 37, 43)	**2**
–	Ovipositor sheaths longer (1.1×–1.8×) than gaster length (Figures 63, 76, 97, 117, 137, 151, 163)	**5**
2	Ovipositor sheaths much shorter (0.4–0.6×) than gaster length (Figures 16, 31)	**3**
-–	Ovipositor sheaths 0.8–0.9× as long as gaster length (Figures 37, 43)	**4**
3	Body colour nearly entirely orange-yellow; only a narrow black band along anterior suture of pronotum (Figures 15, 16)	***Megastigmus helinae* Roques & Copeland, sp. n.**
-	Body colour with a large longitudinal, median rufous band extending from pronotum to scutellum (Figure 30, 32) and parts of gaster dark brown (Figure 30)	***Megastigmus smithi* Roques & Copeland, sp. n.**
4	Pilosity on head entirely pale (Figure 39); pilosity on propodeum, coxa and legs entirely pale; body extensively orange except a few black patterns (Figure 37)	***Megastigmus copelandi* Roques & Copeland, sp. n.**
–	Pilosity pale on face but dark on other parts of head (Figure 45); pilosity on propodeum, coxa and legs dark; body colour yellow and black (Figures 42, 43, 44)	***Megastigmus ozoroae* Roques & Copeland, sp. n.**
5	Minute species, body length less than 1.5 mm; Thorax pilosity pale; 2 pairs of hairs on scutellum (Figure 64)	**6**
–	Larger species, body length more than 2.5 mm; Thorax pilosity dark; from 3 to 8 pairs of hairs on scutellum (Figures 77, 83, 98, 118, 131, 138, 152)	**7**
6	Body colour mostly black	***Megastigmus pretorianensis* Do**ǧ**anlar**
–	Body colour nearly completely orange-yellow (Figure 64)	***Megastigmus icipeensis* Roques & Copeland, sp. n.**
7	Thorax colour orange or pale yellow without extensive dark markings (Figures 77, 138, 152)	**8**
–	Thorax with extensive dark markings (Figures 83, 98, 118, 163)	**12**
8	Ovipositor sheaths less than 1.2× as long as gaster length (Figure 137)	**9**
–	Ovipositor sheaths at least 1.4× as long as gaster length (Figures 76, 151)	**10**
9	Forewing with an elongate stigma, 1.7× as long as wide	***Megastigmus thomseni* (Hussey)**
–	Forewing with an oval- rounded stigma, 1.2–1.3 × as long as wide (Figure 141)	***Megastigmus pistaciae* Walker**
10	Ovipositor sheaths at least 1.8 × as long as gaster length and 0.8× as long as body length (Figure 76); forewing stigma oval, 1.5 × as long as wide (Figure 80)	***Megastigmus laventhali* Roques & Copeland, sp. n.**
–	Ovipositor sheaths at most 1.6× as long as gaster length and 0.6× as long as body length (Figure 151); forewing stigma more elongate, at least 1.6 × as long as wide (Figure 155)	**11**
11	Forewing stigma oblong, 1.7 × as long as wide, with a very short stigmal vein, at most 0.2× as long as stigma length; gaster tergites pale yellow with translucent median longitudinal stripes, transversely infumate	***Megastigmus zebrinus* Grissell**
–	Forewing stigma elongate-oval, 2.0 × as long as wide, with an elongate stigmal vein, 0.4 × as long as stigma length (Figure 155); gaster tergites light orange-brown with transverse dark brown bands	***Megastigmus transvaalensis* (Hussey)**
12	Ovipositor sheaths never longer than 1.5× gaster length (Figures 117, 163)	**13**
–	Ovipositor sheaths at least 1.8× longer than gaster length (Figures 82, 97)	**15**
13	Pronotum with a large median dark brown band progressively decreasing in size from the anterior to posterior suture (Figure 118); scutellum with 3–4 pairs of hairs; in seeds of *Ozoroa*	***Megastigmus hypogeus* (Hussey)**
–	Pronotum yellow or brownish, without median dark band; scutellum with 7–8 pairs of hairs; in seeds of *Juniperus procera*	**14**
14	Propodeum smooth; forewing stigma elongate, more than 2× as long as broad	***Megastigmus asir* Ghramh & Shati**
–	Propodeum rugose, with reticulate striae (Figure 166); forewing stigma oval, 1.5 × as long as broad (Figure 167)	***Megastigmus somaliensis* Hussey**
15	Ovipositor sheaths at least 1.2× longer than body length. In *Rosa* seeds	***Megastigmus aculeatus* Swederus**
–	Ovipositor sheaths never longer than body length. In Malvaceae or Anacardiaceae seed	**16**
16	Pronotum orange with a conspicuous longitudinal black stripe on each side (Figures 81, 82, 83); in Malvaceae seeds	***Megastigmus grewianae* Roques & Copeland, sp. n.**
–	Pronotum without lateral black stripes but with a large, nearly rectangular dark brown band medially extending from anterior to posterior suture (Figures 96, 98); in Anacardiaceae seeds	***Megastigmus lanneae* Roques & Copeland, sp. n.**

**Figures 15–21.**
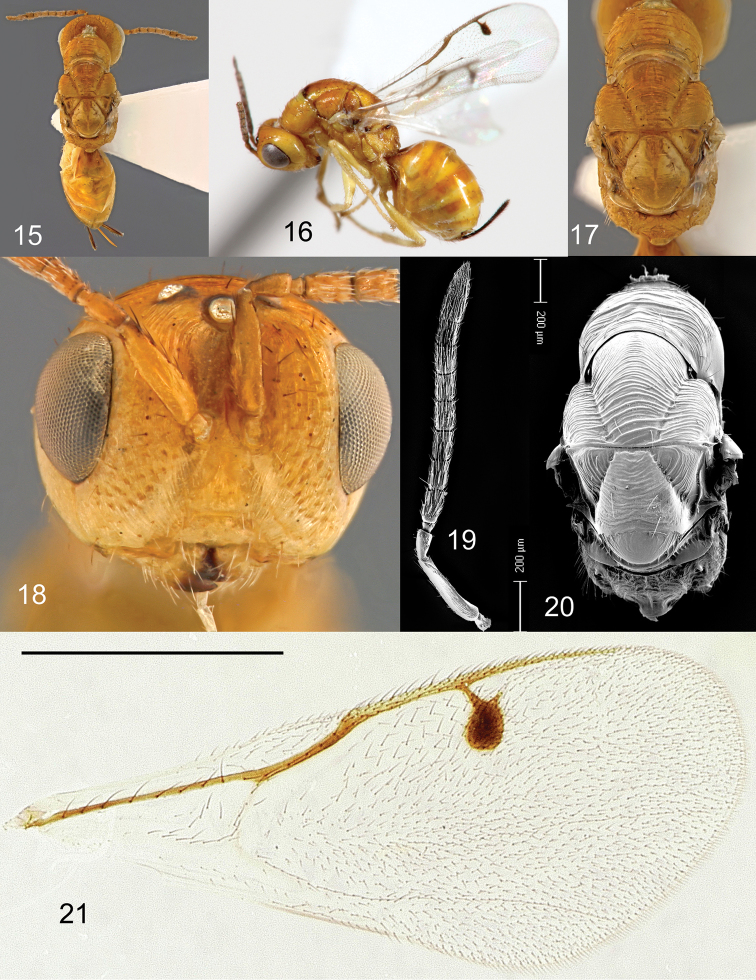
*Megastigmus helinae* Roques & Copeland, sp. n. female. **15** dorsal view of the body **16** lateral view of body **17** dorsal view of thorax **18** front view of head **19** electroscan of antenna **20** electroscan of dorsal view of thorax **21** forewing.

**Figures 22–29.**
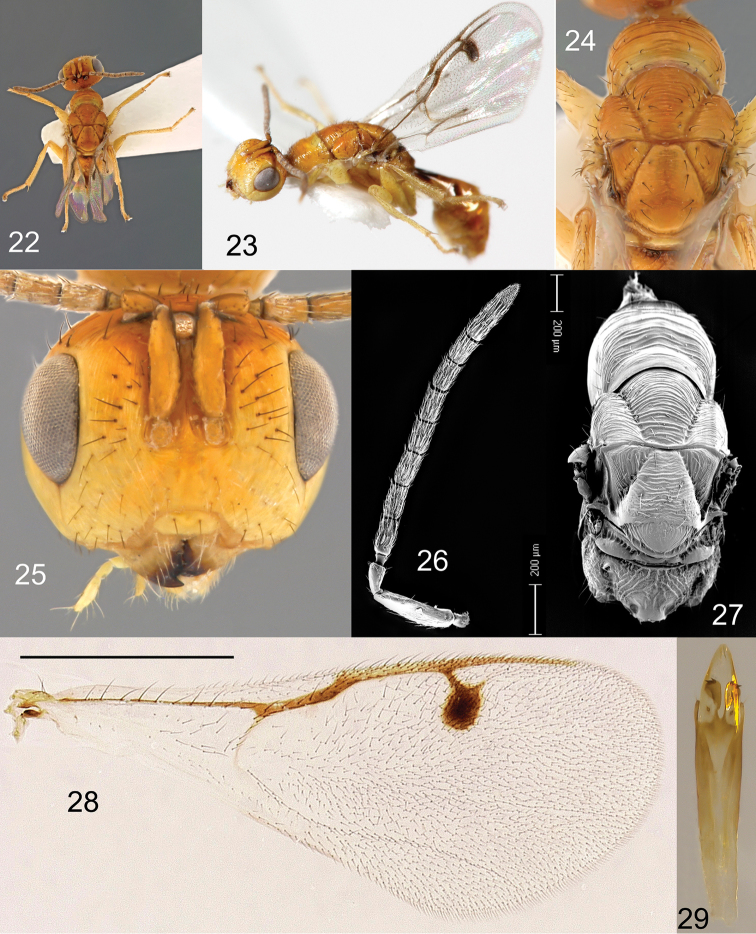
*Megastigmus helinae* Roques & Copeland, sp. n. male. **22** dorsal view of the body **23** lateral view of body **24** dorsal view of thorax **25** front view of head **26** electroscan of antenna **27** electroscan of dorsal view of thorax **28** forewing **29** genitalia.

**Figures 30–35.**
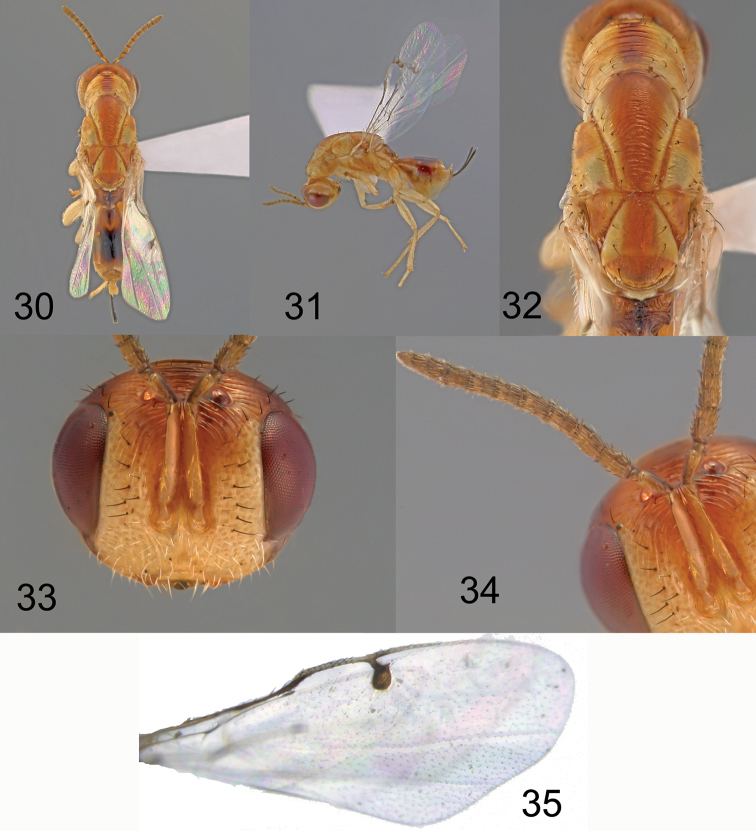
*Megastigmus smithi* Roques & Copeland, sp. n. female. **30** dorsal view of the body **31** lateral view of body **32** dorsal view of thorax **33** front view of head **34** antenna **35** forewing.

**Figures 36–41.**
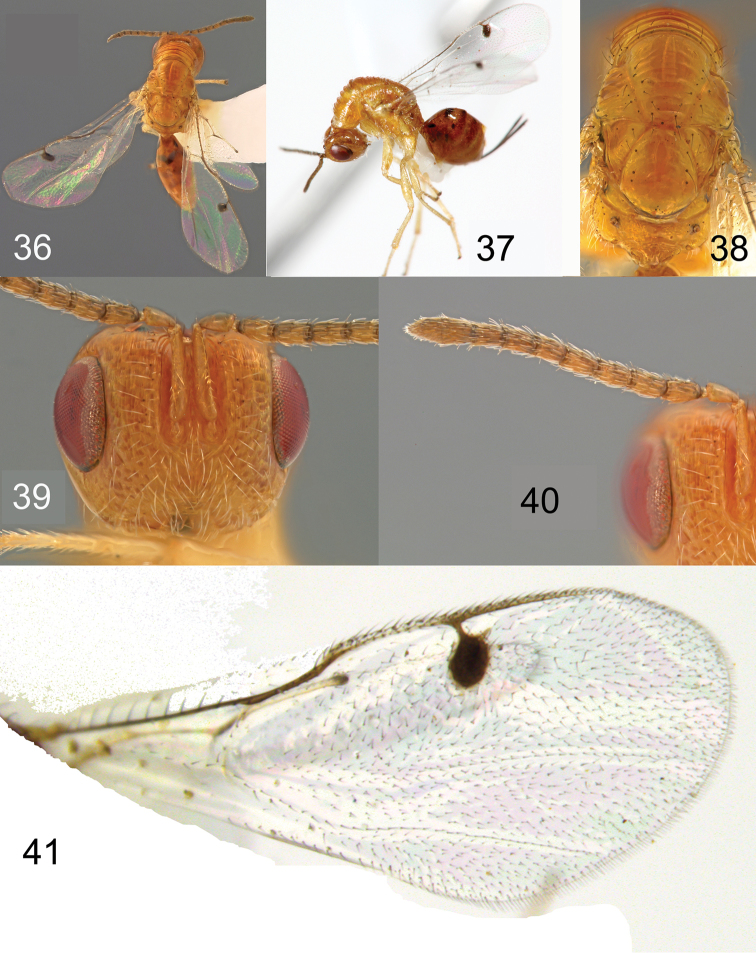
*Megastigmus copelandi* Roques & Copeland, sp. n. female. **36** dorsal view of the body **37** lateral view of body **38** dorsal view of thorax; **39** front view of head **40** antenna **41** forewing.

**Figures 42–47.**
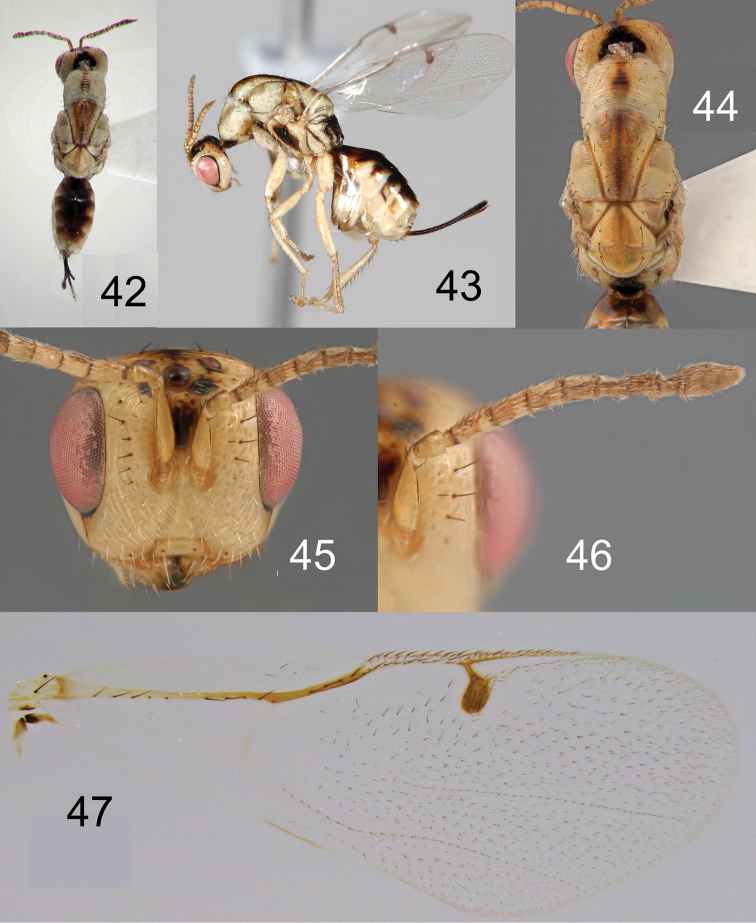
*Megastigmus ozoroae* Roques & Copeland, sp. n. female. **42** dorsal view of the body **43** lateral view of body **44** dorsal view of thorax **45** front view of head **46** antenna **47** forewing.

**Figures 48–54.**
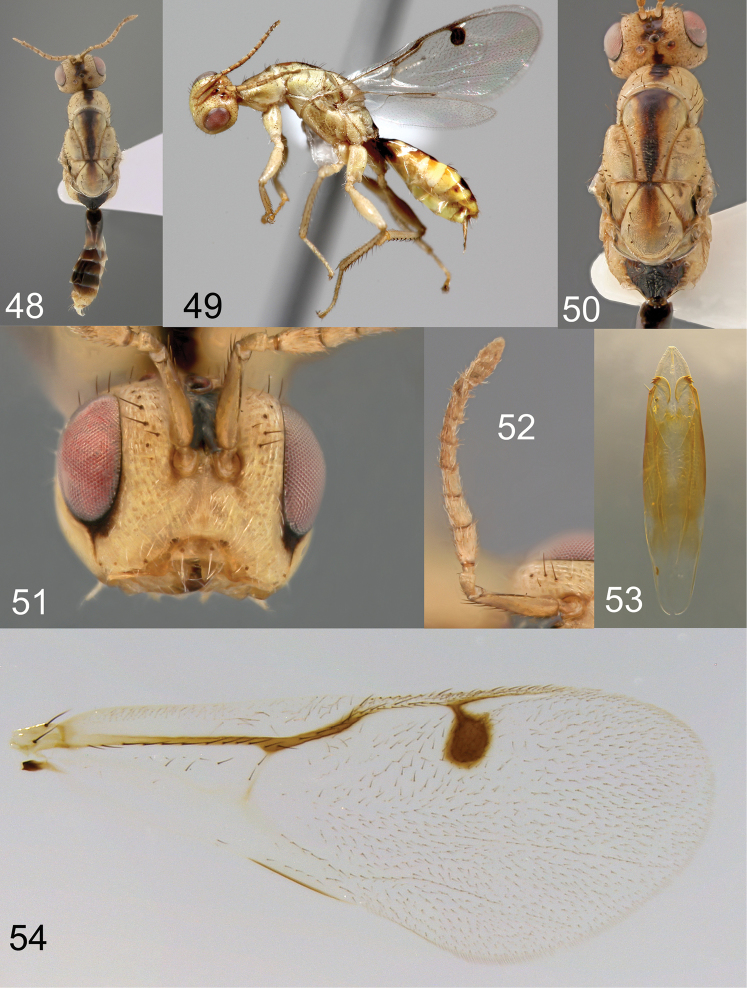
*Megastigmus ozoroae* Roques & Copeland, sp. n. male pale form. **48** dorsal view of the body **49** lateral view of body **50** dorsal view of thorax; **51** front view of head **52** antenna **53** genitalia **54** forewing.

**Figures 55–61.**
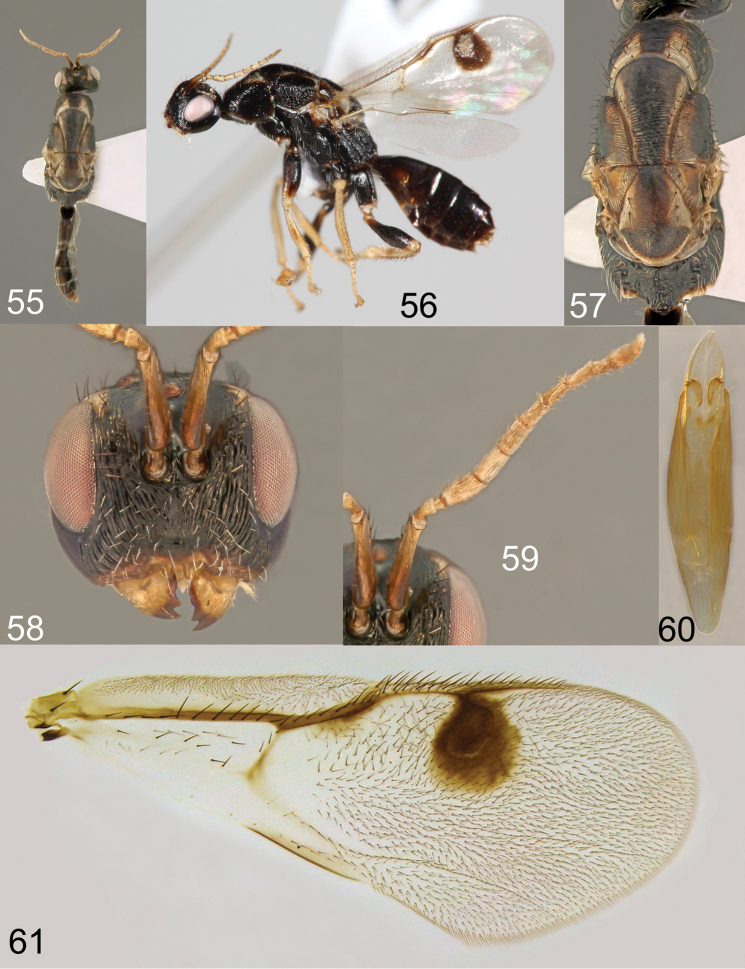
*Megastigmus ozoroae* Roques & Copeland, sp. n. male dark form. **55** dorsal view of the body **56** lateral view of body **57** dorsal view of thorax; **58** front view of head **59** antenna **60** genitalia **61** forewing.

**Figures 62–67.**
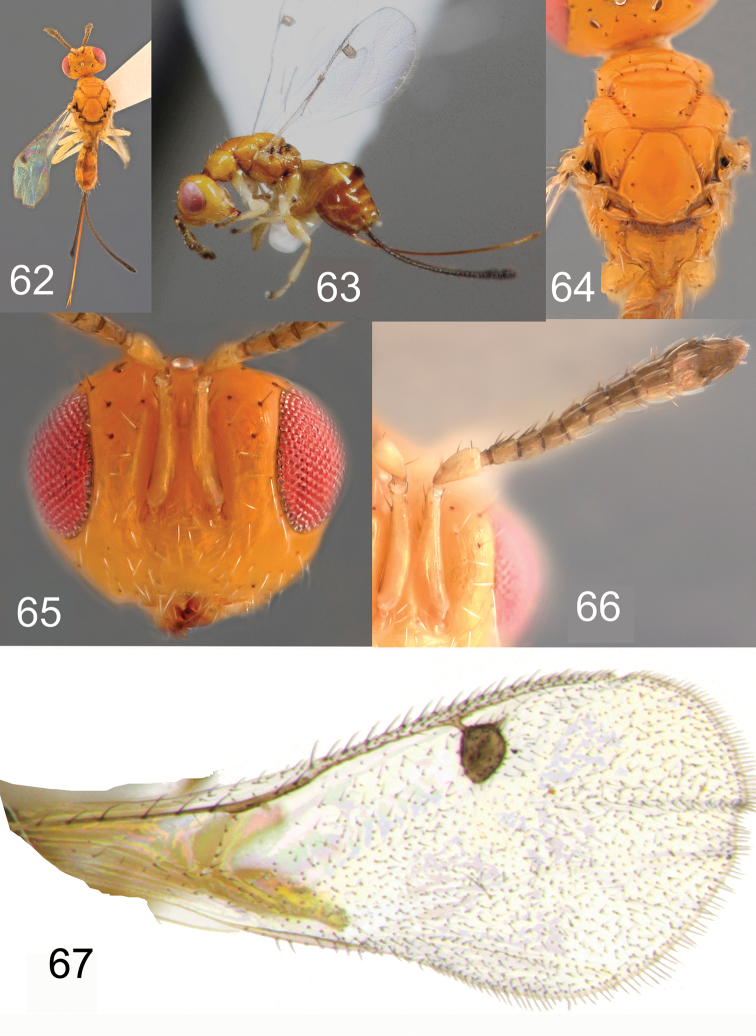
*Megastigmus icipeensis* Roques & Copeland, sp. n. female. **62** dorsal view of the body **63** lateral view of body **64** dorsal view of thorax **65** front view of head **66** antenna **67** forewing.

**Figures 68–74.**
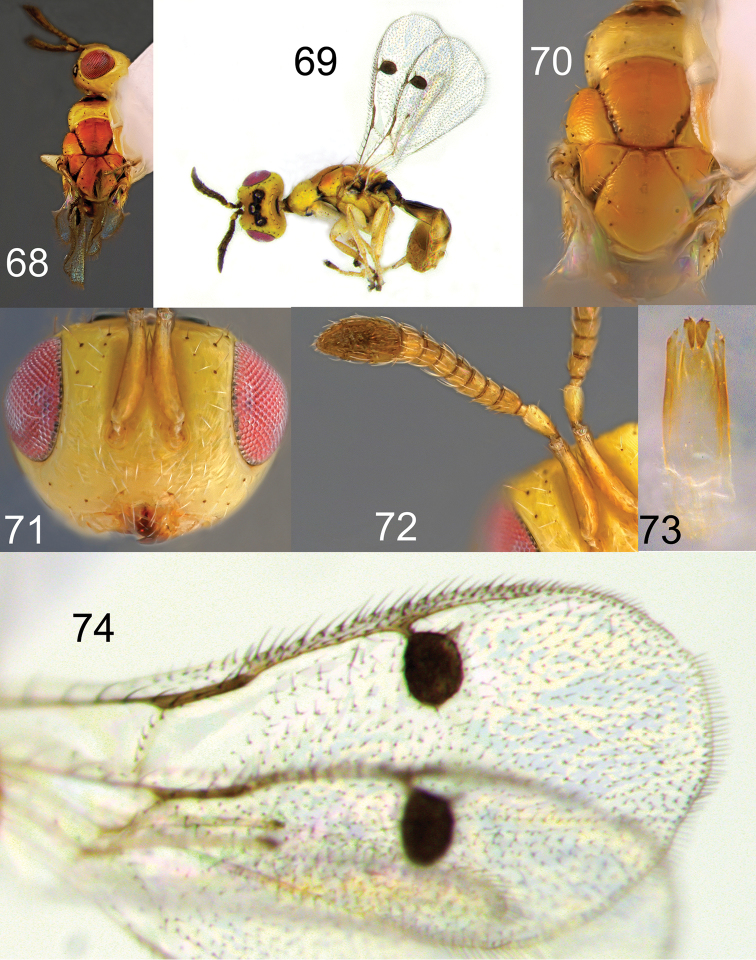
*Megastigmus icipeensis* Roques & Copeland, sp. n. male. **68** dorsal view of the body **69** lateral view of body **70** dorsal view of thorax **71** front view of head **72** antenna **73** forewing **74** genitalia.

**Figures 75–80.**
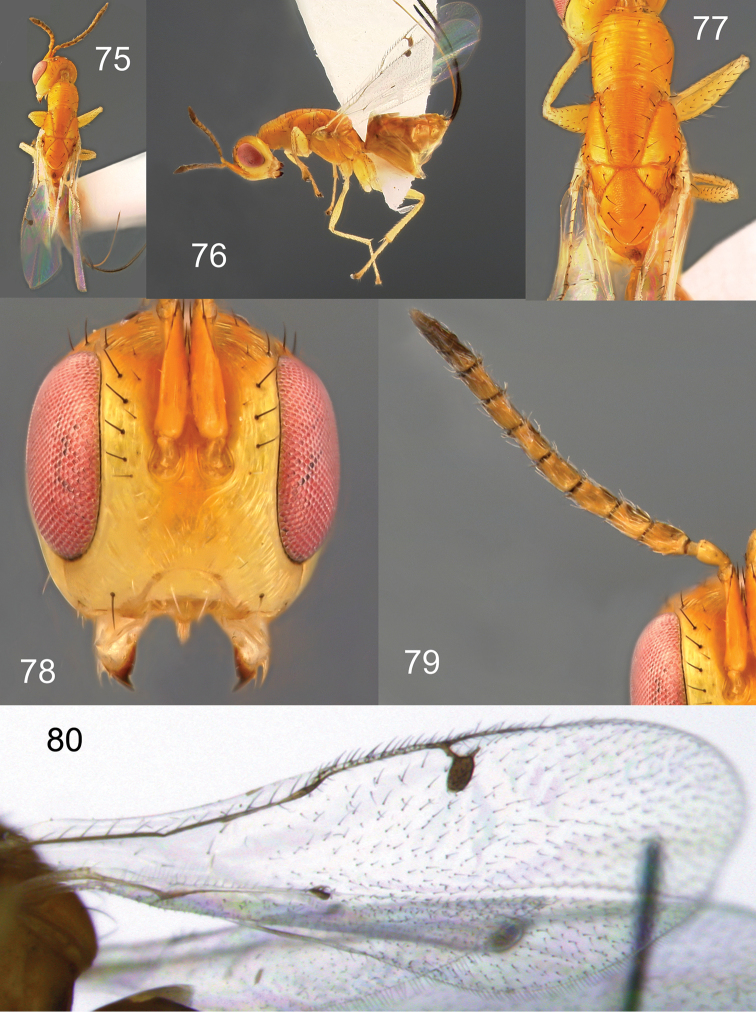
*Megastigmus laventhali* Roques & Copeland, sp. n. female. **75** dorsal view of the body **76** lateral view of body **77** dorsal view of thorax **78** front view of head **79** antenna **80** forewing.

**Figures 81–87.**
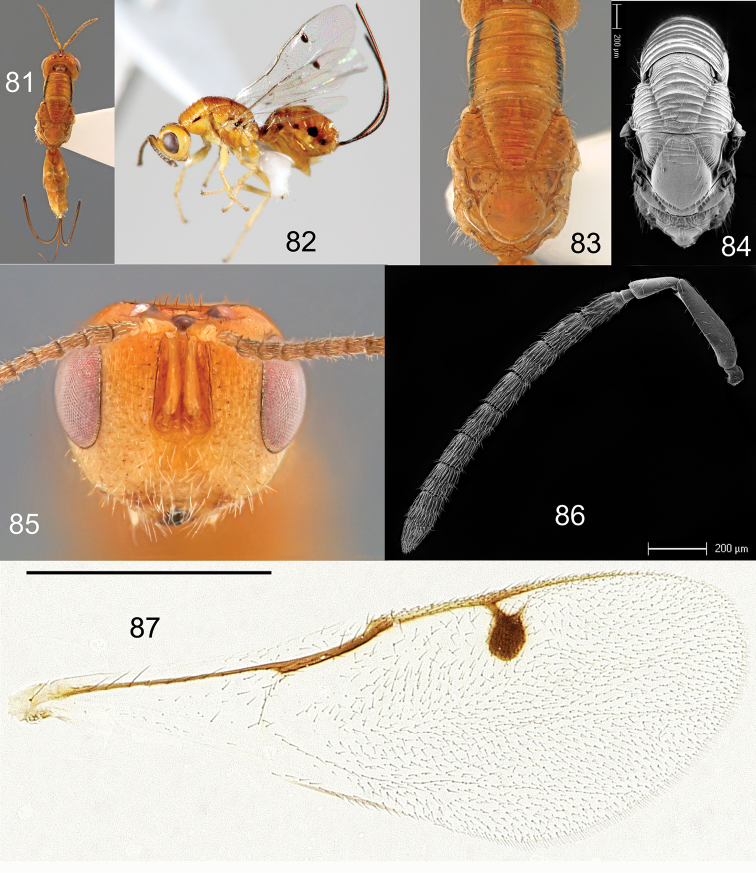
*Megastigmus grewianae* Roques & Copeland, sp. n. female. **81** dorsal view of the body **82** lateral view of body **83** dorsal view of thorax **84** electroscan of dorsal view of thorax **85** front view of head **86** electroscan of antenna **87** forewing.

**Figures 88–95.**
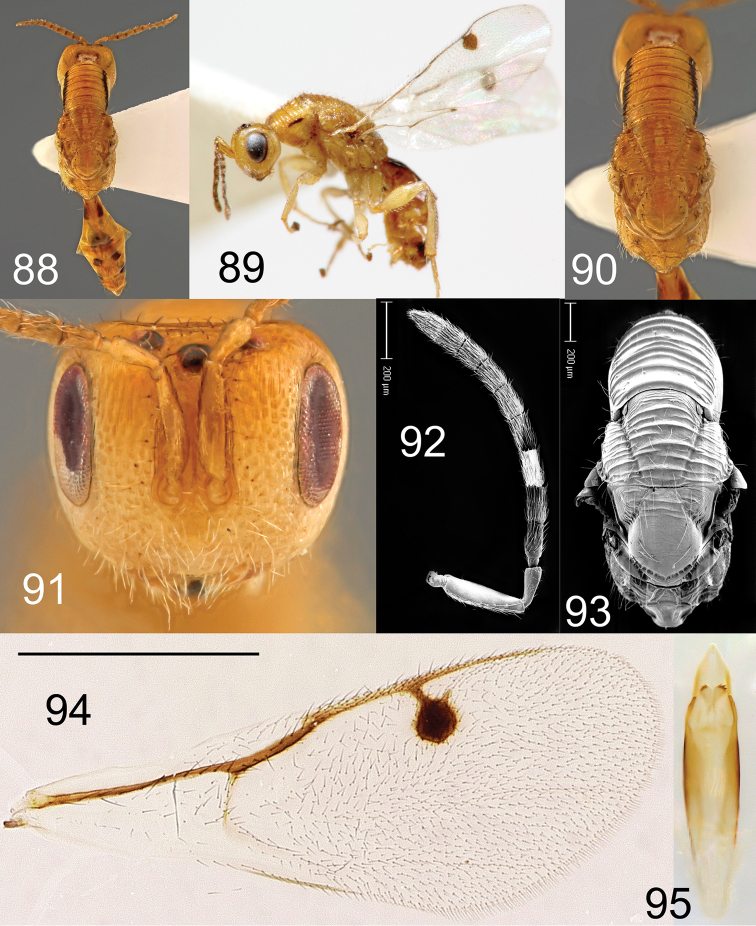
*Megastigmus grewianae* Roques & Copeland, sp. n. male. **88** dorsal view of the body **89** lateral view of body **90** dorsal view of thorax **91** front view of head **92** electroscan of antenna **93** electroscan of dorsal view of thorax **94** forewing **95** genitalia.

**Figures 96–101.**
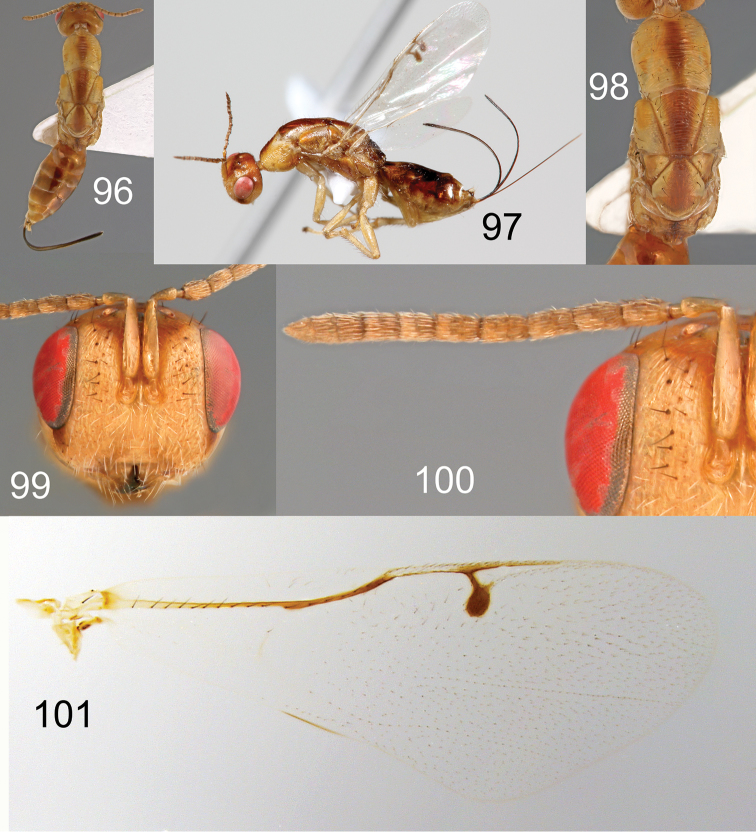
*Megastigmus lanneae* Roques & Copeland, sp. n. female. **96** dorsal view of the body **97** lateral view of body **98** dorsal view of thorax **99** front view of head **100** antenna **101** forewing.

**Figures 102–108.**
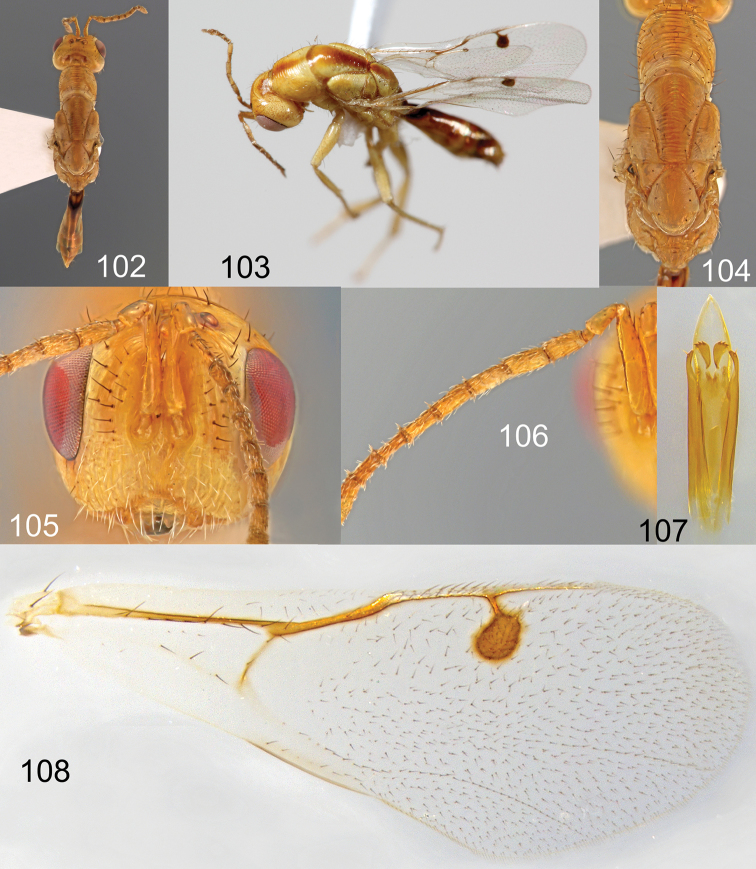
*Megastigmus lanneae* Roques & Copeland, sp. n. male pale form. **102** dorsal view of the body **103** lateral view of body **104** dorsal view of thorax **105** front view of head; **106** antenna **107** genitalia **108** forewing.

**Figures 109–115.**
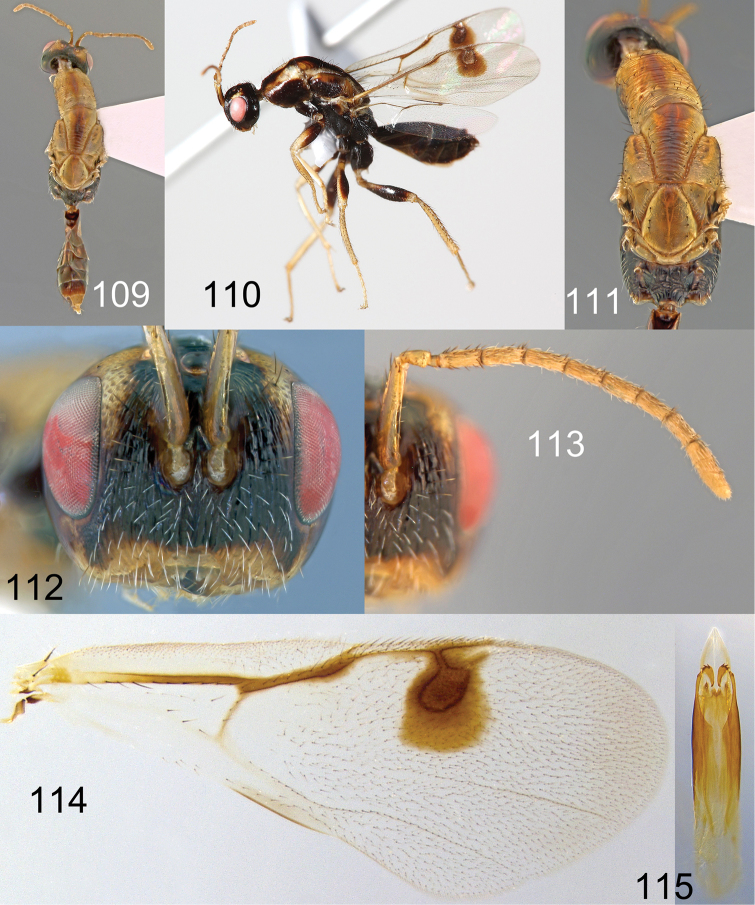
*Megastigmus lanneae* Roques & Copeland, sp. n. dark form. **109** dorsal view of the body **110** lateral view of body **111** dorsal view of thorax **112** front view of head; **113** antenna **114** forewing **115** genitalia.

**Figures 116–121.**
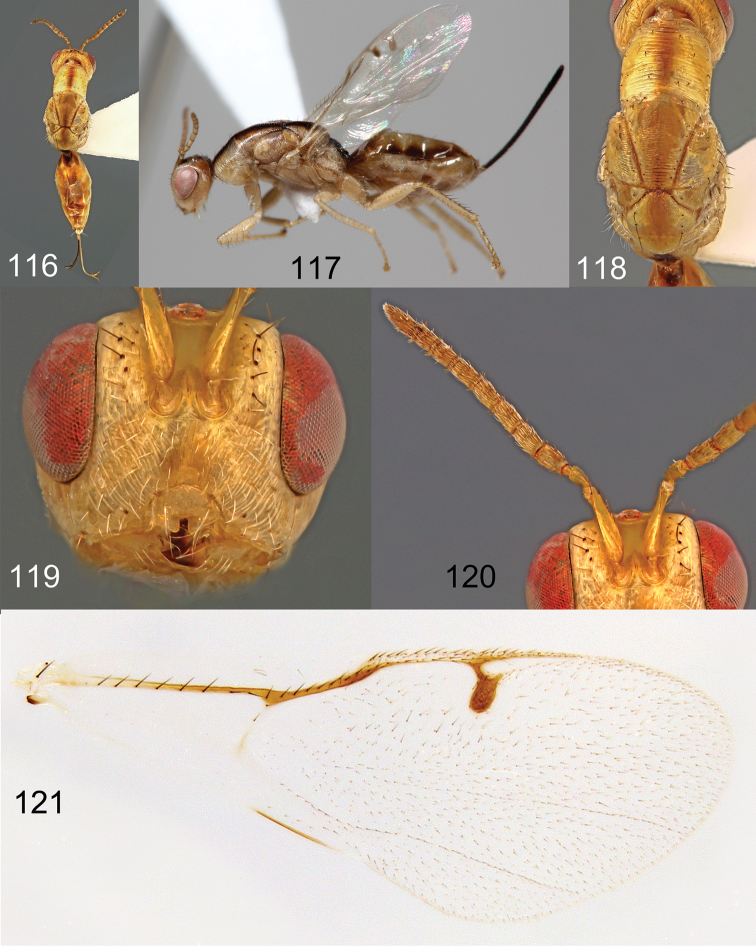
*Megastigmus hypogeus* Hussey female. **116** dorsal view of the body **117** lateral view of body **118** dorsal view of thorax **119** front view of head **120** antenna **121** forewing.

**Figures 122–128.**
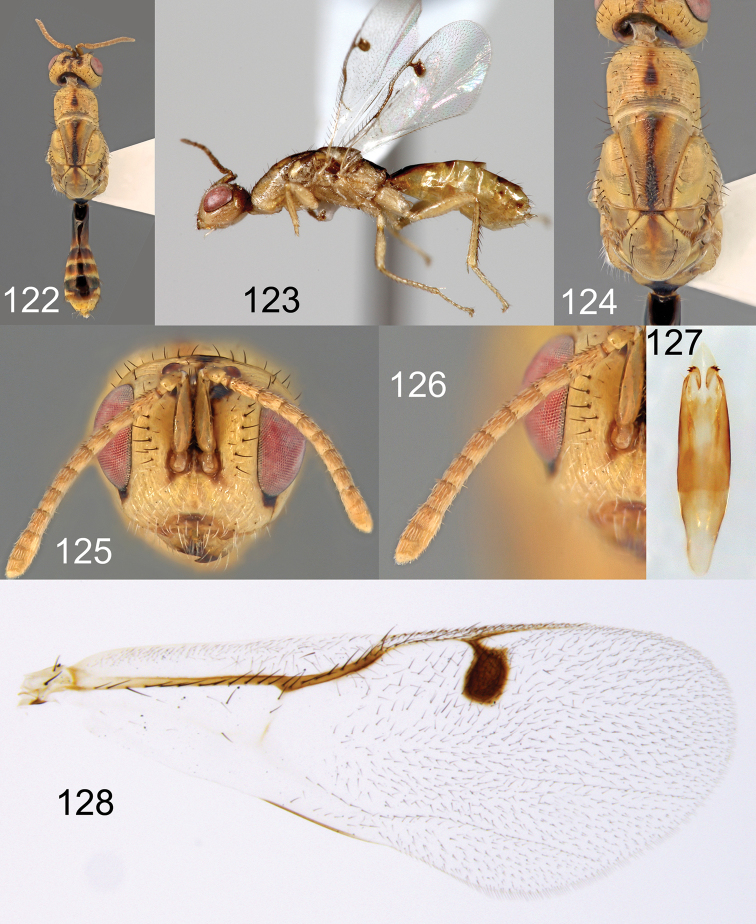
*Megastigmus hypogeus* Hussey male pale form. **122** dorsal view of the body **123** lateral view of body **124** dorsal view of thorax **125** front view of head; **126** antenna **127** genitalia **128** forewing.

**Figures 129–135.**
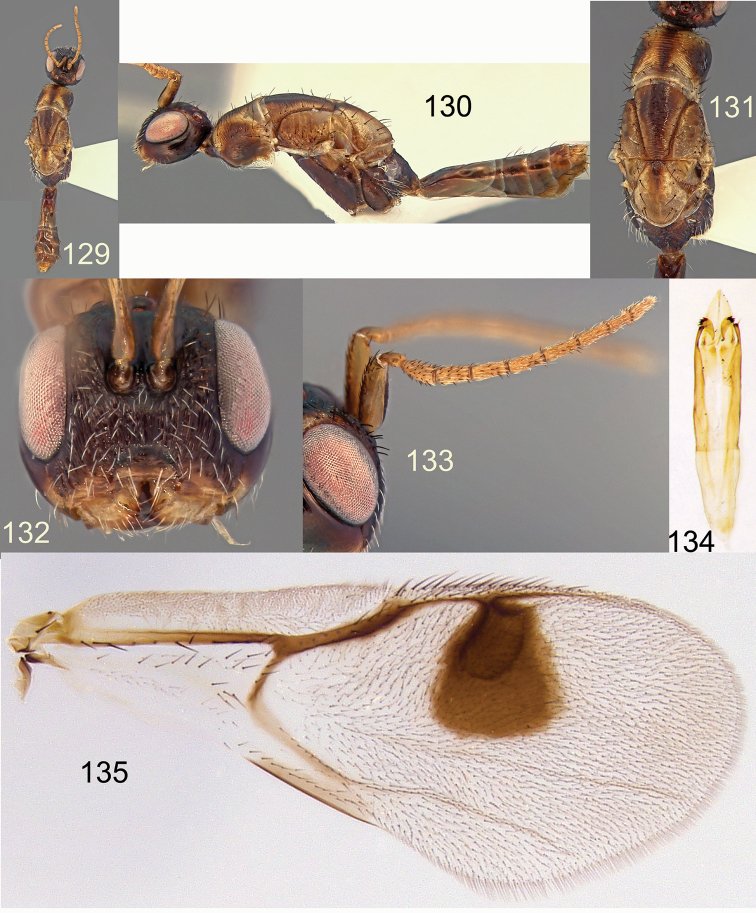
*Megastigmus hypogeus* Hussey male dark form. **129** dorsal view of the body **130** lateral view of body **131** dorsal view of thorax **132** front view of head; **133** antenna **134** genitalia **135** forewing.

**Figures 136–141.**
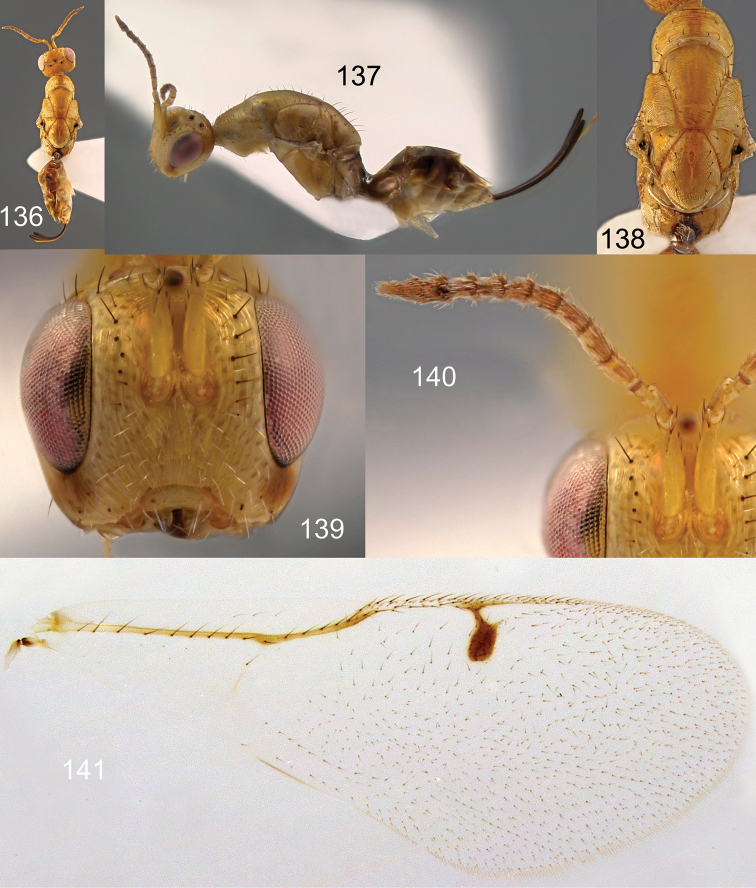
*Megastigmus pistaciae* Walker female. **136** dorsal view of the body **137** lateral view of body **138** dorsal view of thorax **139** front view of head **140** antenna **141** forewing.

**Figures 142–149.**
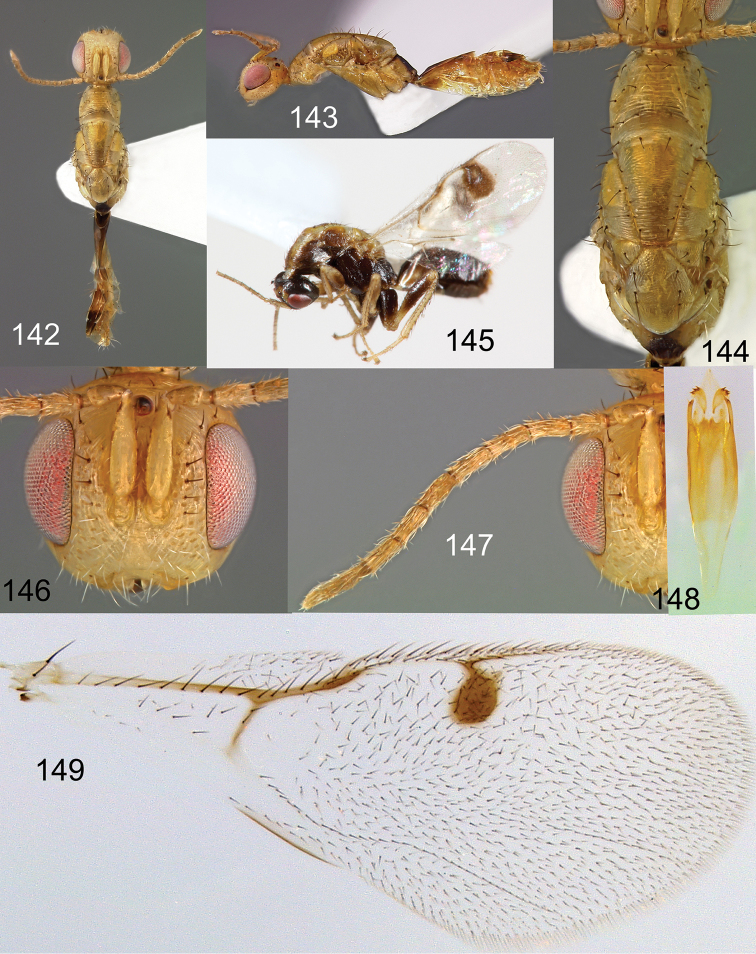
*Megastigmus pistaciae* Walker male. **142** dorsal view of the body (pale form) **143** lateral view of body (pale form) **144** dorsal view of thorax(pale form) **145** lateral view of body (dark form) **146** front view of head (pale form); **147** antenna (pale form) **148** genitalia (pale form) **149** forewing (pale form).

**Figures 150–155.**
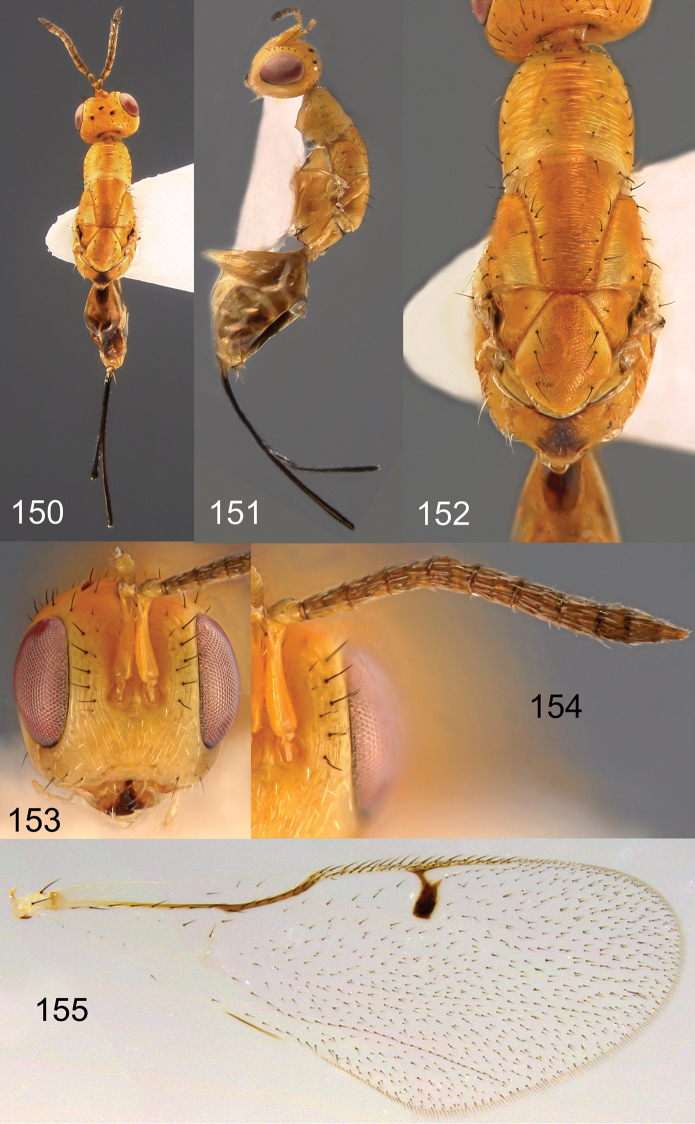
*Megastigmus transvaalensis* Hussey female. **150** dorsal view of the body **151** lateral view of body **152** dorsal view of thorax **153** front view of head **154** antenna **155** forewing.

**Figures 156–162.**
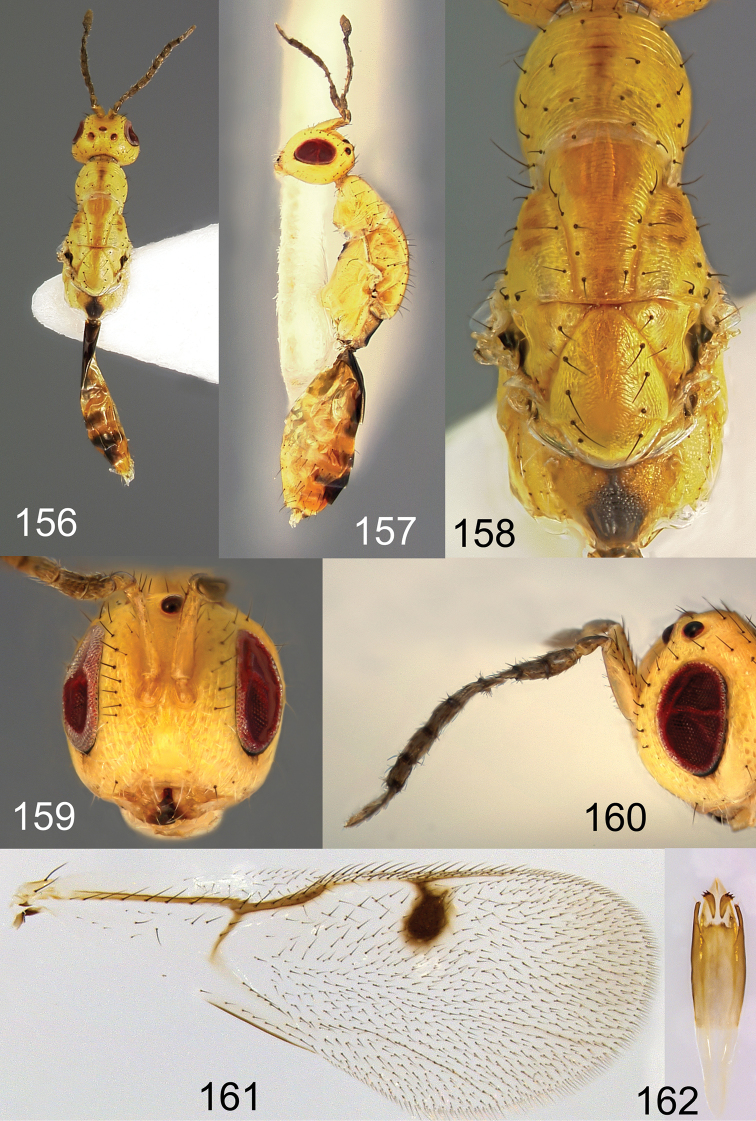
*Megastigmus transvaalensis* Hussey male. **156** dorsal view of the body **157** lateral view of body **158** dorsal view of thorax **159** front view of head; **160** antenna **161** forewing **162** genitalia.

**Figures 163–167.**
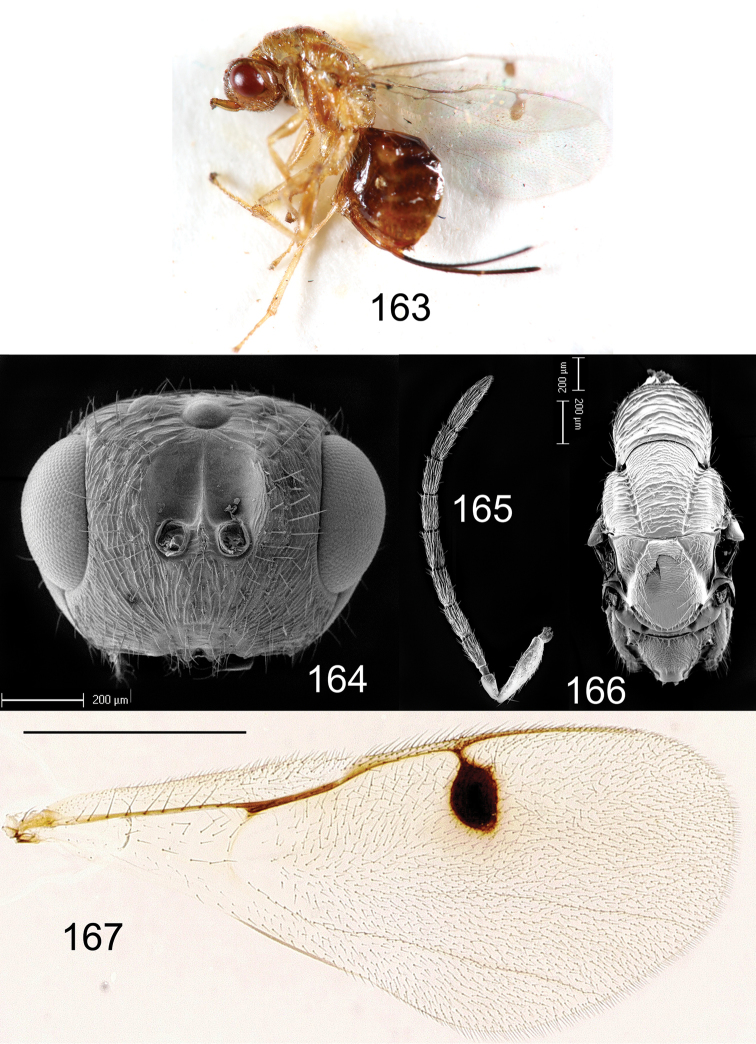
*Megastigmus somaliensis* Hussey. **163** lateral view of female body **164** electroscan of front view of male head **165** electroscan of male antenna **166** electroscan of dorsal view of male thorax **167** male forewing.

**Figure 168.**
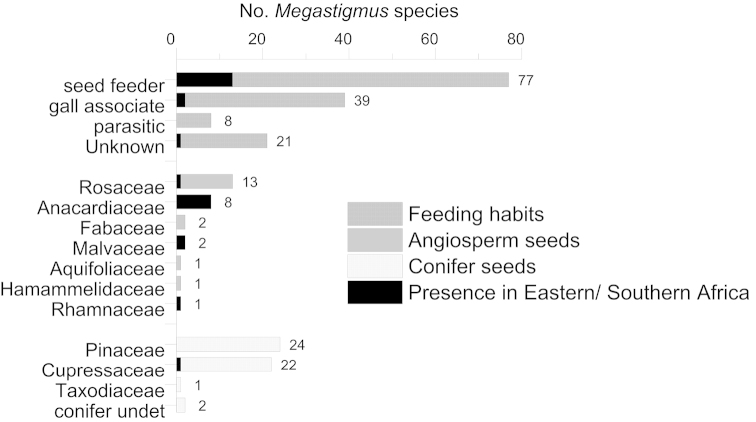
Updated synthesis of biological habits of the world *Megastigmus* species recorded to date with a detail of the species present in Eastern and Southern Africa. The four bars on the top detail the chalcid feeding regimes. The following ones present the host plant families colonized by the phytophagous seed chalcids, splitted into angiosperms and conifers.

## Species description

### Megastigmus helinae

Taxon classificationAnimaliaHymenopteraTorymidae

Roques & Copeland sp. n.

http://zoobank.org/A2A398C5-E7D8-4B8E-B858-2136275BC907

#### Type material.

**Holotype** ♀, Kenya, Scandent climber, Rift Valley Prov. Matthews Range, 1.1777°N, 37.3141°E, 1342m, 16 Jan 2004, ex. *Helinus integrifolius* fruits, A&M coll. N°2692, R. Copeland leg. (NMKE).

**Paratypes.** 2♀♀, same collection data as holotype (ARC, 1 destroyed); 2♂♂, same collection data as holotype (NMKE, ICIPE).

#### Description.

**Holotype** ♀. A small species with body length 3.2 mm (without ovipositor); length of ovipositor sheaths 0.8 mm. Body colour nearly entirely orange-yellow (Figures 15, 16). Head orange with a black ring around each ocellus. Pilosity on lower face pale, but black hairs on malar space, gena, and on dorsum of head, with a row of long hairs on vertex behind ocelli. Antenna entirely yellow. Pronotum orange; remainder of thorax orange except a bisinuate black band along pronotum suture at the anterior part of mid-lobe of mesoscutum (Figure 16), and two small black spots at wing insertions. Pilosity black on thorax dorsum; sparse long hairs on pronotum and mid-lobe of mesoscutum along notauli; scutellum with 3 pairs of black hairs on the sides. Legs entirely pale yellow. Forewing stigma brown without infuscation; basal cell well defined, with disc including 8 long setae, closed by a basal setal line with 8 long hairs and a costal setal line with 6 long hairs; coastal cell with several rows of small setae (Figure 21). Propodeum orange with a narrow, curved black stripe on the center of its anterior part; propodeum hairs black. Gaster entirely orange, with black hairs on sutures. Ovipositor sheaths black, very short, only 0.6 × as long as gaster, 0.2 × as long as body (Figure 16).

Head transverse (Figure 18), width: height ratio: 1.3 in dorsal view; POL: OOL: 0.8; torulus 1.2 × as long as wide; inter-antennal area as broad as torulus width; scrobe elongate, ca. 2.3 × as long as wide; Scape 1.2 × as long as combined length of pedicel, anellus and F1 (Figure 19); scape 0.8 × as long as combined length of pedicel, anellus, F1 and F2; anellus subquadrate; F1 1.2 × as long as pedicel, 1.9 × as long as wide; F2 1.8 × as long as wide; following funicular segments progressively tending to subquadrate from F4 on, with F7 1.3 × as long as wide. Pronotum, mid- and lateral lobes of mesoscutum, with coarse transverse carinae (Figure 20); axilla with longitudinal striae; mid-lobe of mesoscutum 1.1 × as long as scutellum. Scutellum 1.3 × as long as wide, with irregular transverse striae; frenum 0.3 × as long as scutellum length, mostly smooth with a few longitudinal carinae on sides. Forewing stigma oval, about 1.5 × as long as wide (Figure 21); upper part of stigmal vein elongate, 0.4 × as long as stigma length; uncus 0.6 × as long as upper part of stigmal vein; marginal vein 0.8 × as long as postmarginal vein. Propodeum with coarse longitudinal carinae, and a median carina interrupted in the middle (Figure 20).

**Male.** Body colour similar to that of female (Figures 22, 23), orange-yellow with a few black markings: a ring around each ocellus, a transverse fuzzy band on the center of the anterior part of propodeum, a large spot on T3 at gaster insertion, a comma- like spot on the lateral sides of T4, and a smaller, rounded, brownish spot on the lateral sides of the three following tergites. Pilosity as in female. Forewing stigma brown without infuscation (Figure 28); basal cell well defined, the disc with 11 long setae, closed by a basal setal line with 7 long setae and a costal setal line with 6 long setae; coastal cell with several rows of small setae.

Head rectangular (Figure 25), width: height ratio: 1.5 in dorsal view; POL: OOL 0.9; torulus 1.2 × as long as wide; inter-antennal area 0.9 × as wide as torulus width; scrobe 2.5 × as long as wide. Scape 1.1 × as long as combined length of pedicel, anellus and F1 (Figure 26); scape 0.8 × as long as combined length of pedicel, anellus, F1 and F2; F1 1.3 × as long as pedicel, 2.1 × as long as wide; F2 2.0 × as long as wide; following funicular segments also elongate, with F7 1.5 × as long as wide. Pronotum, mid- and lateral lobes of mesoscutum, with coarse transverse carinae (Figure 27); axilla with longitudinal striae; mid-lobe of mesoscutum short, 0.9 × as long as scutellum. Scutellum 1.2 × as long as wide, with irregular, transverse striae; frenum 0.3 × as long as scutellum length, mostly smooth with a few longitudinal carinae on sides; Forewing stigma more rounded than in female, about 1.4 × as long as wide (Figure 28); upper part of stigmal vein elongate, 0.5 × as long as stigma length; uncus 0.7 × as long as upper part of stigmal vein; marginal vein 0.8 × as long as postmarginal vein. Propodeum with coarse longitudinal carinae, and short median carina in its upper part (Figure 27). Gaster conspicuously petiolate (Figure 23). Genitalia with rather small aedeagus, its part above digitus only 1.2× as long as digitus length; digitus with only 2 teeth (Figure 29).

**Variation.** The transverse brown band at the anterior part of mid-lobe of mesoscutum is less marked in female paratypes. In males, the lateral black-brown spots could be absent on T5–T7.

#### Host plants.

*Helinus integrifolius* (Rhamnaceae). Probably a seed feeder, based on its place in the molecular phylogeny of *Megastigmus* spp. (Figure 14). Nevertheless we only sequenced one specimen and it would be necessary to analyze other samples in order to clarify the phylogenetic position, which appeared to vary depending on the marker.

#### Distribution.

Known only from the Matthews Range, Kenya where it was observed to emerge from 9.8 % of the collected fruits (Table 3).

#### Etymology.

Named after its host plant.

#### Diagnosis and comments.

This is the only species of *Megastigmus* found to date to emerge from fruits of the Rhamnaceae. Females are easily distinguished from those of other species present in the region by the very small ovipositor associated with an orange body colour. Genitalic characters differentiate males *Megastigmus helinae* by the presence of only two teeth on the digitus whereas the other species bear three teeth except *Megastigmus icipeensis* but the phallobase of the latter species is less elongate (Figure 74).

### Megastigmus smithi

Taxon classificationAnimaliaHymenopteraTorymidae

Roques & Copeland sp. n.

http://zoobank.org/3A22FAA9-278F-4741-8288-123D074A276B

#### Type material.

**Holotype** ♀, Kenya, Coast Province, Sagalla Mountain, 3.47864°S, 38.57463°E, 1090m, 22 Sep 2014, ex. *Lannea* cf. *schimperi* fruits, CHIESA coll. N°343, R. Copeland leg. (NMKE).

**Paratypes** 2♀♀, same collection data as holotype (1♀ ICIPE; 1♀ARC).

#### Description.

**Holotype** ♀. Medium-sized species, body length (without ovipositor) 4.6 mm; length of ovipositor sheaths 0.8 mm. Body colour orange-yellow and brown (Figures 30, 31, 32). Head with face yellow except a blackish infuscation extending from base of eye along malar line, a large brown spot covering most of head dorsum and extending to the top of the scrobe on the face and to the temple on the back; parascrobal area and gena yellow. Pilosity on lower face pale; upper face and dorsum of head with conspicuous black hairs (Figure 33). Antenna brownish except scape entirely yellow and pedicel yellow underneath. Pronotum yellow with a brown, irregular median band extending from anterior to posterior suture (Figure 32); mid-lobe of mesoscutum yellow with a large triangular brownish band in the middle, narrowing from anterior to posterior suture; lateral lobe of mesoscutum with a large orange rectangular spot at apex; axilla with a large longitudinal orange band on its lateral half (Figure 32). Scutellum with a large brownish band, covering about half of the surface until frenum; frenal area quite completely brownish, with a narrow yellow band on borders; pleura and ventrum yellow except a large dark orange spot along mesopleural line. Pilosity on thorax entirely black; 3 pairs of black hairs on scutellum. Legs entirely pale yellow except claws brown.

Forewing stigma brown without infuscation; basal cell indistinct, lacking setae, no setae on basal setal line and costal setal line (Figure 35). Propodeum with a large, oval longitudinal black band in its middle (Figure 30, 32); callus and metapleuron yellow with the suture dark orange. Propodeum hairs entirely pale; Gaster brown, T3 orange with a black spot at insertion, following tergites with a dark brown band covering the middle part (Figure 30), lateral parts orange with a whitish rectangular spot on the side. Pilosity on gaster black. Ovipositor sheaths black, very short, only 0.4 × as long as gaster, 0.2 × as long as body (Figure 31).

Head transverse (Figure 33), width: height ratio: 1.5; POL: OOL: 1.4. Scape 1.2 × as long as combined length of pedicel, anellus and F1 (Figure 34); scape 0.9 × as long as combined length of pedicel, anellus, F1 and F2; anellus subquadrate; F1 1.1 × as long as pedicel, 1.5 × as long as wide; F2 1.5 × as long as wide; following funicular segments longer than wide, F7 1.3 × longer than wide. Pronotum, mid- and lateral lobes of mesoscutum with transverse carinae; axilla striated. Mid-lobe of mesoscutum 1.1 × as long as scutellum. Scutellum 1.3 × as long as wide, with irregular transverse striae; frenum 0.4 × as long as scutellum length; completely covered with irregular, concentric wrinkles. Forewing stigma oval elongate, 1.6 × as long as wide (Figure 35); upper part of stigmal vein relatively elongate, 0.4 × as long as stigma length; uncus short, 0.4× as long as upper part of stigmal vein; marginal vein 0.9× as long as postmarginal vein. Propodeum with coarse longitudinal carinae, and conspicuous spiracles.

**Variation**: Body length from 4.5–4.9 mm, ovipositor sheaths from 0.8–0.9 mm. No variation was observed in colour or pilosity.

**No males reared.**

#### Host plants.

*Lannea* cf. *schimperi* (Anacardiaceae). Like other species also reared from Anacardiaceae fruits, e.g., *Megastigmus pistaciae* and *Megastigmus transvaalensis*, it is probably a seed chalcid.

#### Distribution.

Known only from Sagalla Mountain, Taita Hills, Kenya. Specimens emerged from only 0.4% of sampled fruits (Table 3).

#### Etymology.

Named in honour of Robert J. Smith, noted musicologist and exceptional left fielder.

#### Diagnosis and comments.

Its comparatively very short ovipositor, nearly 2 × smaller than gaster length, allows to separate easily this species from the other ones developing in Anacardiacae fruits and showing resembling body colour patterns but a comparatively much longer ovipositor, quite as long as gaster length (*Megastigmus ozoroae*) or longer (*Megastigmus lanneae*; *Megastigmus hypogeus*).

### Megastigmus copelandi

Taxon classificationAnimaliaHymenopteraTorymidae

Roques & Copeland sp. n.

http://zoobank.org/7FD3FBF4-C166-4172-B1F0-98CEFA571BC8

#### Type material.

**Holotype** ♀: Kenya, Coast Province, Taita hills, Mwatate area, 3.48940°S, 38.33298°E, 1050m, 4 Feb 2012, ex. *Grewia tephrodermis* seeds, CHIESA coll. N°98, R. Copeland leg. (NMKE)

**Paratypes**: 2 ♀♀, same collection data as holotype (1 ♀ RSC; 1 ♀ ARC)

#### Description.

**Holotype** ♀. Small species with body length (without ovipositor) 2.6 mm; length of ovipositor sheaths 1.1 mm. Body colour entirely orange to dark orange except a few black patterns including contours of each ocellus, a spot at wing insertions, contour of propodeal spiracle, rounded spots on the lateral parts of T3, a lunule on the middle of T4, and 2 elongated blackspots on the lateral sides of T5 (Figures 36, 37). Head and gaster dark orange contrasting with the paler orange of the thorax (Figure 37). Pilosity on head entirely pale (Figure 39). Antenna brownish. Thoracic dorsum with long black hairs but lateral parts with pale hairs; scutellum with 3 lateral pairs of long black hairs (Figure 38). Propodeum with pale hairs, coxa and legs with pale hairs. Forewing stigma black without infuscation; basal cell with two small setae, closed by a basal setal line with five long setae and a costal setal line with seven long setae (Figure 41). Ovipositor sheaths black, 0.9 × as long as gaster, 0.4 × as long as body (Figure 37).

Head rounded, width: height ratio: 1.3×; POL: OOL 1.0; inter-antennal area as broad as torulus width; scrobe short, ca. 2.6 × as long as wide. Scape 1.2 × as long as combined length of pedicel, anellus, and F1 (Figure 40); scape 0.8 × as long as combined length of pedicel, anellus, F1 and F2; anellus subquadrate; F1 1.1 × as long as pedicel, 1.6 × as long as wide; F2 1.9 × as long as wide; following funicular segments becoming increasingly elongate, with F7 2.0 × as long as wide. Pronotum, mid- and lateral lobes of mesoscutum, and axilla with very coarse transverse carinae; mid-lobe of mesoscutum 1.1 × as long as scutellum. Scutellum 1.2 × as long as wide, with irregular transverse striae; frenum 0.4 × as long as scutellum length, densely wrinkled. Stigma elongate, oval-oblique, about 1.4 × as long as wide (Figure 41); upper part of stigmal vein short, 0.3 × as long as stigma length; uncus 0.9 × as long as upper part of stigmal vein; marginal vein 0.9 × as long as postmarginal vein. Propodeum with cross- striae tending to reticulate in the anterior part but without median carina.

**Variation.** Females range in length from 2.6–2.7 mm. In one paratype, gaster is dark brown. Wing chaetotaxy is variable with basal setal line with 5–6 long setae and costal setal line with 3–6 setae.

No males reared.

#### Host plants.

*Grewia tephrodermis* (Malvaceae).

#### Distribution.

Known only from the Mwatate area, Taita Hills, Kenya. *Megastigmus copelandi* emerged from 0.7% of collected fruits (Table 3). Probably a seed feeder.

#### Etymology.

Named after Dr. Robert Copeland, who collected the specimen.

#### Diagnosis.

The combination of an almost entirely orange body, pale pilosity on the head, and an ovipositor a bit shorter than gaster length readily distinguishes this species from other Afrotropical *Megastigmus*, and from *Megastigmus grewianae*, in particular, which also attacks *Grewia* fruits. The latter species is much larger (4.1 mm vs. 2.6–2.7) and has an ovipositor ca. 1.8 × longer than gaster length (Figure 82) vs. 0.9 × in *Megastigmus copelandi*.

### Megastigmus ozoroae

Taxon classificationAnimaliaHymenopteraTorymidae

Roques & Copeland sp. n.

http://zoobank.org/DB27B108-C725-4654-938C-2C1ED26FAA59

#### Type material.

**Holotype.** ♀, Kenya, Nyanza Province, Sindo-Mbita Road, 0.4849°S, 34.1765°E, 1205m, 11 Dec 2004, A&M coll. N°3064, ex. *Ozoroa insignis* ssp *reticulata*, R. Copeland leg. (NMKE)

**Paratypes.** 8 ♀♀, same collection data as holotype; 6♂♂, same collection data as holotype, except 19 Dec 2004, A&M coll. N°3077 (2♀♀, 1♂, NMKE; 2♀♀, 1♂ RSC; 2♀♀, 1♂ ARC, 1♀, 1♂ ICIPE, 1♀, 1♂ SAMC)

#### Additional material examined.

23♀♀, 11♂♂ same collection data as holotype, except 19 Dec 2004, A&M coll. N°3077 (11♀♀, 5♂♂ RSC; 12♀♀, 6♂♂ ARC)

#### Descriprion.

**Holotype** ♀. Large species, body length (without ovipositor) 4.8 mm; length of ovipositor sheaths 1.5 mm. Body colour yellow and black (Figures 42–44). Head pale yellow except a conspicuous black ring around occipital carina extending on vertex in a distinct median band reaching the inter-antennal area, malar sulcus blackish, and a black dot on each side of clypeus (Figure 45). Pilosity pale on lower face; upper face with a row of long black hairs along parascrobal area and on dorsum of head (Figure 45). Antenna brown with scape and pedicel yellow beneath. Pronotum pale yellow- grey with a large, nearly rectangular, longitudinal black spot in the middle, its colour turning to brownish at the posterior end (Figure 44). Remainder of thorax mostly pale yellow with conspicuous darker patterns: a large brownish spot covering most of the mid-lobe of mesoscutum (except the lateral parts) including a large black triangle at the anterior suture extending in a median, distinct black line to the posterior suture, a large triangular brownish spot covering most of the scutellum, a rectangular orange spot on the upper part of the lateral lobe of mesoscutum followed by a grey spot of same size, a large orange spot on the axilla, all sutures brownish- black along prepectus and lateral panel of pronotum, mid-lobe of mesoscutum, lateral lobe of mesoscutum and scutellum, and a brownish oval spot including a small triangular black spot in the middle of the mesepimeron. Metanotum yellow with a black transverse line interrupted in the middle. Pilosity on thorax dark, with 5 pairs of conspicuous setae on the lateral yellow sides of the posterior part of mid-lobe of mesoscutum, 4 pairs on lateral sides of scutellum, and 1 pair of large setae at the anterior corner of axilla.

Legs pale yellow except claws brownish; coxae with numerous pale hairs extending from small black dots, especially on hind coxa; femora with small black hairs; tibiae with 3 rows of long black hairs. Forewing stigma brown without infuscation; basal cell only partly closed, the disc with 5 very small setae; basal line with 2 setae; no setae on costal line; 4 setae in coastal cell (Figure 47). Propodeum pale yellow with black markings including a large central band extending in a lateral line along the suture with metanotum (Figure 44), a longitudinal spot along suture between callus and propodeum, and the sutures of metapleuron and callus. Pilosity pale on propodeum. Gaster brown- black with yellowish sides; T3 black on dorsum; the 4 following tergites with large transverse black bands progressively narrowing and becoming brownish on sides; a row of long black hairs along lateral parts of all tergite sutures; sterna pale yellow; Ovipositor sheaths black, short, only 0.8 × as long as gaster, 0.3 × as long as body (Figure 43).

Head about 1.1 × as wide as long in front view (Figure 45). POL: OOL: 1.5; inter-antennal area as broad as torulus width; scrobe elongate, ca. 3 × as long as wide. Scape 1.2 × as long as combined length of pedicel, anellus, and F1 (Figure 46); scape 0.8 × as long as combined length of pedicel, anellus, F1 and F2; F1 1.3 × as long as pedicel, 1.7 × as long as wide; F2 1.6 × as long as wide; following funicular segments tending to subquadrate from F3, with F7 as long as wide. Pronotum with coarse transverse carinae; mid-lobe of mesoscutum and axilla with finer cross-striae; mid-lobe of mesoscutum 1.3 × as long as scutellum; scutellum 1.1× as long as wide, noticeably covered with longitudinal, irregular wrinkles; frenum quite indistinct from scutellum, roughly 0.4 × as long as scutellum length, completely covered with irregular wrinkles. Forewing stigma oval- oblique, about 1.6 × as long as wide (Figure 47); upper part of stigmal vein elongate, 0.4 × as long as stigma length; uncus short, 0.4 × as long as upper part of stigmal vein; marginal vein 0.8 × as long as postmarginal vein. Propodeum with a short median carina in its anterior part and cross- striae on its upper part, the lower part smooth and shining.

**Variation.** Females range in length from 3.5 to 4.8 mm. Five of the examined specimens out of 14 have lateral lobes of mesoscutum and scutellum pale yellow. The black spots on pronotum and mid-lobe of mesoscutum as well as the other black markings are absent in one specimen, except at base of eye, on the suture of the lateral lobe of mesoscutum and on the central part of propodeum. The general colour of this specimen is orange, with the gaster brownish and not blackish in its middle part, the brownish colour beginning only at T5. Another specimen has the central part of propodeum completely shining black. The relative length of the ovipositor sheaths varies between 0.7 to 0.8 × the gaster length, and 0.3–0.4 × the body length.

**Males.** They are highly variable in colour, and two extreme forms can be distinguished with intermediates.

*Pale form*. Body length from 4.3 to 5.7 mm. Body colour pale yellow and black (Figures 48–50). Head with a large black spot extending from lower face to frons and vertex, and laterally to the lower part of eye which is entirely surrounded by a black line, and malar space (Figure 51); yellow only on gena, temples, base of clypeus and lower part of malar space and parascrobal area; ocellar area black, with anterior ocellus covered with black but not the lateral part of posterior ocelli; black extending in a conspicuous black ring around occiput. Pilosity on face pale, but conspicuous black hairs on parascrobal area, temple, gena and dorsum of head (Figure 51). Antenna yellow. Pronotum yellow with a longitudinal black band in the center, narrowing towards mid-lobe of mesoscutum. Remainder of thorax yellow, except mid-lobe of mesoscutum with a large triangular black-brown spot covering the anterior part, prolongated in its center by an irregular longitudinal black line, narrowing in the middle, and expanding to the posterior part of mid-lobe of mesoscutum (Figure 50). Anterior of scutellum with a brownish; a conspicuous square-shaped grey spot in the posterior part of lateral lobe of mesoscutum; axilla yellow; lateral panel of pronotum black; prepectus mostly black with a few yellow infuscations near the suture of lower mesepisternum and prothoracic spiracle; upper and lower mesepisternum mostly black except in the center a yellow band extending to median coxa; lateral part of upper and lower mesepimeron yellow; all sutures black along mid-lobe of mesoscutum, lateral lobe of mesoscutum, and scutellum. Thoracic ventrum mostly black. Pilosity on thorax consisting of long black hairs, including 5 pairs on the postero- lateral parts of mid-lobe of mesoscutum, 1 pair on axilla, and 3 pairs on scutellum.

Legs yellow expect anterior part of coxae with large black spots (extending to the posterior part in fore coxa), and small brownish spots at femur insertion, 3 rows of large hairs on tibia. Forewing stigma brown without infuscation (Figure 54); basal cell partly closed, with 8 setae on disc; basal setal line with 2 long setae, but costal setal line quite indistinct with 3 small setae; coastal cell with more than 20 long setae in several rows in the apical 1/2 to 2/3 . Propodeum yellow- grey with a black band along the anterior suture black and a large oval black spot medially (Figure 50); callus and metapleuron yellow but the sutures black. Propodeum hairs pale. Gaster colour mostly black-brown; T3 petiolate and black; T4 with anterior part brown, followed by a large yellow band; T5 with the anterior part brown, followed by a small yellow band; T6 with a large black spot in form of shield; T7 brown; T8- T9 yellow with a brown lateral band. Long black hairs on tergite sutures. Genitalia with elongate aedeagus, its part above digitus about 1.8 × as long as digitus length; digitus compressed, about 2.2 × longer than its maximum width, with 3 teeth (Figure 53).

Head about 1.2 × as wide as long in front view. POL: OOL: 2.0; inter-antennal area as broad as torulus width; scrobe short, ca. 1.5 × as long as wide. Scape 1.3 × as long as combined length of pedicel, anellus, and F1 (Figure 52); scape 0.9 × as long as combined length of pedicel, anellus, F1 and F2; F1 1.5 × as long as pedicel, 1.7 × as long as wide; F2 1.6 × as long as wide; following funicular segments elongate, only tending to subquadrate from F7. Pronotum with coarse transverse carinae, mid-lobe of mesoscutum and axilla with transverse striae finer than on pronotum. Mid-lobe of mesoscutum 1.3 × as long as scutellum length; Scutellum 1.2 × as long as wide, noticeably covered with longitudinal, irregular wrinkles; frenum nearly indistinct from scutellum, roughly 0.4 × as long as scutellum length, completely covered with irregular, arching, concentric wrinkles. Forewing stigma oval- oblique, about 1.4 × as long as wide (Figure 54); upper part of stigmal vein very short, only 0.3 × as long as stigma length; uncus short, 0.6 × as long as upper part of stigmal vein; marginal vein 0.9 × as long as postmarginal vein. Propodeum with strong, irregular striae medially

*Dark form*. Larger than pale form, with body length 5.0 to 6.5 mm. Body colour mostly black (Figures 55–57) with a conspicuous, large triangular infuscation around the forewing stigma (Figure 61). Head entirely black except a triangular dark grey spot along parascrobal area (Figure 58). Thorax black except a triangular grey spot on sides of pronotum posteriorly, a narrow yellowish line on the sides of mid-lobe of mesoscutum and on lateral sutures of lateral lobes of mesoscutum, axillae yellowish (Figure 57). Antero-lateral part of scutellum yellowish but frenum black. Metanotum with a transverse yellow line. Propodeum entirely black. Legs mostly black (Figure 56); coxae black except the posterior part yellowish; mid- and hind femora black except the basal and apical part brownish; fore femur with the apical part yellow; tibiae and tarsi yellow. Forewing with basal cell closed, disc with 10 strong setae; basal setal line with 2 long setae, costal setal line with 8 small setae; coastal cell with more than 100 small setae in several rows in the apical 1/2 to 2/3 (Figure 61). Thoracic sculpture, pilosity and genitalia (Figure 60) same as in pale forms.

*Intermediates*. In some specimens, black patterns on thorax are limited to a sharp median line on pronotum and mid-lobe of mesoscutum, and to the sutures of lateral panel of pronotum, prepectus, and tegula. These specimens also show a grey spot on lateral lobes of mesoscutum and a brownish median line on scutellum. Gaster is mostly black with a few yellow patterns as follows: an annelation at the base of T3, the sides of T4, a small lateral spot on T5 and the last two segments. Forewing stigma not infuscated in these individuals. In some others, the lateral parts of thorax are black (panel of pronotum, prepectus, mesepisternum, and upper mesepimeron) except lower mesepimeron and metapleuron and callus yellow (with black spots on callus), and scutellum is black at the base with the remainder dark brown. In these individuals, the forewing stigma is infuscated. Pilosity may include 5–6 pairs of setae on mid-lobe of mesoscutum and 3–4 pairs on scutellum.

#### Host plants.

*Ozoroa insignis* (Anacardiaceae). Its position in the molecular phylogeny is close to those *Megastigmus* species known to be seed feeders in Anacardiaceae, strongly suggesting it is also a seed feeder. Although the presence of numts was noticed in the analyzed specimen of *Megastigmus ozoroae* it belonged whatever the marker (short mitochondrial or nuclear fragment) to the “Anacardiaceae clade” confirming the existence of a strong monophyletic clade, the most divergent from the others (Figure 14).

#### Distribution.

Known only from western Kenya, near Lake Victoria. In one collection, 8.1% of fruits were infested (Table 3).

#### Etymology.

Named after the genus of the host plant.

#### Diagnosis.

Females are easily distinguished from those of other species observed to emerge from fruits of Anacardiaceae by the relatively small ovipositor, nearly as long as gaster length whereas it is at least 1.2 × the gaster length in *Megastigmus hypogeus* (Figure 117), *Megastigmus lanneae* (Figure 97), *Megastigmus laventhali* (Figure 76), *Megastigmus pistaciae* (Figure 137), *Megastigmus thomseni* (see Hussey 1956a) and *Megastigmus transvaalensis* (Figure 151). In contrast, the ovipositor of *Megastigmus smithi* is significantly shorter (0.4 × the gaster length; Figure 31). Genitalic characters differentiate male *Megastigmus ozoroae* from those of males described for other species that feed in Anacardiaceae; the aedeagus part above digitus being significantly longer in *Megastigmus ozoroae* (1.8 × as long as digitus length) than in *Megastigmus hypogeus* (1.2–1.3×; Figures 127, 134), *Megastigmus pistaciae* (0.8×; Figure 148) and *Megastigmus transvaalensis* (0.5×; Figure 162). The aedaegus dimension compared to digitus is similar in *Megastigmus lanneae* but the phallobase is more elongate in the latter species (Figures 107, 115), being 2.8× longer than wide vs. 2.6× in *Megastigmus ozoroae*.

### Megastigmus icipeensis

Taxon classificationAnimaliaHymenopteraTorymidae

Roques & Copeland sp. n.

http://zoobank.org/4EE8D716-63AB-48F4-8B9E-E6277136C6D8

#### Type material.

**Holotype** ♀, Kenya, Nairobi Province, ICIPE Campus, Kasarani, 1.2232°S,36.8965°E, 1600m elevation, Malaise trap, meadow in degraded shrub-/grassland, 24 Feb to 3 Mar 2014, R. Copeland leg. (NMKE)

**Paratypes** 4♀♀, the same as holotype (1♀ NMKE; 1♀ RSC; 1♀ ARC; 1♀ ICIPE).

#### Additional material.

3♂♂, collected together with the four females (2♂♂ RSC; 1♂ ARC).

#### Description.

**Holotype** ♀. Very small species with body length (without ovipositor) 1.2 mm; length of ovipositor sheaths 0.9mm. Body colour nearly entirely orange-yellow (Figures 62–64). Head colour orange, except mouthparts brown (Figure 65). Pilosity on lower face pale; dorsum of head with conspicuous black hairs: a row along the internal side of each eye from parascrobal area to temple, a row along the occiput, and three isolated hairs around each of the lateral ocelli; two interocellar bristles. Antenna dark- brown except scape and pedicel yellow underneath (Figure 66). Thorax entirely orange except a small black spot on axilla at wing insertions and on lateral panel of metanotum (Figure 64). Pilosity on thorax mostly pale; sparse long hairs on pronotum, especially along the suture with mid-lobe of mesoscutum; 3 pairs of pale hairs on the lateral side of mid-lobe of mesoscutum, very close to suture with the lateral lobe; 2 pairs of pale hairs on scutellum. Legs entirely pale yellow except claws brown; Forewing stigma brown without infuscation; basal cell without setae, closed by a basal setal line with 5 setae and a costal setal line with 3 setae (Figure 67). Propodeum orange with a transverse black stripe along the suture with metanotum (Figure 64). Propodeum hairs pale; Gaster orange, with four large transverse orange-brown bands along the anterior sutures of the tergites 4–8; pilosity on gaster black. Ovipositor sheaths black, 1.6 × longer than gaster, 0.7× as long as body (Figure 63).

Head about 1.2 × as wide as long in front view (Figure 65). POL large, POL: OOL: 2.7. Scape 1.1 × as long as combined length of pedicel, anellus, and F1 (Figure 66); scape 0.9 × as long as combined length of pedicel, anellus, F1 and F2; pedicel elongate, 1.5× longer than F1; F2 subquadrate, small, 0.8 × as long as F1; following funicular segments subquadrate from F2. Pronotum, mid-lobe of mesoscutum, axilla and scutellum with fine transverse striae. Mid-lobe of mesoscutum conspicuously short, only 0.9 × as long as scutellum length; scutellum 1.1 × as long as wide; frenum roughly 0.4 × as long as scutellum length, with the same sculpture as the remainder of scutellum. Propodeum with a zig-zag median carina. Forewing stigma oval, without infuscation, about 1.4 × as long as wide (Figure 67); upper part of stigmal vein short, 0.2 × as long as stigma length; uncus as long as upper part of stigmal vein; marginal vein 0.8 × as long as postmarginal vein.

**Variation.** Body ranges 1.2–1.3 mm with ovipositor up to 1.2 mm. Ovipositor length varies from 0.7 to 0.9 × the body length, and from 1.6 to 2.0 × the gaster length. No variation in colour and pilosity.

**Male.** In the absence of molecular analyses, the males caught in Malaise traps along with the four female *Megastigmus icipeensis* could not be attributed with certainty to this species but noticeable convergences in morphology make it probable. Body length 1mm. Same body colour as in female of *Megastigmus icipeensis* (Figures 68–70) except a paler yellow pronotum and some additional black patterns on head and thorax: an irregular spot surrounding the three ocelli, an annelation at insertion of pronotum, a darkening of the lateral sutures of mid-lobe of mesoscutum, a narrow transverse band on lateral panel of metanotum, and a transverse band covering the base of propodeum and extending in its middle into a large longitudinal spot in shape of inverted Y (Figures 62, 64). Gaster petiolate, mostly brown- black on dorsum: T3 black, T4 brown black with a large transverse yellow band distally, T5 similar but the yellow band narrower, T6 brown-black, T7 yellow.

Legs yellow with the two last tarsal segments and claws black. Pilosity pale on head and thorax, showing the same setal pattern as in female *Megastigmus icipeensis*, including the interocellar brisles and 2 pairs of pale hairs on scutellum (Figure 70). Forewing stigma without infuscation (Figure 73); basal cell closed, but without setae on disc; basal setal line with 6 setae, costal setal line with 3 long setae; costal cell with only 3 small setae in the apical 1/2 to 2/3.

Head transverse, about 1.3 × as wide as long in front view (Figure 71) . OOL short, POL: OOL: 2.8. Scape 1.2 × as long as combined length of pedicel, anellus, and F1 (Figure 72); scape as long as combined length of pedicel, anellus, F1 and F2; pedicel elongate, 1.8 × longer than F1; F2 subquadrate, small, 0.8 × as long as F1; following funicular segments also subquadrate. Pronotum, mid-lobe of mesoscutum, axilla and scutellum finely transversely striated. Mid-lobe of mesoscutum conspicuously short, only as long as scutellum length; scutellum 1.1 × as long as wide; frenum roughly 0.4 × as long as scutellum length, with the same sculpture as the rest of scutellum. Propodeum with an irregular median carina. Forewing stigma nearly rounded, only 1.3 × as long as wide (Figure 74); upper part of stigmal vein very short, only 0.2 × as long as stigma length; uncus 1.5 × longer than upper part of stigmal vein; marginal vein 1.1 × longer than postmarginal vein. Genital digitus with only 2 teeth (Figure 73).

#### Host plants.

Unknown. In the molecular study, *Megastigmus icipeensis* did not group with other taxa, but the phylogenetic position (Figure 14) and the genetic distances between this species and the other groups led to suggest that it could be a seed-feeder associated with another plant family than those identified in this paper.

#### Distribution.

Only known from the collection site in a degraded meadow near a small stream.

#### Etymology.

Named after the institution on whose grounds the species was collected, the campus of the International Centre of Insect Physiology and Ecology (ICIPE), Nairobi.

#### Diagnosis.

The species can be easily distinguished from other tiny *Megastigmus* spp. known from the Afrotropical region by the pale bristles on the thoracic dorsum whereas they are mostly black in *Megastigmus zebrinus* (Grissell, 2006), and by its orange body whereas *Megastigmus pretorianensis* is mostly black with brown areas (Doǧanlar 2015). In contrast to most other species, the male genital digitus bears only two teeth such as in *Megastigmus helinae* but the phallobase of the latter species is more elongate (Figure 29).

### Megastigmus laventhali

Taxon classificationAnimaliaHymenopteraTorymidae

Roques & Copeland sp. n.

http://zoobank.org/81764A75-CF49-4AB8-977F-497F56F9A6DA

#### Type material.

**Holotype** ♀, Kenya, Coast Province, Base of Kasigau Mountain, 3.81301°S, 38.64050°E, 619m, 5 Mar 2012, ex. *Lannea schweinfurthii* fruits, CHIESA coll. N°141, R. Copeland leg. (NMKE);

**Paratype 1**♀ Kenya, Coast Province, Funzi island, 4.57776°S, 39.44127°E, near sea level, Malaise trap in mixed grass and woodland, 24 to 28 Jul 2012, R. Copeland leg. (ICIPE).

#### Description.

**Holotype** ♀. Medium-sized species with body length (without ovipositor) 4.0 mm; length of ovipositor sheaths 3.4 mm. Body colour predominantly orange (Figures 75–77). Head entirely orange (Figure 78); pilosity on lower face pale; upper face and dorsum of head with conspicuous black hairs. Antenna light brown except scape entirely orange (Figure 79). Thorax entirely light orange except a small black spot at wing insertion (Figure 77). Pilosity on thorax black; a row of 6 black hairs along each lateral suture of mid lobe of mesoscutum; 3 pairs of black hairs on scutellum (Figure 77). Legs entirely pale yellow except claws brown. Forewing stigma brown without infuscation; basal cell indistinct, with two small setae on disc; basal setal line with 1 seta; costal setal line without setae; costal cell without setae (Figure 80). Propodeum light orange with a darker longitudinal line medially (Figure 75); propodeal spiracle very conspicuous; propodeum hairs entirely pale. Gaster dark orange, darker than propodeum; pilosity on gaster black. Ovipositor sheaths black, long, 1.9 × as long as gaster, 0.8 × as long as body, 0.9 × as long as thorax plus gaster (Figure 76).

Head rounded, width: height ratio: 1.0 (Figure 78); POL:OOL: 1.1. Scape 1.1 × as long as combined length of pedicel, anellus and F1 (Figure 79); scape 0.8 × as long as combined length of pedicel, anellus, F1 and F2; anellus subquadrate; F1 1.1 × as long as pedicel, twice as long as wide; F2 1.1 × as long as 1^st^ funicular segment; following funicular segments longer than wide, with F7 1.4 × longer than wide. Pronotum, mid- and lateral lobes of mesoscutum with coarse transverse carinae; axilla with finer striae; mid-lobe of mesoscutum elongate, 1.3 × as long as scutellum; scutellum 1.2 × as long as wide, reticulate; frenum 0.4 × as long as scutellum length, reticulate. Forewing stigma oblique, nearly rectangular, 1.5 × as long as wide (Figure 80); upper part of stigmal vein 0.4 × as long as stigma length; uncus relatively elongate, 0.8 × as long as upper part of stigmal vein; marginal vein 0.9 × as long as postmarginal vein. Propodeum with coarse longitudinal carinae and conspicuous spiracles.

**Variation.** In the paratype, the basal cell presents two long setae on disc, and the basal setal line has 1 seta.

**No males reared.**

#### Host plants.

*Lannea schweinfurthii* (Anacardiaceae). Probably a seed feeder but it could not be integrated in the molecular analysis because too few specimens were available.

#### Distribution.

Known from the base of Kasigau Mountain, a Taita Hills outlier. Only 1 of 168 fruits (0.6%) was infested (Table 3). Also collected in a Malaise trap sample from Funzi Island, just off the southeast coast of Kenya (Figure 2).

#### Etymology.

Named in honour of Leo Laventhal, historian, Yiddish scholar and union man.

#### Diagnosis.

Females can be separated easily from those of other species associated with fruits of Anacardiaceae by the combination of a predominantly orange body and a relatively long ovipositor, at least 1.8 × as long as the gaster. In most of the other species, body colour combines dark and yellow patterns (*Megastigmus hypogeus*, *Megastigmus ozoroae*, *Megastigmus lanneae*, *Megastigmus smithi*). For the remaining species that have a predominantly orange body colour, the ovipositor length is at most 1.4 × gaster length (*Megastigmus pistaciae*, *Megastigmus thomseni* and *Megastigmus transvaalensis*).

### Megastigmus grewianae

Taxon classificationAnimaliaHymenopteraTorymidae

Roques & Copeland sp. n.

http://zoobank.org/5AA95119-9BC7-4DF3-AE4E-3CB3409147EB

#### Type material.

**Holotype** ♀, Kenya, Coast Province, Arabuko-Sokoke forest, 3.2997°S, 39.9869°E, 55 m, 17 Feb 2000, ex. *Grewia stuhlmanii* fruits, Coll. N° Kip-356, R Copeland leg. (NMKE)

**Paratypes**: Kenya, 1♀, 1♂, same collection data as holotype (RSC); 2♀♀, 1♂, same collection data as holotype (ARC); 1♀, 7♂♂, Eastern Province, Tsavo, 2.6760°S, 38.3325°E, 638 m, 20 Feb 2000, ex. fruits of *Grewia tephrodermis*, A&M Coll. N°514, R. Copeland leg. (1♀, 3♂♂ NMKE, 4♂♂ ICIPE); 1♀, Coast Province, Kasigau Mountain, 3.82700°S, 38.64875°E, 1065 m, Malaise trap in campsite clearance, 19 May to 2 Jun 2011, R. Copeland leg. (SAMC); 1♀, Eastern Province, Kasaala area, 2.07486°S, 38.22530°E, 741 m, Malaise trap, 28 Nov. to 4 Dec 2013, leg. J. Bukhebi & R. Copeland (ICIPE).

#### Additional material in alcohol.

Kenya, 7♀♀, 1♂, same as holotype (ARC); 1♀, Eastern Province, Tsavo, 2.6760°S; 38.3325°E, 638 m, 20 Feb 2000, ex. fruits of *Grewia tephrodermis*, Coll. #514, R. Copeland leg. (ARC)

#### Description.

**Holotype** ♀. Body length (without ovipositor) 4.1mm; length of ovipositor sheaths 3.0 mm. Body colour orange with some darker patterns (Figures 81–83). Head colour orange with a very narrow occipital black line and outer part of ocelli black. Pilosity on face pale, dark on dorsum of head but hairs not strong (Figure 85). Antenna brownish except scape and pedicel yellowish (Figure 85). Pronotum orange with a conspicuous longitudinal black stripe running for 7/8 of the lateral part of the pronotum, interrupted just before the suture with mid-lobe of mesoscutum (Figures 82–83). Remainder of thorax orange except black spots on the posterior part of axilla at wing insertion. Pilosity on thorax black but hairs not strong; a few on pronotum; 5 pairs on latero-posterior part of mid-lobe of mesoscutum; 4 pairs on lateral lobe of mesoscutum along suture with mid-lobe of mesoscutum; 4 pairs on axilla. Scutellum orange with 5 pairs of lateral hairs with conspicuous insertion dots (Figures 83–84).

Legs entirely pale yellow except claws brown, coxae with conspicuous hair dots. Forewing stigma brown without infuscation; basal cell closed, with 13 setae on disc ; basal setal line with 6 long setae; costal setal line with 16 small setae, costal cell with 11 setae in 2 rows (Figure 87). Propodeum dark orange, with a small oblique brownish spot around the spiracle; not wrinkled transversally; propodeum hairs dark. Gaster orange with two lateral rows composed of 5 brown elongated spots, one on each of the first five segments, placed latero-medially, the third spot shaped like a comma, larger than the others, followed in size by the 4^th^ one. Ovipositor sheaths black, 1.8 × as long as gaster, 0.7 × as long as body (Figure 82).

Head rounded, width: height ratio: 1.1 × (Figure 85); POL: OOL: 1.3; torulus ca. 1.2 × as long as wide; inter-antennal area much smaller (0.6×) than torulus width; scrobe elongate, ca. 3.3× as long as wide. Scape elongate, 1.2 × as long as combined length of pedicel, anellus and F1 (Figure 86); scape 0.8 × as long as combined length of pedicel, anellus, F1 and F2; Pedicel elongate, 1.3× longer than F1; anellus subquadrate; F1 1.8 × as long as wide, shorter than the other segments, 0.8 × shorter than F2; F2 1.9 × as long as wide; following funicular segments same as F2, except F7 and F8 tending to subquadrate, with F7 1.4 × as long as wide. Pronotum with 8 very strong transverse carinae (Figure 84), mid- lobe of mesoscutum with 7 coarse transverse carinae extending onto lateral lobes; axillae with 3 weaker, longitudinal, carinae. Mid-lobe of mesoscutum 1.2 × as long as scutellum length. Scutellum 1.2 × as long as wide, with transverse, irregular striae anteriorly; frenum smooth, 0.3 × as long as scutellum length. Stigma oval, about 1.2 × as long as wide (Figure 87); upper part of stigmal vein very short, 0.3 × as long as stigma length; uncus comparatively elongate, 1.2 × longer than upper part of stigmal vein; marginal vein 0.8 × as long as postmarginal vein. Propodeum reticulate with 2 oblique carinae diverging from the center of the anterior suture (Figure 84).

**Male.** Body length 4.2 mm; Body colour, pilosity and black patterns similar to female (Figures 88–91) except gaster with 6 rows of brown spots laterally on dorsum; those on the first two segments elongate with the second longer, the 3 following more rounded, comma-like, and the last a simple spot (Figure 88). Forewing stigma brown without infuscation; basal cell closed, with 21 setae on disc; basal setal line with 7 setae; costal setal line with 21 setae; costal cell with 20 setae arranged in 3 rows (Figure 94). Thorax pilosity as in female.

Head subquadrate, width: height ratio: 1.1 × (Figure 91); POL: OOL 1.3; torulus ca. 1.2× as long as wide; inter-antennal area short, 0.6× as broad as torulus width; scrobe elongate, ca. 3.9× as long as wide; eyes protruding. Scape as long as combined length of pedicel, anellus and F1 (Figure 92); scape 0.7× as long as combined length of pedicel, anellus, F1 and F2; pedicel 1.2× longer than F1; anellus subquadrate; F1 2.1× as long as wide; F2 1.9× as long as wide; following funicular segments similar to F2, only F7 tending to suquadrate (1.2× as long as wide). Pronotum with coarse transverse carinae as in female but irregular; some carinae interrupted in their middle on mid-lobe of mesoscutum (Figure 93); axilla with curved longitudinal striae; mid-lobe of mesoscutum 1.1× as long as scutellum length. Scutellum 1.2 × as long as wide, with transverse striae in the anterior part only, then smooth; frenum entirely smooth, 0.2 × as long as scutellum length (Figure 93). Forewing stigma subquadrate, 1.1 × as long as wide (Figure 94); upper part of stigmal vein short, 0.3 × as long as stigma length; uncus as long as upper part of stigmal vein; marginal vein 0.8 × as long as postmarginal vein. Propodeum with several oblique, irregular carinae (Figure 93). Genitalia with aedeagus relatively elongate, its part above digitus about 1.6 × as long as digitus length; digitus enlarged at its extremity, only twice as long than its maximum width, with 3 teeth (Figure 95).

**Variation.** Females range in length from 3.5 to 4.0mm. The number of lateral spots on gaster varies from 6 (with two on T3) to 2 (only the large ones remaining), the 3 last lateral spots sometimes fused into a line. Pilosity on thorax paler in one female paratype. Males range in length from 4.1 to 4.5 mm. The spots on gaster sometimes larger, the pairs of lateral hairs on scutellum ranging up to 6 in both sexes.

#### Host plants.

*Grewia stuhlmannii*, *Grewia tephrodermis* (Malvaceae). Probably a seed feeder, based on its place in the molecular phylogeny of *Megastigmus* spp. (Figure 14). Sequencing of four specimens from Arabuko-Sokoke forest showed they differed by less than 0.3% in COI, confirming the validity of the single specimen that we used in the analyses, even if the position remains unresolved. A large number of species of *Grewia* coexist in East Africa (Brink and Achigan-Dako 2012) and it would be interesting to find if other *Megastigmus* species or sub-species are able to attack their seeds.

#### Distribution.

Known from dry *Acacia*/*Commiphora* savanna, moderately wet mid-altitude mountain forest, and bush associated with costal forest. Adults emerged from 2.7% of *Grewia stuhlmannii* fruits and 13% of *Grewia tephrodermis* fruits (Table 3).

#### Etymology.

Named after the genus of its host plant.

#### Diagnosis.

Females are easily separated from those of *Megastigmus copelandi*, the other species reared from *Grewia* fruits, by their significantly larger size (4.1 mm vs. 2.6–2.7) and the relatively longer ovipositor (ca. 1.8 × longer than gaster length vs. 0.9 × in *Megastigmus copelandi*). Females and males are also easily distinguished from the other species of Afrotropical *Megastigmus* by the unique pattern of a longitudinal black stripe extending 7/8 of the length of the side of the pronotum (Figures 82–83, 89–90).

### Megastigmus lanneae

Taxon classificationAnimaliaHymenopteraTorymidae

Roques & Copeland sp. n.

http://zoobank.org/A19A4C74-A315-46B7-90B9-828196351FAF

#### Type material.

**Holotype** ♀: Kenya, Eastern Province, Machakos, 1.5609°S, 37.2338°E, 1586m elevation, 3 May 2005, ex. *Lannea rivae* fruits, Coll. N°3109, R. Copeland leg. (NMKE)

**Paratypes** 8♀♀, 12♂♂, same collection data as holotype (2♀♀, 2♂♂ NMKE; 2♀♀, 4♂♂ RSC; 2♀♀, 2♂♂ ARC; 1♀, 2♂♂ ICIPE; 1♀, 2♂♂ SAMC);

#### Additional material in alcohol.

Kenya; 7 ♀♀ same collection data as holotype; 2♀♀, same collection data as holotype except 25 Nov. 2004, ex. *Lannea rivae* seeds, Coll. N°3042, R. Copeland leg. (ARC).

#### Description.

**Holotype** ♀. Large species, body length (without ovipositor) 5.4 mm; length of ovipositor sheaths 4.2 mm. Body colour yellow and brownish (Figures 96–98). Head yellow with the following dark patterns (Figure 99): a brownish band from frons to occiput, covering the interocellar area, base of eye at malar line black, malar line with a brownish infuscation, temple with a brownish infuscation. Pilosity on face and gena pale, dark hairs on temple, vertex and parascrobal area (Figure 99). Antenna light brownish, scape and pedicel yellow underneath (Figure 100). Pronotum yellow with a large, nearly rectangular, dark median band extending from anterior to posterior suture (Figure 98); mid-lobe of mesoscutum with a large triangular band covering most of the surface, narrowing from anterior to posterior, its colour turning progressively from brown-red to dark brownish; lateral lobe of mesoscutum with a large orange rectangular band on the antero-lateral corner continuing as a smaller grey band; axilla with an orange band at apex; lateral and ventral sides entirely yellow except two blackish spots at wing insertion; scutellum with a large triangular dark-brownish band, covering about half of the surface until reaching the frenum; frenal area nearly completely dark brownish, with a narrow yellow band on sides; lateral panel of metanotum with a narrow brownish band. Pilosity on thorax dark with long hairs on the yellow parts of pronotum, mid-lobe of mesoscutum (eight pairs of long hairs), lateral lobe of mesoscutum (three rows of hairs of different sizes), and scutellum (four lateral pairs, one on them on the frenum). Legs yellow except claws brown; fore femora with several long black hairs at apex, hind femora with numerous pale hairs; tibiae with 3 rows of long black hairs. Forewing stigma brown, without infuscation; basal cell closure poorly defined, with 9 small setae on disc; only 1 seta on basal setal line; 4 small setae on costal line; costal cell with 4 large setae (Figure 101). Propodeum with a large black rectangular band in its middle, and a brownish spot around spiracle. Gaster with a narrow, yellow longitudinal median band (Figure 96); T3 dark orange, T4 with a dark brown triangular spot extending from the median yellow line to the side of the tergite, continuing as an orange- brown band, thus delimiting a triangular yellow cell closed laterally by a comma- shaped blackish spot; lateral parts of gaster whitish. Ovipositor sheaths black, 1.8 × longer than gaster, 0.7 × as long as body (Figure 97).

Face quadrate, width: height ratio: 1.0 (Figure 99); POL: OOL 1.7; scrobe elongate, ca. 4.2× as long as wide. Scape 1.1 × as long as combined length of pedicel, anellus and F1 (Figure 100); scape 0.8× as long as combined length of pedicel, anellus, F1 and F2; anellus subquadrate; F1 1.2 × longer than pedicel, 1.7 × as long as wide; F2 1.9 × as long as wide; following funicular segments progressively tending to subquadrate, with F7 1.2 × as long as wide. Pronotum, mid- and lateral lobes of mesoscutum, and axilla with coarse transverse cross- striae. Mid-lobe of mesoscutum 1.5 × as long as scutellum length. Scutellum 0.9 × as long as wide, with reticulate striation; frenum 0.4 × as long as scutellum, with reticulate striation in the middle, and carinae laterally. Stigma oval- elongate, about 1.5 × as long as wide (Figure 101); upper part of stigmal vein elongate, 0.5 × as long as stigma length; uncus 0.7 × as long as upper part of stigmal vein; marginal vein 0.9× as long as postmarginal vein. Propodeum with coarse transverse striae.

**Variation.** Length of female body varies from 4.8 to 5.4 mm. The relative length of the ovipositor sheaths varies between 1.6 and 1.8 × the gaster length, and between 0.6 and 0.7 × the body length. In three out of the eleven specimens examined, the brownish bands on thorax and abdomen are closer to to dark orange, becoming less distinct. One specimen, with two setae on the basal setal line, a second specimen with four setae.

**Males.** Like in most other *Megastigmus* species related to Anacardiaceae, males are highly variable in colour, and two extreme forms can be distinguished with intermediates.

*Pale form*. Body length from 4.2 to 6.2 mm. Body colour mostly orange-yellow (Figures 102–104). Head orange with a blackish ring around occiput. Pilosity on lower face pale but conspicuous black hairs on parascrobal area, temple, gena and dorsum of head. Antenna yellow. Thorax orange-yellow except a small black spot at wing insertion and a more or less distinct median longitudinal band of darker orange colour extending such as in female from anterior suture of pronotum to scutellum. Pilosity on thorax black with 4 pairs of long setae on scutellum (Figure 104). Legs entirely yellow; pilosity pale on femurs, black on tibiae. Forewing stigma brown without infuscation (Figure 108); basal cell partly closed, with 2 long setae on disc; basal setal line with 2 long setae, costal setal line quite indistinct with 1 setae. Propodeum orange with a median black band; hairs on callus pale. Gaster colour mostly brown- yellow; T3 petiolate, black at insertion and then turning to brownish; T4 yellow; T5 with a transverse brown band on anterior suture; T6 with a small triangular brown spot on the middle; T7 with a larger triangular brown spot on the middle, and a small rounded brown spot on each side; T8 with a small rounded brown spot on each side. Long black hairs on tergite sutures. Genitalia with elongate aedeagus, its part above digitus about 1.8 × as long as digitus length; digitus with 3 teeth; phallobase elongate, about 2.8× as long as wide (Figure 107).

Head about 1.1 × as wide as long in front view (Figure 105). POL: OOL: 1.2. Scape 1.3 × as long as combined length of pedicel, anellus, and F1 (Figure 106); scape 0.9 × as long as combined length of pedicel, anellus, F1 and F2; F1 1.4 × as long as pedicel, 2.1 × as long as wide; F1 1.2 × as long as F2; following funicular segments elongate, not tending to subquadrate; F7 2.0× as long as wide. Pronotum with coarse transverse carinae, mid-lobe of mesoscutum and axilla with transverse striae finer than on pronotum (Figure 104). Mid-lobe of mesoscutum 1.2 × as long as scutellum length; Scutellum 1.3 × as long as wide, noticeably covered with irregular, arching, concentric wrinkles; frenum nearly indistinct from scutellum. Forewing stigma oblique, rounded, about 1.2 × as long as wide (Figure 108); upper part of stigmal vein 0.4 × as long as stigma length; uncus very short, 0.4 × as long as upper part of stigmal vein.

*Dark form*. Larger than pale form, with body length 5.7 to 6.4 mm. Body colour substantially black (Figures 109–112) with a conspicuous, large triangular infuscation around the forewing stigma (Figure 114). Head mostly black except a transverse dirty yellow band on the lower face (restricted to gena in some specimens) and a dirty yellow spot between eye and posterior ocellus (Figure 112). Thoracic dorsum dirty yellow with a large median black band extending from the anterior suture of pronotum to frenum (Figure 111). Sides of thorax entirely black (Figure 110). Legs mostly dark; coxae black; fore femora brownish in its basal part, mid- and hind femora quite entirely brownish; tibiae and tarsi yellow. Propodeum entirely black. Antenna (Figure 113), thoracic sculpture (Figure 111), pilosity and genitalia (Figure 115) similar to pale form.

*Intermediates*. Some specimens show a slightly infuscated stigma along with head and thorax mostly orange but with more blackish patterns than in the pale form: a black-brown band between eye margin and torulus, a brownish median band on thorax from anterior suture of pronotum to posterior suture of mid lobe of mesoscutum which prolongates in a fuzzy median band on scutellum, and a brown longitudinal band on upper and lower part of mesepimeron. Legs yellow with a large black spot on fore coxa, a smaller spot on mid-coxa, and a brownish infuscation on hind coxa. Propodeum with a large median black band, black spots around spiracles, and a brownish infuscation at suture with callus. Pilosity is similar to the one of pale from but some individuals have 3 pairs of hairs on scutellum.

#### Host plants.

*Lannea rivae* (Anacardiaceae). Probably a seed feeder. Although the presence of numts was noted in the COI sequences of the only specimen of *Megastigmus lanneae* which amplified for DNA analysis, this species clearly clustered within the “Anacardiaceae clade” (Figure 14) as already noticed for *Megastigmus ozoroae*.

#### Distribution.

Known only from farmland tree in Machakos area. Adults emerged from 3.3% of the collected fruits (Table 3).

#### Etymology.

Named after the genus of its host plant.

#### Diagnosis.

Females can be separated easily from those of other species associated with fruits of Anacardiaceae by the combination of an ovipositor at least 1.8 × as long as gaster length and a yellow and brownish body. In other species having dark and yellow patterns, the ovipositor length is either shorter than gaster length (*Megastigmus ozoroae*- Figure 43, *Megastigmus smithi*- Figure 31) or at most 1.5 × longer (*Megastigmus hypogeus*- Figure 117). Body colour is predominantly orange in *Megastigmus laventhali*, *Megastigmus pistaciae*, *Megastigmus thomseni* and *Megastigmus transvaalensis*. Genitalic characters allow separation of *Megastigmus lanneae* males from those of other species developing in seeds of Anacardiaceae. The aedeagus part above digitus is significantly longer (1.8 × as long as digitus length) than in *Megastigmus hypogeus* (1.2–1.3×; Figures 127, 134), *Megastigmus pistaciae* (0.8×; Figure 148) and *Megastigmus transvaalensis* (0. 5×; Figure 162). The aedaegus dimension compared to digitus is similar in *Megastigmus ozoroae* but the phallobase of *Megastigmus lanneae* is more elongate (2.8× vs. 2.6× longer than wide; Figures 53, 60).

### Megastigmus hypogeus

Taxon classificationAnimaliaHymenopteraTorymidae

(Hussey, 1956)

#### Type material examined.

**Holotype** ♀ “Bred ex. oil seed, Nairobi; 5–37; Kenya, Corydon Museum; Pres. by Com. Inst. Ent., B.M. 1957–41; B.M. TYPE HYM. 5.1653; NHMUK010263947” (BNHM); **Paratypes**: 3♀♀, 4♂♂, same collection data as holotype (BNHM);

#### Additional material.

11♀♀, 14♂♂, Kenya, Coast Province, Shimba Hills, 4°08.096'S, 39°28.082'E, 285m elevation, 25 Nov. 2001, ex. *Ozoroa obovata* seeds A&M coll. N°1609, R. Copeland leg.; 1♀, Nyanza Province, Sindo-Mbita Road, 0°29.091'S, 34°10.592'E alt 1205m, 11 Dec 2004, ex. *Ozoroa insignis* ssp *reticulata* seeds, A&M coll. N°3064, R. Copeland leg.; 11♀♀, 8♂♂, Arabuko-Sokoke forest, 3.3716°S, 39.8949°E, 55m elevation, 18 Feb 2000., coll. N°Kip-372, ex. *Ozoroa obovata* seeds, R. Copeland leg; 2♀♀, 3♂♂, Arabuko-Sokoke forest, 3.3716°S, 39.8949°E, 55m elevation, 18 Feb 2000, ex. *Ozoroa obovata* seeds, A&M coll. N°509, R. Copeland leg.; 1♀, Arabuko-Sokoke forest, 3.2030°S, 39.9271°E, 98m elevation, 17 Mar 2001, ex. *Ozoroa obovata* seeds, A&M coll. N°1103, R. Copeland leg. (all material in RSC except 5♀♀, 5♂♂ from Arabuko-Sokoke forest in ARC)

This species has been first described by Hussey (1956a) under the name of *Eumegastigmus hypogea*, and erroneously reported to emerge from”Oil seed, probably *Helianthus* sp.” in Nairobi, Kenya. No subsequent records have been published since this original description. In his paper, Hussey mentioned that it is a very variable species. Our collection of a large number of specimens from different hosts, all belonging to the Anacardiaceae genus *Ozoroa*, allowed us to supplement Hussey’s primary description which was rather limited.

**Female**. Females range in length from 3.9 to 5.1 mm (without ovipositor), length of ovipositor sheaths from 2.1 to 2.5 mm. Body colour usually yellow and black (Figures 116–118) but some individuals present a dominant orange colour, all the blackish patterns becoming orange-brown. Head yellow with upper part of scrobe black (but interocellar area yellow), and vertex with a brownish infuscation. Pilosity on face, gena and temples pale but with a few long black hairs near clypeus and parascrobal area (Figure 119); vertex with long black hairs. Antenna brownish.

Thorax yellowish-brown with blackish patterns (Figures 116, 118). Pronotum with a large, median dark brown band progressively decreasing in width from anterior to posterior suture (Figure 118) but in a few specimens, the median pronotum line is very light and interrupted at its distal end; mid-lobe of mesoscutum completely black at the anterior part, continuing as a large triangular brown- black band progressively narrowing from anterior to posterior, the sides yellow; lateral lobe of mesoscutum yellow with an orange rectangular band becoming grey posteriorly, continuing on axilla as a similar orange band; a brownish longitudinal line on lower mesepimeron; scutellum with a triangular blackish band increasing in size from the anterior suture to frenal area (but sometimes reduced to a narrow line with an orange-brown infuscation); conspicuous black sutures between the following parts: lateral panel of pronotum and collar, mid-lobe of mesoscutum and lateral lobe of mesoscutum, lateral lobe of mesoscutum and prepectus, axilla and scutellum, callus and metapleuron. In the individuals with a dominant orange colour, the sutures between lateral lobe of mesoscutum and prepectus, wing insertion and middle part of propodeum remain black. Pilosity on thorax with long black hairs; mid-lobe of mesoscutum with five lateral pairs of hairs on the yellow parts; scutellum with 3–4 lateral pairs of hairs on the yellow sides (4 pairs on holotype; 3 pairs on most other specimens). Legs yellow except claws brown; pale hairs on coxa, with conspicuous blackish insertion dots; femora with both pale and black hairs; hairs black at apex of femora; 3 rows of long black hairs on tibia. Forewing stigma brown without infuscation; basal cell indistinct, basal setal line with only 1 seta; costal cell with 3 setae (Figure 121). Propodeum light brown with a large, rectangular longitudinal black band medially. Gaster mostly brown with a narrow yellow line in the middle; T3 black; subsequent terga with transverse brown bands on the dorsum, narrowing progressively towards the lateral sides which are whitish; gaster completely brown on some specimens with only a few whitish dots on sides; in a few others, gaster has an orange T3, with the subsequent tergites having an orange longitudinal band anteriorly, the band becoming yellow posteriorly. The brownish lines on the lateral sides of gaster segments are sometimes absent. Ovipositor sheaths black, Ovipositor sheaths 1.2 × longer than gaster, 0.6 × as long as body (Figure 117).

Face subquadrate, width: height ratio in front view: 1.2×; POL: OOL 1.6×; inter-antennal area as broad as torulus width; scrobe rather short, ca. 2.9 × as long as wide. Scape 1.3 × as long as combined length of pedicel, anellus, and F1 (Figure 120); scape 0.8 × as long as combined length of pedicel, anellus, F1 and F2; anellus subquadrate; F1 1.3 × as long as pedicel, 2.1 × as long as wide; F2 2.1× as long as wide; following funicular segments elongate tending to subquadrate only from F6, with F7 1.3 × as long as wide. Pronotum with coarse cross- striae, mid- and lateral lobes of mesoscutum, and axilla with finer strong cross- striae (Figure 118). Mid-lobe of mesoscutum 1.4 × as long as scutellum length. Scutellum 1.4 × as long as wide, reticulate; frenum indistinct, 0.4 × as long as scutellum, reticulate in the middle, and with smooth striae on sides. Stigma rectangular, elongate, about 1.9 × as long as wide (Figure 121); upper part of stigmal vein elongate, 0.4 × as long as stigma length; uncus 0.8 × as long as upper part of stigmal vein; marginal vein 0.9 × as long as postmarginal vein. Propodeum with cross- striae becoming reticulate anteriorly, a very weak median carina posteriorly.

**Males.** They are highly variable in colour, and two extreme forms can be distinguished with intermediates. Paratypes ♂ include three specimens with pale patterns and one with dark patterns whereas our collection included nine specimens of pale form, 13 of dark form, and three of intermediate color.

*Pale form*. Body length from 4.3 to 5.4 mm. Body colour mostly orange-yellow (Figures 122–124). Head yellow with a black spot covering the interantennal area and the scrobes (Figure 125), extending in a semi-circle to the anterior ocellus, then lengthening in a median irregular line until pronotum insertion. Pilosity on lower face pale, black on the remainder of head; parascrobal area with a line of small setae along the scrobes, and a line of long black bristles along the eye contour; two small setae in the interocellar area; two rows of bristles around pronotum insertion. Antenna yellow.

Thorax mostly yellow- orange with a few black patterns (Figure 124). Pronotum yellow with a triangular black spot (very light in a few specimens) in the middle of pronotum, enlarging apically but not reaching the mid lobe of mesoscutum, and a fuzzy blackish band along the posterior suture; mid lobe of mesoscutum orange with a narrow, median black band extending from anterior to posterior suture (very light in one of the ♂ paratypes); lateral lobes of mesoscutum yellow; axilla yellow with a transparent, rectangular band on the antero-lateral part; scutellum yellow with the anterior suture black prolongating in its middle by a small fuzzy blackish spot surrounded by an orange spot. Posterior sutures of metanotum black. Pilosity on thoracic dorsum black but pale on mesepisternum, mesepimeron and callus; 4 pairs of long black bristles on scutellum (Figure 124). Legs yellow. Forewing stigma of paratype brown with a slight yellowish contour but the specimens we collected did not show such a contour (Figure 128); basal cell only partly closed, the disc with 6 setae; basal line and costal line each with 2 setae; costal cell more than 20 small hairs in 4–5 rows in the apical 1/2 to 2/3. Propodeum yellow with a black, median patch of rombhoid shape, extending from anterior to posterior part; sutures with callus blackish. Gaster petiolate, mostly black with few yellow patterns (Figure 122); T3 elongate, entirely black on dorsum; T4 with a large transvers blackish band on the anterior part and a narrower yellow band on the posterior part, extending on sides; T5 similar; T6 and T7 with the yellow band limited to the sides; last segments yellow. Gaster entirely black on dorsum in a few specimens. A row of long black bristles on the lateral parts of tergites 5, 6, 7 and numerous bristles on T8.

Face rounded, width: height ratio in front view: 1.0× (Figure 125); POL: OOL 1.4×. Scape 1.2 × as long as combined length of pedicel, anellus, and F1 (Figure 126); scape 0.9 × as long as combined length of pedicel, anellus, F1 and F2; anellus subquadrate; F1 elongate, 1.5 × as long as pedicel, 2.1 × as long as wide; F2 and following funicular segments similarly elongate, with F7 1.4 × as long as wide.. Pronotum with strong transverse cross-striae; mid lobe of mesoscutum with finer, very irregular cross-striae (Figure 124); mid-lobe of mesoscutum 1.1 × as long as scutellum length; scutellum 1.2 × as long as wide, irregularly reticulate; frenum 0.4 × as long as scutellum length, reticulate as scutellum in its middle but with longitudinal carinae on the sides. Forewing stigma nearly rectangular, 1.4 × as long as wide (Figure 128); upper part of stigmal vein short, 0.2 × as long as stigma length; uncus short, 0.6 × as long as upper part of stigmal vein; marginal vein 0.8 × as long as postmarginal vein. Propodeum with coarse, irregular carinae extending laterally but no median carina. Genitalia with short aedeagus, its part above digitus about 1.2 × as long as digitus length; digitus compressed, about 2.3 × longer than its maximum width, with three teeth (Figure 127).

*Dark form*. Body length slightly longer than in pale form, from 5.0 to 5.5 mm. Body substantially black (Figures 129–132). Head entirely black (Figure 132) but antenna yellow with scape brownish (Figure 133). Pronotum dark-yellowish with a large median black extending from head insertion to mid lobe of mesososcutum, a large black rectangular patch on the sides, and a larger fuzzy blackish band along the posterior suture (Figure 131); mid lobe of mesoscutum with a large median, dark orange brown band covering most of the surface; lateral lobes of mesoscutum and axilla dark orange; scutellum with a large brown- black median band from anterior to posterior suture. Lateral parts of the thorax (mesepisternum, mesepimeron and callus) entirely black except acropleuron dark orange. Legs mostly black; coxae black; femora black on the apical 2/3 in fore- and mid- femur, and quite completely in hind femur; tibiae and tarsi yellow. Forewing stigma brown with a large, triangular infuscation (Figure 135); basal cell with 16 strong setae on disc; basal line with 3 strong setae; costal line with 26 small setae; costal cell with more than 100 small hairs in a number of rows. Pilosity of thorax similar to that in pale form. Propodeum entirely black (Figure 131). Gaster conspicuously petiolate, entirely black (Figure 129–130).

Relative dimensions of head and antenna similar as in pale form; sculpture of thorax and propodeum same as in pale form. Forewing stigma nearly rectangular, 1.3 × as long as wide (Figure 135); upper part of stigmal vein very short, only 0.2 × as long as stigma length; uncus as long as upper part of stigmal vein; marginal vein 0.8 × as long as postmarginal vein. Aedeagus similar to pale form but one digitus with four teeth, the other one with three teeth (Figure 134).

#### Intermediates.

Body length as in pale form, from 4.6 to 5.4 mm. Some specimens with infuscate stigma show lighter coloration patterns than in the extreme dark form, with the median band on pronotum smaller, the lateral lobes of mesoscutum and axilla yellow, the scutellum with the median band limited to the anterior part, and the gaster with narrow yellow bands from T4 to T6.

#### Host plants.

*Ozoroa obovata*, *Ozoroa insignis* ssp *reticulata* (Anacardiaceae). Probably a seed feeder, based on its place in the molecular phylogeny of *Megastigmus* spp. where it clusters with the other species developing in seeds of Anacardiaceae (Figure 14), such as *Megastigmus ozoroae* and *Megastigmus lanneae*, and despite the presence of numts in the short COI fragment.

#### Distribution.

Mixed bush-/grassland in western Kenya near Lake Victoria and in similar habitat in coastal areas. Adults emerged from up to 2.8% of the fruits in some collections (Table 3).

#### Diagnosis.

The key from Hussey (1956a) distinguished the females of *Megastigmus hypogeus* by their large body (>5 mm), the ocelli surrounded with black, and the thoracic dorsum with longitudinal black stripe. Actually, it does not allow one to separate females of this species from those of some other species associated with fruits of Anacardiaceae, which are also large and present a thoracic dorsum with a longitudinal dark band; i.e., *Megastigmus lanneae*, *Megastigmus ozoroae*, and *Megastigmus smithi. Megastigmus hypogeus* differs from these species by the relative length of its ovipositor being 1.2–1.3 × longer than gaster length whereas it is shorter than gaster length in *Megastigmus ozoroae* (Figure 43), and *Megastigmus smithi* (Figure 31), and much longer in *Megastigmus lanneae* (1.8×; Figure 97).

Genitalic characters also allow separation of *Megastigmus hypogeus* males from those of other species developing in seeds of Anacardiaceae, the aedeagus part above digitus being significantly less elongated (1.2 × as long as digitus length) than in *Megastigmus ozoroae* (1.8×; Figures 53, 60) and *Megastigmus lanneae* (1.8×; Figures 107, 115) but much longer than in *Megastigmus pistaciae* (0.8×; Figure 148) and *Megastigmus transvaalensis* (0. 5×; Figure 162).

### Megastigmus pistaciae

Taxon classificationAnimaliaHymenopteraTorymidae

Walker, 1871

#### Material examined.

3♀♀, 3♂♂, Kenya, Mount Kulal, 2.6290°S, 36.9278°E, 1640m elevation, 11 Dec 2008, ex. *Pistacia lentiscus* subsp. *emarginata* seeds, R. Copeland leg. (2♀♀, 1♂ RSC; 1♀, 1♂ ARC; 1♂ destroyed)

Male and female specimens fit the detailed description of the species by Roques and Skrzypczyńska (2003) as shown by figures 136–141 presenting the most important characteristics of female specimens from Kenya. Similarly as in most *Megastigmus* species developing in Anacardiaceae seeds in Kenya, males are highly variable ranging from pale forms, mostly yellow with forewing stigma without infuscation (Figures 142–144 and 146–149,) to dark forms, mostly black with infuscated stigma (Figure 145). However, the forewing description of female *Megastigmus pistaciae* used by Grissell and Prinsloo (2001) as a diagnostic character in the key to separate the species of *Megastigmus* reared from seeds of Anacardiaceae appears largely different from our observations. To separate it from female *Megastigmus thomseni*, Grissell and Prinsloo (2001) stated that the costal cell of the forewing of *Megastigmus pistaciae* presents 3 or 4 rows of setae in the apical 1/2 to 2/3 and the basal cell more than 5 setae whereas there is at most a single row of setae in the costal cell and at most 3–4 setae in the basal cell of *Megastigmus thomseni*. Actually, all the specimens of *Megastigmus pistaciae* we examined from Kenya, but also from other parts of the world (Europe, North Africa, California) did not show the characters mentioned by Grissell & Prinsloo but were closer to those attributed to the forewing of *Megastigmus thomseni*. Since these authors did not mention the origin of the specimens they examined, it is difficult to evaluate their observations.

In the molecular phylogeny of *Megastigmus*, the Kenyan specimen clusters with the other specimen of *Megastigmus pistaciae* from Southern Europe (Figure 14), confirming their status of sister species belonging to the same ”Anacardiaceae clade”. However, there was a genetic distance of 4.5% between these specimens and according to Auger-Rozenberg et al (2006) and Scheffer and Grissell (2003), clearly differentiated species of *Megastigmus* diverge by more than 4.0%. This suggests an opportunity to develop further studies to evaluate the possibility that the Kenyan populations represent a sibling species of *Megastigmus pistaceae*.

#### Host plants.

*Pistacia lentiscus* subsp. *emarginata* seeds.

#### Distribution.

Reared from samples collected in highland areas (1640–1920 m above sea level) of the Rift Valley, in northern and southwestern Kenya. Few fruits (0.6%) were infested (Table 3).

#### Diagnosis.

Females can be separated from those of other species with a predominantly orange colour associated with fruits of Anacardiaceae by the shape of the forewing stigma and the relative length of ovipositor. The stigma is oval in *Megastigmus pistaciae* (1.2–1.3× as long as wide; Figure 141) whereas it is much more elongate in both *Megastigmus transvaalensis* (2.0×; Figure 155) and *Megastigmus thomseni* (1.7×, according to the drawing by Hussey 1956a). The ovipositor is only a bit longer than gaster length (Figure 137) whereas it is nearly twice as long as gaster in *Megastigmus laventhali* (1.8×; Figure 76) and 1.4 × as long as gaster length in *Megastigmus transvaalensis* (Figure 151).

The genitalia allow one to separate males from those of other species identified in seeds of Anacardiaceae. The aedeagus part above digitus is comparatively longer than in *Megastigmus transvaalensis* (0.8 × vs. 0.5 × as long as digitus length; Figures 148 and 162, respectively) whereas this part is much more elongated in *Megastigmus hypogeus* (1.2 × as long as digitus length; Figures 127, 134), *Megastigmus ozoroae* (1.8×; Figures 53, 60) and *Megastigmus lanneae* (1.8×; Figures 107, 115).

### Megastigmus transvaalensis

Taxon classificationAnimaliaHymenopteraTorymidae

(Hussey, 1956)

#### Material examined.

3♀♀, 7♂♂, Kenya, Kikuyu Escarpment, Central Province, 1.0290°S, 36.6025°E, 2100 m, coll. 85, ex. *Rhus vulgaris* seeds, 29 Apr 1999, R. Copeland leg.; 2♀♀, 3♂♂, Kenya, Burguret, Central Province, 0.1128°S, 37.0375°E, coll. 2162, ex. *Rhus natalensis* seeds, 16 Aug 2002, R. Copeland leg.; 3♀♀, 7♂♂, Kenya, Nairobi Province, 1.2212°S, 36.8963°E, 1610m, coll. 2787, ex. *Schinus terebinthifolius* seeds, 28 Apr 2004, R. Copeland leg. (4♀♀, 10♂♂ RSC; 4♀♀, 7♂♂ ARC)

Male and female specimens fit the description of the species by Hussey (1956a) and Grissell and Prinsloo (2001), then detailed by Roques and Skrzypczyńska (2003). Figures 150–155 present the most important characteristics of female specimens from Kenya whereas Figures 156–162 show those of males.

#### Host plants.

*Schinus molle*, *Schinus terebinthifolius*, *Rhus natalensis*, *Rhus vulgaris* (Anacardiaceae). A seed feeder. Schefer and Grissell (2003) presented a molecular analysis of the populations of this species, suggesting its origin in Austral Africa but we were unable to amplify any specimens of the Kenyan populations we obtained.

#### Distribution.

Adults emerged from 14.8% of the fruits of *Schinus molle*, 14.7% of those of *Rhus vulgaris* and up to 6.1% of *Rhus natalensis* fruits (Table 3).

#### Diagnosis.

Females can be distinguished from these of other species associated with fruits of Anacardiaceae and showing a predominantly orange body by the relative size of the ovipositor and the shape of the forewing stigma. In *Megastigmus transvaalensis*, the ovipositor is 1.4 × as long as gaster length (Figure 151) whereas it is nearly twice as long as the gaster in *Megastigmus laventhali* (1.8×; Figure 76) and only a bit longer than gaster in *Megastigmus thomseni* and *Megastigmus pistaciae* (1.1–1.2×; Figure 137). The stigma is conspicuously elongate, and quite rectangular in *Megastigmus transvaalensis* (2.0 × as long as wide; Figure 155), less elongate in *Megastigmus thomseni* (1.7×, according to the drawing by Hussey 1956a) whereas it is oval and rounded in *Megastigmus pistaciae* (1.2–1.3×; Figure 141).

Genitalia allows the separation of males from those of other species reared from seeds of Anacardiaceae. The aedeagus part above digitus is conspicuously shorter than digitus length (0. 5×; Figure 162) whereas this part is more elongated in *Megastigmus pistaciae* (0.8×; Figure 148), *Megastigmus hypogeus* (1.2 × as long as digitus length; Figures 127, 134), *Megastigmus ozoroae* (1.8×; Figures 53, 60) and *Megastigmus lanneae* (1.8×; Figures 107, 115).

### Megastigmus somaliensis

Taxon classificationAnimaliaHymenopteraTorymidae

Hussey, 1955

#### Type material examined.

Holotype ♀ “Bristish Somaliland; ex. seeds of *Juniperus procera*; coll. i.1954, em. 25.ii.1954, J.T. Lawrie; Brit. Mus. 1956–294; Com. Inst. Ent. coll. 13661; B.M. TYPE HYM. 5.1623a; NHMUK010263946 5.1623a”(BNHM). Paratypes 4♀♀, 2♂♂, same collection data as holotype (BNHM);

#### Additional material.

1♀ “Abyssinia, Mulu, above Muger Valley; circa 8000 ft., 18–23. 12. 1926, Dr. H. Scott” (BNHM); 1♀ “Abyssinia, Mt. Chillálo Digula; circa 9500 ft., 27.11.1926, from foliage giant juniper, Dr. H. Scott” (BNHM); 3♀♀, 2♂♂, Kenya, Central Prov., Mt. Kenya Forest, 0.0308°S, 37.1230°E, 2125m, coll. 3034, ex. *Juniperus procera* seeds, 2 Nov. 2004, R. Copeland leg. (2♀♀, 1♂, RSC; 1♀ ARC; 1♂ destroyed).

Male and female specimens obtained from Kenya fit the general description of the species by Hussey (1956b). Figure 163 presents a view of female body whereas Figures 164–167 show the major morphological characteristics of male specimens from Kenya. Body colour is dark orange in the Kenyan specimens instead of pale orange in the type material from former British Somaliland, at present Somalia. However, at least one dark orange female was noticed in the BNHM collection, which had been collected in Digula, Abyssinia, at present Ethiopia.

#### Host plants.

*Juniperus procera*. A seed feeder clustering in the molecular phylogeny of *Megastigmus* with the other species developing in seeds of *Juniperus* in Europe (Figure 14). This species clearly belongs with strong support to the “Cupressaceae clade” whatever the marker and the analysis. It is close to *Megastigmus amicorum*, a seed chalcid which is widely distributed on *Juniperus* from the Mediterranean basin. The host of *Megastigmus somaliensis*, *Juniperus procera*, is the only juniper out of more than 60 species growing in the Arabian Peninsula and in Africa (Mao et al. 2010). It suggests that the host and the associated chalcid originated from regions more northern than the ones where they are presently observed, which could explain the phylogenetic proximity with other juniper seed chalcids.

#### Distribution.

Sampled in highland forest and woodland in central and northwestern Kenya (Figure 1). Also present in Somalia and Ethiopia.

#### Diagnosis.

This species, as well as the related *Megastigmus asir* which also attacks *Juniperus procera* (Ghram and Shati 2011), are easily differentiated from the other Afrotropical *Megastigmus* by the 7–8 pairs of hairs on the lateral parts of scutellum (Figure 166) whereas at most 5 pairs are present in the other species. Females of *Megastigmus somaliensis* are distinguished from those of *Megastigmus asir* by the shape of the forewing stigma and the sculpture of the propodeum. In the first species, forewing stigma is oval (1.6 × as long as wide measured from the drawing of Hussey 1956b) whereas it is much more elongate *Megastigmus asir* (2.2 × as long as wide when measured on the corresponding wing photo in Ghram and Shati (2011), although the authors mentioned in their text this value to be 1.5×). Propodeum is rugose and irregularly striated in *Megastigmus somaliensis* whereas it is smooth in *Megastigmus asir* (Ghram and Shati 2011). The propodeum of males shows the same differences, the one of *Megastigmus somaliensis* additionally presenting a very short median carina in its anterior part (Figure 166). Forewing stigma is quite similar in the two species (1.4 × as long as wide in Megastigmus somaliensis- Figure 167- vs. 1.35 × in *Megastigmus asir*; Ghram and Shati 2011).

## Conclusion

Is the radiation on Angiosperms more important than previously considered?

Our results increase to 16 the number of *Megastigmus* species presently known from the Afrotropical region, of which at least 13 are seed feeders. The results also increase to 28 the number of species shown to be associated worldwide with angiosperm seeds vs. 48 with conifers, and to 7 the number of angiosperm families hosting *Megastigmus* seed chalcids (Figure 168). These angiosperm families are rather distant taxonomically, belonging to different clades and orders within the new, molecular-based classification system of angiosperms (APG III; The Angiosperm Phylogeny Group 2009): Rosaceae and Rhamnaceae in order Rosales and Fabaceae in order Fabales of the clade Fabids; Anacardiaceae in order Sapindales and Malvaceae in order Malvales of the clade Malvids; Hamamelidaceae in order Saxifragales of the clade Core eudicots; and Aquifoliaceae in order Aquifoliales of the clade Campanulids. Rosaceae still host the largest number of chalcid species but there is increasing evidence of a large radiation in Anacardiaceae. Thus, *Megastigmus* seed chalcids appear to have radiated in Angiosperms much more than previously considered, and it is likely that new host genera, and possibly new host families are to be discovered. The combination of morphological with molecular evidence provides a more robust method for analyzing relationships between chalcid species and their host plants. In the future, such a combined analysis could also help to reliably link the different male colour forms with conspecific females.

## Supplementary Material

XML Treatment for Megastigmus helinae

XML Treatment for Megastigmus smithi

XML Treatment for Megastigmus copelandi

XML Treatment for Megastigmus ozoroae

XML Treatment for Megastigmus icipeensis

XML Treatment for Megastigmus laventhali

XML Treatment for Megastigmus grewianae

XML Treatment for Megastigmus lanneae

XML Treatment for Megastigmus hypogeus

XML Treatment for Megastigmus pistaciae

XML Treatment for Megastigmus transvaalensis

XML Treatment for Megastigmus somaliensis

## Supplementary materials

Supplementary material 1 Sampling details on specimens used in the genetic study Data type: Collection data Explanation note: Collection data for the specimens used in the genetic study:

- 25 species already sequenced and used in a previous phylogenetic study (Boivin et al, 2014). (Note that *Torymus azureus* was used as an outgroup for building the phylogenetic trees of the *Megastigmus* genus).
- 4 species already described in the literature but sequenced and analysed for this paper.
- 5 new species described in this paper.

This dataset is made available under the Open Database License (http://opendatacommons.org/licenses/odbl/1.0/). The Open Database License (ODbL) is a license agreement intended to allow users to freely share, modify, and use this Dataset while maintaining this same freedom for others, provided that the original source and author(s) are credited.Alain Roques, Robert S. Copeland, Laurent Soldati, Olivier Denux, Marie-Anne Auger-Rozenberg

## References

[B1] Adamski D Copeland RS Miller SE Hebert PDN Darrow K Luke Q (2010) A Review of African Blastobasinae (Lepidoptera: Gelechioidea: Coleophoridae), with new taxa reared from native fruits in Kenya. Smithsonian Contributions to Zoology 630. Smithsonian Institution Scholarly Press, Washington D.C., 68 pp.

[B2] African Plant Database (2015) Conservatoire et Jardin botaniques & South African National Biodiversity Institute. http://www.ville-ge.ch/musinfo/bd/cjb/africa/recherche.php [accessed March 2015]

[B3] Aly SM (2014) Reliability of long vs short COI markers in identification of forensically important flies. Croatian Medical Journal 55: 19–26. doi: 10.3325/cmj.2014.55.19 24577823 10.3325/cmj.2014.55.19 PMC3944415

[B4] Angiosperm Phylogeny Group (2009) “An update of the Angiosperm Phylogeny Group classification for the orders and families of flowering plants: APG III”, Botanical Journal of the Linnean Society 161: 105–121. doi: 10.1111/j.1095-8339.2009.00996.x

[B5] Auger Rozenberg MA Kerdelhué C Magnoux E Turgeon J Rasplus JY Roques A (2006) Molecular phylogeny of conifer seed chalcids in the genus *Megastigmus* (Hymenoptera: Torymidae) and evolution of host-plant use. Systematic Entomology 31: 47–64. doi: 10.1111/j.1365-3113.2005.00310.x

[B6] Auger Rozenberg MA Roques A (2012) Seed wasp invasions promoted by unregulated seed trade affect vegetal and animal biodiversity. Integrative Zoology 7: 228–246. doi: 10.1111/j.1749-4877.2012.00308.x 22938521 10.1111/j.1749-4877.2012.00308.x

[B7] Boivin T Henri H Vavre F Gidoin C Veber P Candau JN Magnoux E Roques A Auger-Rozenberg MA (2014) Epidemiology of asexuality induced by the endosymbiotic *Wolbachia* across phytophagous wasp species: host plant specialization matters. Molecular Ecology 23: 2362–2375. doi: 10.1111/mec.12737 24673824 10.1111/mec.12737

[B8] Brown JW Copeland RS Aarvik T Miller SE Rosati ME Luke Q (2014) Host records for fruit-feeding Afrotropical Tortricidae (Lepidoptera). African Entomology 22: 343–376. doi: 10.4001/003.022.0225

[B9] Copeland RS (2006) Host fruit processing – purpose, tools and methodology. In: Ekesi S Billah MK (Eds) A Field Guide to the Management of Economically Important Tephritid Fruit Flies in Africa. ICIPE Science Press, Nairobi, F1–F11.

[B10] Copeland RS Luke Q Wharton RA (2009) Insects reared from wild fruits in Kenya. Journal of East African Natural History 98: 11–66. doi: 10.2982/028.098.0104

[B11] Copeland RS Wharton RA Luke Q De Meyer M (2002) Indigenous hosts of *Ceratitis capitata* (Diptera: Tephritidae) in Kenya. Annals of the Entomological Society of America 95: 672–694. doi: 10.1603/0013-8746(2002)095[0672:IHOCCD]2.0.CO;2

[B12] Copeland RS Wharton RA Luke Q De Meyer M Machera P Okumu M Zenz N Lux S (2006) Geographic distribution, host fruits, and parasitoids of the African fruit fly pests *Ceratitis anonae*, *Ceratitis cosyra*, *Ceratitis fasciventris* and *Ceratitis rosa* (Diptera: Tephritidae) in Kenya. Annals of the Entomological Society of America 99: 261–278. doi: 10.1603/0013-8746(2006)099[0261:GDHFAP]2.0.CO;2

[B13] Copeland RS White IM M. Okumu M Machera P Wharton RA (2004) Insects associated with fruits of the Oleaceae (Asteridae, Lamiales) in Kenya, with special reference to the Tephritidae (Diptera). Bishop Museum Bulletin in Entomology 12: 135–164.

[B14] DAISIE (2009) Handbook of alien species in Europe. Springer Science + Business Media B.V., 400 pp.

[B15] Darriba D Taboada GL Doallo R Posada D (2012) jModelTest 2: more models, new heuristics and parallel computing. Nature Methods 9: 772. doi: 10.1038/nmeth.2109 10.1038/nmeth.2109 PMC4594756 22847109

[B16] Doǧanlar M (2011) Review of Palearctic and Australian species of *Bootanomyia* Girault 1915 (Hymenoptera: Torymidae: Megastigminae), with descriptions of new species. Turkish Journal of Zoology 35: 123–157

[B17] Doǧanlar M (2015) Diagnosis of *Megastigmus* spp. (Hymenoptera: Torymidae) reared from galls of *Leptocybe invasa* Fisher & Lasalle, 2004, (Hymenoptera: Eulophidae) on *Eucalyptus* spp. (Myrtaceae), with description of a new species from South Africa. Entomofauna- Zeitschrift für Entomologie 36: 561–580.

[B18] Doǧanlar M Hassan E (2010) Review of Australian Species of *Megastigmus* Dalman, 1820 Hymenoptera: Torymidae) Associated with Eucalyptus, with Descriptions of New Species. Australian Journal of Basic and Applied Sciences 4: 5059–5120.

[B19] Doǧanlar M Zache B Wilcken CF (2013) A new species of *Megastigmus* Dalman 1820 (Hymenoptera: Torymidae: Megastigminae) from Brazil. Florida Entomologist 96: 197–198. doi: 10.1653/024.096.0126

[B20] Essl F Dullinger S Rabitsch W Hulme PE Hülber K Jarošík V Kleinbauer I Krausmann F Kühn I Nentwig W Vilà M Gen.esi P Gherardi F Desprez-Loustau ML Roques A Pyšek P (2011) Socioeconomic legacy yields an invasion debt. Proceedings of the National Academy of Sciences of USA 108: 203–207. doi: 10.1073/pnas.1011728108 10.1073/pnas.1011728108 PMC3017203 21173227

[B21] Galindo-González J Sánchez-Velásquez LR Montiel CR (2012) First report of *Megastigmus* sp. (Hymenoptera: Torymidae) damaging *Abies* spp. (Pinales: Pinaceae) seeds in Veracruz, Mexico. Entomological News 122: 79–84. doi: 10.3157/021.122.0111

[B22] Ghramh HA Shati AA (2011) Description of a new species of *Megastigmus* Dalman (Hymenoptera: Chalcidoidea: Torymidae) from Saudi Arabia. African Journal of Biotechnology 10: 4502–4506.

[B23] Grissel EE (1999) An annotated catalog of World Megastigminae (Hymenoptera: Chalcidoidea: Torymidae). Contributions of the American Entomological Institute 31: 1–92.

[B24] Grissel EE (2006) A new species of *Megastigmus* Dalman, 1820 (Hymenoptera: Torymidae), galling seed capsules of *Eucalyptus camaldulensis* Dehhardt (Myrtaceae) in South Africa and Australia. African Entomology 14: 87–94.

[B25] Grissell EE Prinsloo GL (2001) Seed-feeding species of *Megastigmus* Dalman 1820 (Hymenoptera: Torymidae) associated with Anacardiaceae. Journal of Hymenoptera Research 10: 271–279.

[B26] Guindon S Dufayard JF Lefort V Anisimova M Hordijk W Gascuel O (2010) New Algorithms and Methods to Estimate Maximum-Likelihood Phylogenies: Assessing the Performance of PhyML 3.0. Systematic Biology 59: 307–321. doi: 10.1093/sysbio/syq010 20525638 10.1093/sysbio/syq010

[B27] Hall TA (1999) BioEdit: a user-friendly biological sequence alignment editor and analysis program for Windows 95/98/NT. Nucleic Acids Symposium Series 41: 95–98

[B28] Hedlin AF Yates HO III Cibrian-Tovar D Ebel BH Koerber TW Merkel EP (1980) Cone and seed insects of North America conifers . Environment Canada/Canadian Forestry Service: Ottawa, US Forest Service: Washington and SARH: Mexico, 122 pp.

[B29] Hussey NW (1956a) A new species of *Megastigmus* (Hym.: Chalcidoidea) from British Somaliland. Bulletin of Entomological Research 47: 57–59. doi: 10.1017/S0007485300046502

[B30] Hussey NW (1956b) A new genus of African Megastigminae (Hymenoptera: Chalcidoidea). Proceedings of the Royal Entomological Society of London B 25: 157–162. doi: 10.1111/j.1365-3113.1956.tb01114.x

[B31] Křístek J Jančařík V Procházková Z Skrzypczyńska M Uroševič B (Eds) (1992) Škůdci semen šišek a plodů lesních dřevin. Brázda, Praha, 286 pp.

[B32] Liebhold AM Brockerhoff EG Garrett LJ Parke JL Britton KO (2012) Live plant imports: the major pathway for forest insect and pathogen invasions of the US. Frontiers in Ecology and the Environment 10: 135–143. doi: 10.1890/110198

[B33] Mao K Hao G Liu J Adams RP Milne RI (2010) Diversification and biogeography of *Juniperus* (Cupressaceae): variable diversification rates and multiple intercontinental dispersals. New Phytologist 188: 254–272. doi: 10.1111/j.1469-8137.2010.03351.x 20561210 10.1111/j.1469-8137.2010.03351.x

[B34] Mendel H (1982) Dimethyl hydantoin formaldehyde (D.M.H.F.), an alternative mountant for insect genitalia. Biology Curators Group Newsletter 3: .

[B35] Milliron HE (1949) Taxonomic and biological Investigations in the genus *Megastigmus* with particular reference to the taxonomy of the Nearctic species (Hymenoptera: Chalcidoidea; Callimomidae). American Midland Naturalist 41: 257–420. doi: 10.2307/2421720

[B36] Narendran TC Girish Kumar P Vastrad AS (2010) Two new species of *Megastigmus* Dalman (Hymenoptera: Torymidae) from India, with a revised key to Indian species. Records of the Zoological Survey of India 110: 1–6.

[B37] Noyes J (2015) The Natural History Museum. Universal Chalcidoidea Database. http://www.nhm.ac.uk/research-curation/research/projects/chalcidoids/database

[B38] Olmi M Copeland RS Guglielmino A (2015) An updated checklist of Dryinidae, Embolemidae and Sclerogibbidae (Hymenoptera) of Kenya and Burundi, with descriptions of thirteen new species. Acta Entomologica Musei Nationalis Pragae 55: 333–380

[B39] Razowski J Brown JW (2012) Descriptions of new Tortricidae (Lepidoptera) reared from native fruit in Kenya. Zootaxa 3222: 1–27.

[B40] Ronquist F Huelsenbeck JP (2003) MrBayes version 3.0: Bayesian phylogenetic inference under mixed models. Bioinformatics 19: 1572–1574. doi: 10.1093/bioinformatics/btg180 12912839 10.1093/bioinformatics/btg180

[B41] Roques A Kenis M Lees D Lopez-Vaamonde C Rabitsch W Rasplus JY Roy DB (Eds) (2010) Alien terrestrial arthropods of Europe. Pensoft, Sofia-Moscou, 1024 pp.

[B42] Roques A Skrzypczyńska M (2003) Seed-infesting chalcids of the genus *Megastigmus* Dalman (Hymenoptera: Torymidae) native and introduced to Europe: taxonomy, host specificity and distribution. Journal of Natural History 37: 127–238. doi: 10.1080/713834669

[B43] Roques A Sun JH Auger-Rozenberg MA Hua O (2003) Potential invasion of China by exotic insect pests associated with tree seeds. Biodiversity and Conservation 12: 2195–2210. doi: 10.1023/A:1024592716172

[B44] Scheffer SJ Grissell EE (2003) Tracing the geographical origin of *Megastigmus transvaalensis* (Hussey 1956) (Hymenoptera: Torymidae): an African wasp feeding on a South American plant in North America. Molecular Ecology 12: 415–421. doi: 10.1046/j.1365-294X.2003.01725.x 12535092 10.1046/j.1365-294x.2003.01725.x

[B45] Song H Buhay JE Whiting MF Crandall KA (2008) Many species in one: DNA barcoding overestimates the number of species when nuclear mitochondrial pseudogenes are coamplified. Proceedings of the National Academy of Sciences of USA 105: 13486–13491. doi: 10.1073/pnas.0803076105 10.1073/pnas.0803076105 PMC2527351 18757756

[B46] Sureshan PM (2009) Two species of *Megastigmus* Dalman associated with wild rose, *Rosa webbiana* (Rosaceae) from Ladakh, India with a key to Oriental species (Hymenoptera: Chalcidoidea: Torymidae). Journal of Threatened Taxa 1: 305–308. doi: 10.11609/JoTT.o2104.305-8

[B47] Tamura K Stecher G Peterson D Filipski A Kumar S (2013) MEGA6: Molecular Evolutionary Genetics Analysis Version 6.0. Molecular Biology and Evolution 30: 2725–2729. doi: 10.1093/molbev/mst197 24132122 10.1093/molbev/mst197 PMC3840312

[B48] Thompson JD Higgins DG Gibson TJ (1994) Clustal W: improving the sensitivity of progressive multiple sequence alignment through sequence weighting, positions-specific gap penalties and weight matrix choice. Nucleic Acids Research 22: 4673–4680. doi: 10.1093/nar/22.22.4673 7984417 10.1093/nar/22.22.4673 PMC308517

[B49] Townes H (1972) A light-weight Malaise trap. Entomological News 83: 239–247.

[B50] Tropicos (2015) Missouri Botanical Garden. http: //www.tropicos.org [accessed April 2015]

[B51] White IM Copeland RS Hancock DL (2003) Revision of the afrotropical genus *Trirhithrum* Bezzi (Diptera: Tephritidae, Ceratitidini). Cimbebasia 18: 71–137.

