# Corrigenda: Roques A, Copeland RS, Soldati L, Denux O, Auger-Rozenberg M-A (2016) *Megastigmus* seed chalcids (Hymenoptera, Torymidae) radiated much more on Angiosperms than previously considered. I- Description of 8 new species from Kenya, with a key to the females of Eastern and Southern Africa. ZooKeys 585: 51–124. doi: 10.3897/zookeys.585.7503

**DOI:** 10.3897/zookeys.620.10663

**Published:** 2016-09-29

**Authors:** Alain Roques, Robert S. Copeland, Laurent Soldati, Olivier Denux, Marie-Anne Auger-Rozenberg

**Affiliations:** 1 INRA, UR633, Zoologie Forestière, 2163 Avenue Pomme de Pin, F-45075, Orléans, France; 2 ICIPE, International Centre of Insect Physiology and Ecology, P.O. Box 30772, Nairobi 00100, Kenya; 3 National Museums of Kenya, Division of Invertebrate Zoology, P.O. Box 40658, Nairobi 00100, Kenya; 4 INRA, UMR 1062, Centre de Biologie pour la Gestion des Populations, Campus International de Baillarguet, CS 30016, F-34988, Montferrier-sur-Lez, France

On p. 81 the header for the new species reads *Megastigmus pistaciae* Roques & Copeland sp. n.

The CORRECT species name is *Megastigmus ozoroae* Roques & Copeland sp. n. The name was misspelled when the PDF file was generated.

