# Supplementary material for: Megastigmus seed chalcids (Hymenoptera, Torymidae) radiated much more on Angiosperms than previously considered. I- Description of 8 new species from Kenya, with a key to the females of Eastern and Southern Africa
Source: Zookeys. 2016 Apr 26;(585):51–124. doi: 10.3897/zookeys.585.7503 (PMC4857038; doi:10.3897/zookeys.585.7503)
Supplement: Supplementary material 1 — Sampling details on specimens used in the genetic study [file zookeys-585-051-s001.docx]

Appendix S1. Collection data for the specimens used in the genetic study :

- 25 species already sequenced and used in a previous phylogenetic study (Boivin et al, 2014). (Note that *Torymus azureus* was used as an outgroup for building the phylogenetic trees of the *Megastigmus* genus).

- 4 species already described in the literature but sequenced and analysed for this paper.

- 5 new species described in this paper.

| **Species name** | **Host-plant group** | **Host-plant family** | **Host-plant species** | **Collection site** |
| --- | --- | --- | --- | --- |
| *M. aculeatus* (Swederus) | Angiosperm | Rosaceae | *Rosa majalis* Herrm. | Krasnoyarsk, Russia |
| *M. aculeatus nigroflavus* Hoffmeyer | Angiosperm | Rosaceae | *Rosa multiflora* Thunb. | Dulles, USA |
| *M. alba* Roques | Angiosperm | Rosaceae | *Rosa* sp. | Mytilini, Greece |
| *M. amicorum* Bouček | Gymnosperm | Cupressaceae | *Juniperus phoenicea* L. | Baux-de-Provence, France |
| *M. atedius* Walker | Gymnosperm | Pinaceae | *Picea* sp. | Vernon, Canada |
| *M. atlanticus* Roques and Skrzypczyńska | Gymnosperm | Cupressaceae | *Cupressus atlantica* Gaussen | Idni, Morocco |
| *M. bipunctatus* (Swederus) | Gymnosperm | Cupressaceae | *Juniperus communis* L. | Briançon, France |
| *M. borriesi* Crosby | Gymnosperm | Pinaceae | *Abies koreana* Wils. | Rold skov, Denmark |
| *M. brevicaudis* Ratzeburg | Angiosperm | Rosaceae | *Sorbus* sp. | Ojcow, Poland |
| *M. cryptomeriae* Yano | Gymnosperm | Taxodiaceae | *Cryptomeria fortunei* Hooibr. | Hangzhou, China |
| *M. hoffmeyeri* Walley | Gymnosperm | Pinaceae | *Tsuga canadensis* (L.) Carrière | North Bay, Canada |
| *M. lasiocarpae* Crosby | Gymnosperm | Pinaceae | *Abies amabilis* (Dougl.) Forbes | Rutherford Creek, Canada |
| *M. nigrovariegatus* Ashmead | Angiosperm | Rosaceae | *Rosa* sp. | Victoria, Canada |
| *M. pictus* (Förster) | Gymnosperm | Pinaceae | *Larix decidua* Mill. | Krynica, Poland |
| *M. pinsapinis* Hoffmeyer | Gymnosperm | Pinaceae | *Cedrus atlantica* (Endl.) Manetti | Tala-guilef, Algeria |
| *M. pistaciae* Walker | Angiosperm | Anacardiaceae | *Pistacia terebinthus* Scop. | Exocori, Greece |
| *M. pinus* Parfitt | Gymnosperm | Pinaceae | *Abies grandis* Lindl. | Vernon, Canada |
| *M. rafni* Hoffmeyer | Gymnosperm | Pinaceae | *Abies grandis* | Mt Bachelor, USA |
| *M. rosae* Bouček | Angiosperm | Rosaceae | *Rosa* *tomentosa* Sm. | Briançon, France |
| *M. schimitscheki* Novitzki | Gymnosperm | Pinaceae | *Cedrus libani* Loud. | Kapidag, Turkey |
| *M. spermotrophus* Wachtl | Gymnosperm | Pinaceae | *Pseudotsuga mensiezii* (Mirb.) Franco | Chico, USA |
| *M. suspectus* Borries | Gymnosperm | Pinaceae | *Abies alba* Mill. | Villers-Cotterêts, France |
| *M. thyoides* Kamijo | Gymnosperm | Cupressaceae | *Chamaecyparis* sp. | Wilmington, USA |
| *M. transvaalensis* (Hussey) | Angiosperm | Anacardiaceae | *Schinus molle* L. | Marrakech, Morocco |
| *M. tsugae* Crosby | Gymnosperm | Pinaceae | *Tsuga heterophylla* (Raf.) Sarg. | Saanichton, Canada |
| *Torymus azureus* Boheman | Gymnosperm | Pinaceae | *Picea abies* (L.) Karst. | Suchora, Poland |
| *M. hypogeus* (Hussey) | Angiosperm | Anacardiaceae | *Ozoroa obovata* (Oliv.) R.&A. Fernandes | Shimba Hills, Kenya |
| *M. pistaciae* Walker | Angiosperm | Anacardiaceae | *Pistacia lentiscus* L. subsp.  e*marginata* (Engl.) AL-Saghir | Rift valley, Kenya |
| *M. somaliensis* Hussey | Gymnosperm | Cupressaceae | *Juniperus procera* Endl. | Mt Kenya Forest, Kenya |
| *M. zebrinus* Grissell | Gall-former |  | *Eucalyptus camaldulensis* | Pretoria, South Africa |
| *M. grewianae* Roques and Copeland | Angiosperm | Malvaceae | *Grewia stuhlmannii* K. Schum. | Arabuko-Sokoke forest, Kenya |
| *M. helinae* Roques and Copeland | Angiosperm | Rhamnaceae | *Helinus integrifolius* (Lam.) Kuntze | Scandent climber, Kenya |
| *M. icipeensis* Roques and Copeland | ? | ? | Malaise trap | Nairobi province, Kenya |
| *M. lanneae* Roques and Copeland | Angiosperm | Anacardiaceae | *Lannea rivae* (Chiov.) Sacl. | Machakos, Kenya |
| *M. ozoroae* Roques and Copeland | Angiosperm | Anacardiaceae | *Ozoroa insignis* Del. subsp. *reticulata* (Bak.f.) Gillett | Sindo-Mbita Road, Kenya |
